# Photoinduced Co/Ni-cocatalyzed Markovnikov hydroarylation of unactivated olefins with aryl bromides†

**DOI:** 10.1039/d4sc03355h

**Published:** 2024-08-06

**Authors:** Hong-Chao Liu, Xin-Yu Xu, Siyuan Tang, Jiawei Bao, Yu-Zhao Wang, Yiliang Chen, Xinya Han, Yong-Min Liang, Kui Zhang

**Affiliations:** a School of Chemistry and Chemical Engineering, Anhui University of Technology Ma'anshan Anhui 243002 P. R. China xinyahan@ahut.edu.cn kuizhang@ahut.edu.cn; b State Key Laboratory of Applied Organic Chemistry, Lanzhou University Lanzhou 730000 P. R. China liangym@lzu.edu.cn; c School of Pharmaceutical Sciences & Institute of Materia Medica, Shandong First Medical University & Shandong Academy of Medical Sciences Jinan 250117 Shandong China

## Abstract

Transition-metal-catalyzed hydroarylation of unactivated alkenes *via* metal hydride hydrogen atom transfer (MHAT) is an attractive approach for the construction of C(sp^2^)–C(sp^3^) bonds. However, this kind of reaction focuses mainly on using reductive hydrosilane as a hydrogen donor. Here, a novel photoinduced Co/Ni-cocatalyzed Markovnikov hydroarylation of unactivated alkenes with aryl bromides using protons as a hydrogen source has been developed. This reaction represents the first example of photoinduced MHAT *via* a reductive route intercepting an organometallic coreactant. The key to this transformation was that the Co^III^–H species was generated from the protonation of the Co^I^ intermediate, and the formed Co^III^–C(sp^3^) intermediate interacted with the organometallic coreactant rather than reacting with nucleophiles, a method which has been well developed in photoinduced Co-catalyzed MHAT reactions. This reaction is characterized by its high catalytic efficiency, construction of quaternary carbons, simple reaction conditions and expansion of the reactive mode of Co-catalyzed MHAT reactions *via* a reductive route. Moreover, this catalytic system could also be applied to complex substrates derived from glycosides.

## Abstract 2

A novel photoinduced Co/Ni-cocatalyzed Markovnikov hydroarylation of unactivated alkenes with aryl bromides by using proton as a hydrogen source has been developed for the first time.

## Graphical abstract

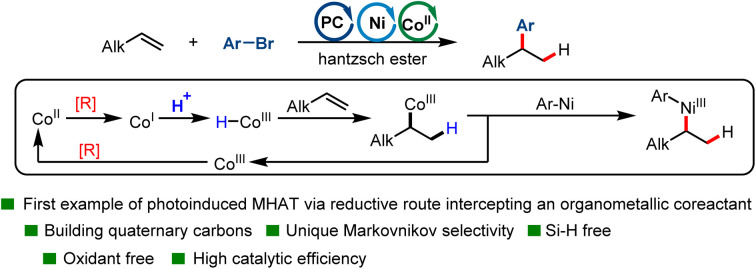

## Introduction

Unactivated alkenes are important and inexpensive starting materials in organic synthesis due to their inherent properties of stability and wide availability. The catalytic hydroarylation of unactivated alkenes has been an attractive route to alkylarenes, common structures in organic chemistry. Traditionally, hydroarylation of unactivated alkenes was accomplished either *via* a classical Friedel–Crafts reaction, a Minisci-type reaction or through methods based on metal-catalyzed C(sp^2^)–H activation of arenes.^1–6^ In recent years, a new strategy has been developed based on the reductive cross-coupling of alkenes and aryl halides in the presence of a hydride donor.^7–13^ The key advance of reductive coupling relative to previous methods is the excellent site selectivity of the arenes regardless of the substitution pattern of the starting arenes. To date, excellent work has been done on the hydroarylation of styrenes or alkenes with directing groups.^14–17^ However, this kind of reaction is a great challenge for unactivated alkenes due to the iterative migratory insertion/β-hydrogen elimination process mediated by metal hydride intermediates, which made the regioselectivity of alkenes difficult to control.^14,18–23^ To date, transition-metal-catalyzed reductive coupling hydroarylation of unactivated alkenes has occurred at the site of double bonds preinstalled on the molecules, which are scarce, and the hydride donors are limited to hydrosilane and alkoxide. Using protons as a hydrogen source for the transition-metal-catalyzed reductive coupling hydroarylation of unactivated alkenes remains underdeveloped. What is more, though a tremendous amount of endeavor has been devoted to this kind of transformation *via* thermochemistry, visible-light-induced reductive cross-coupling of unactivated alkenes and aryl halides in the presence of a hydrogen source has not yet been developed.

**Scheme 1:**
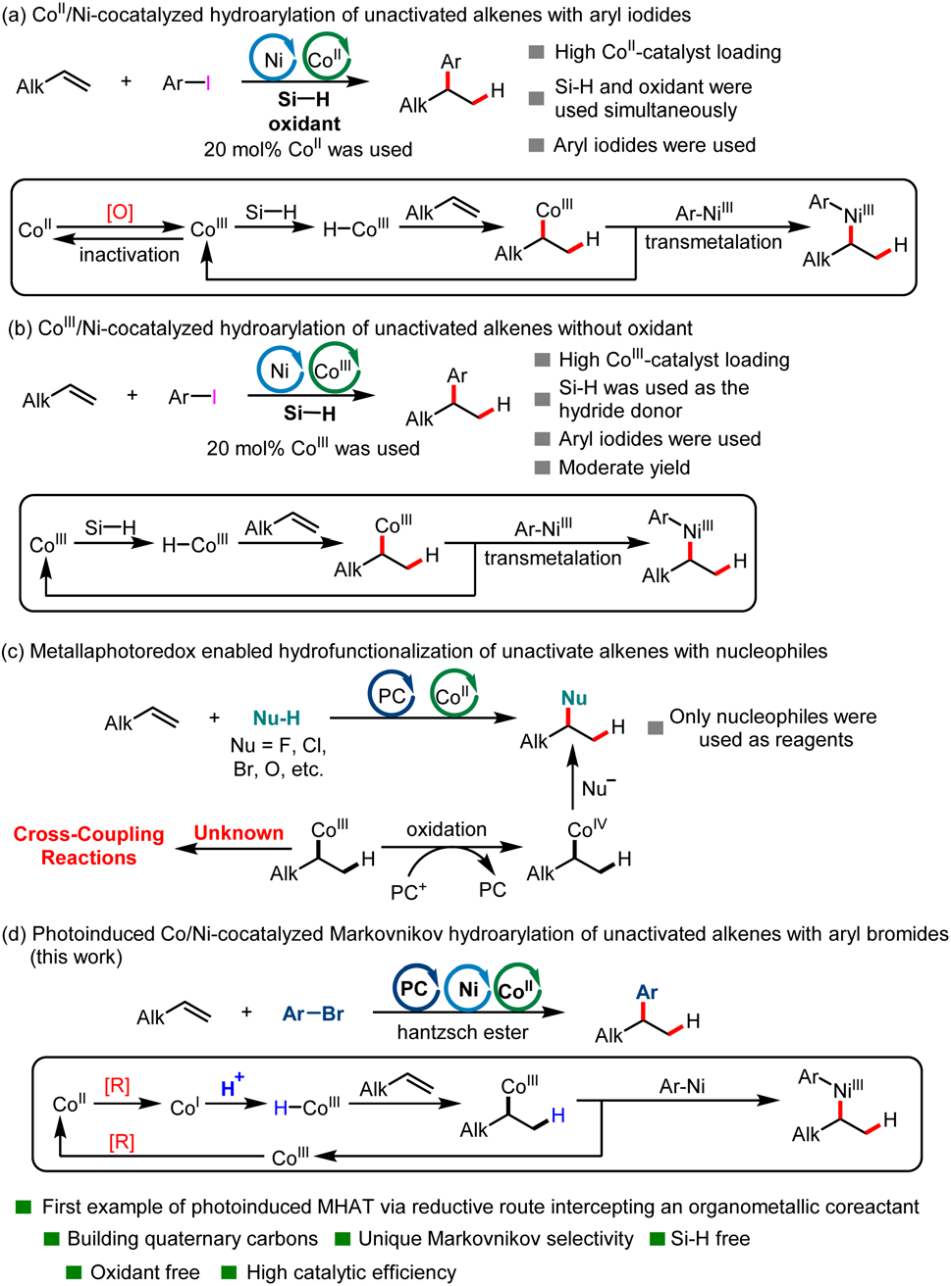
Transition-metal-catalyzed hydroarylation of unactivated alkenes and photoinduced MHAT reaction.

MHAT with unsaturated bonds has recently attracted significant attention in organic synthesis chemistry.^24–32^ This kind of reaction has enabled the successful implementation of a number of radical hydrofunctionalizations of alkenes with Markovnikov selectivity over past decades.^33–44^ In contrast to the increasing demand for reactions enabled by transition-metal hydrides (M-Hs), methods to generate them have remained limited. Generation of M-Hs relies mainly on the requirement for a pair consisting of a stoichiometric oxidant and a hydride-donor reductant. Using a Co-catalyst as an example, the former oxidized the Co^II^-precatalyst to a high-valent Co^III^ species, whereas the latter delivered an H^−^ reacting with a Co^III^ species to produce an active Co^III^–H intermediate. To date, the MHAT reaction has focused mainly on using reductive hydrosilane as a hydrogen donor. However, the combined use of oxidants and reductants complicated the reaction systems and hampered their application in process-scale reactions. What is more, methods for the transition-metal-catalyzed hydroarylation of unactivated alkenes *via* MHAT remains limited. In 2016, the Shenvi group creatively developed the first investigation of the Ni/Co^II^-cocatalyzed reductive coupling hydroarylation of unactivated terminal olefins under electrophile–electrophile conditions *via* the MHAT process to construct alkylarenes (Scheme 1a), in which reductive hydrosilane was used as the hydrogen donor and *N*-fluoropyridinium salt was used as the oxidant.^7^ This reaction represented a new strategy for the Markovnikov hydroarylation of unactivated alkenes. However, the combined use of oxidant and reductant, as well as 20 mol% Co catalysis loading, made this reaction hard to scale up. In 2018, the same group developed a new method for the hydroarylation of unactivated alkenes *via* MHAT using an Ni/Co^III^ catalytic system, in which no oxidant was needed. However, the reaction could only provide the desired product in moderate yield (Scheme 1b).^45^ In the same year, the Shenvi group developed an elegant Fe/Ni-cocatalyzed Markovnikov hydroarylation of unactivated olefins using hydrosilane as hydrogen donor, in which the substrate scope was significantly broader.^8^ However, the catalytic system was complicated (not only hydrosilane was used, but Mn or Mn/MnO_2_ were also needed for the transformation). What is more, up to now, only expensive aryl iodides have been used as arylation reagents in the hydroarylation reaction of unactivated olefins *via* MHAT.

Recently, with the development of photochemistry, a new strategy for generating Co^III^–H intermediates has been developed. The seminal work on a new method for the generation of Co^III^–H intermediates was first developed by the Matsunaga group.^46^ In the new method, Co^III^–H could be generated *via* single electron reduction of a Co^II^ precatalyst by a photoredox catalyst followed by protonation of the resultant Co^I^ intermediate.^27,46^ In this kind of reaction, the use of protons as hydrogen donors and the avoidance of oxidants provided a novel method for the MHAT reaction. In 2022, the Teskey group developed the first example of light-driven Co-catalyzed hydroarylation of styrenes using a Hantzsch ester as reductant.^47^ However, this new method generally focused on realizing the hydrofunctionalization of styrenes^47,48^ and conjugated dienes;^49–52^ the hydrofunctionalization of unactivated alkenes is rare (Scheme 1c).^53–56^ To date, the photocatalyst and MHAT catalyst cocatalyzed hydrofunctionalization of unactivated alkenes mainly employs nucleophiles as functional group reagents *via* an S_N_2 type reaction because the *in situ* formed Co^III^–C(sp^3^) intermediate could be easily oxidated to Co^IV^–C(sp^3^) by an oxidized photocatalyst.^53,54^ To date, the hydroarylation of unactivated alkenes *via* a photoinduced MHAT process using electrophilic aryl halides has not yet been developed. Continuing our research interest in C(sp^2^)–C(sp^3^) construction,^57–62^ here we disclose a novel visible-light-induced Co/Ni-cocatalyzed Markovnikov hydroarylation of unactivated alkenes with aryl bromides using a Hantzsch ester as reductant and employing protons as a hydrogen source in the absence of an organosilane reductant and oxidant (Scheme 1d). This reaction is characterized by its broad substrate scope, high catalytic efficiency, suitability for constructing quaternary carbons and mild reaction conditions. Moreover, this catalytic system could also be applied to complex substrates derived from glycosides.

## Results and discussion

We commenced on our study by using arene bromide 1a and alkene 2a as model substrates to investigate this reaction (Table 1). When this reaction was performed in the presence of 4CzIPN (2 mol%), Co(TPP) (1 mol%), Ni(dtbbpy)Br_2_ (2.5 mol%), and Hantzsch ester (1.5 equiv.) in DMAc (2 mL) under visible-light irradiation, the desired Markovnikov hydroarylation product 3aa was obtained in 82% yield at room temperature after 15 h (Table 1, entry 1). Further investigation disclosed that Co(TPP) was the pivotal MHAT catalyst to accomplish this reaction, as other Co-HAT catalysts failed to trigger the reaction (entries 2, 3). Photocatalysts were also tested and the results demonstrated that 4CzIPN was the best one in consideration of the yields and the price of the photocatalysts (entries 4–6). It is worth noting that other solvents could also trigger the reaction, though they gave a slightly lower yield, such as 1,4-dioxane (entry 8). When triethylamine was used as the reductant instead of the Hantzsch ester, no desired product 3aa was formed (entry 9). Control experiments indicated that the reaction could not provide the desired product in the absence of Co-catalyst, photocatalyst, Ni-catalyst, Hantzsch ester or light (entries 10–14).

**Table 1:** Optimization of reaction conditions^a^

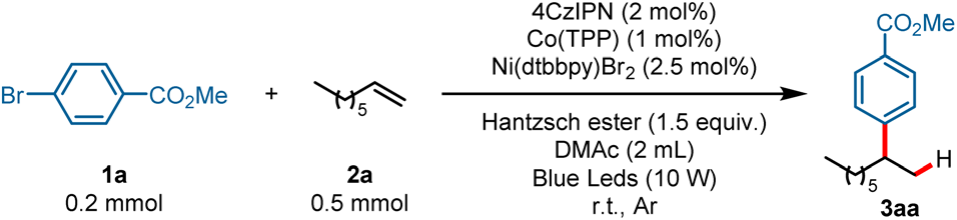		
Entry	Modified conditions	Yield of 3aa^b^ (%)
1	None	82%
2	Co–I	0
3	Co-II	0
4	Ir(dFCF_3_ppy)(dtbpy)	82%
5	Eosin Y	0
6	PC-I	0
7	DMF	81%
8	1,4-Dioxane	74%
9	Et_3_N as reductant	0
10	No light	0
11	No 4CzIPN	0
12	No Co(TPP)	0
13	No Ni(dtbbpy)Br_2_	0
14	No Hantzsch ester	0
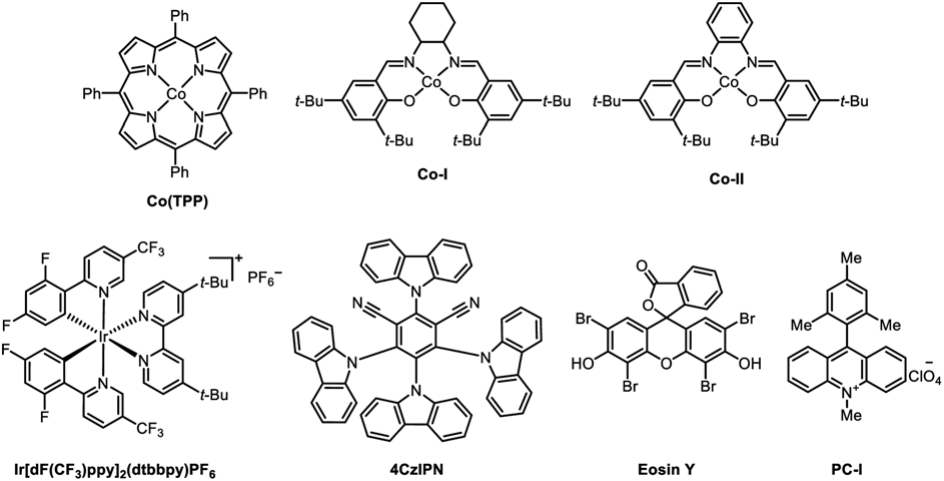		

a Reaction conditions: 1a (0.2 mmol), 2a (0.5 mmol, 2.5 equiv.), 4CzIPN (2 mol%), Co(TPP) (1 mol%), Ni(dtbbpy)Br_2_ (2.5 mol%), Hantzsch ester (0.3 mmol, 1.5 equiv.), DMAc (2 mL), blue LEDs (10 W), r.t., Ar, 15 h, cooling with a fan.

b Isolated yield. DMAc = *N*,*N*-dimethylacetamide. Hantzsch ester = diethyl-1,4-dihydro-2,6-dimethyl-3,5-pyridinedicarboxylate.

**Scheme 2:**
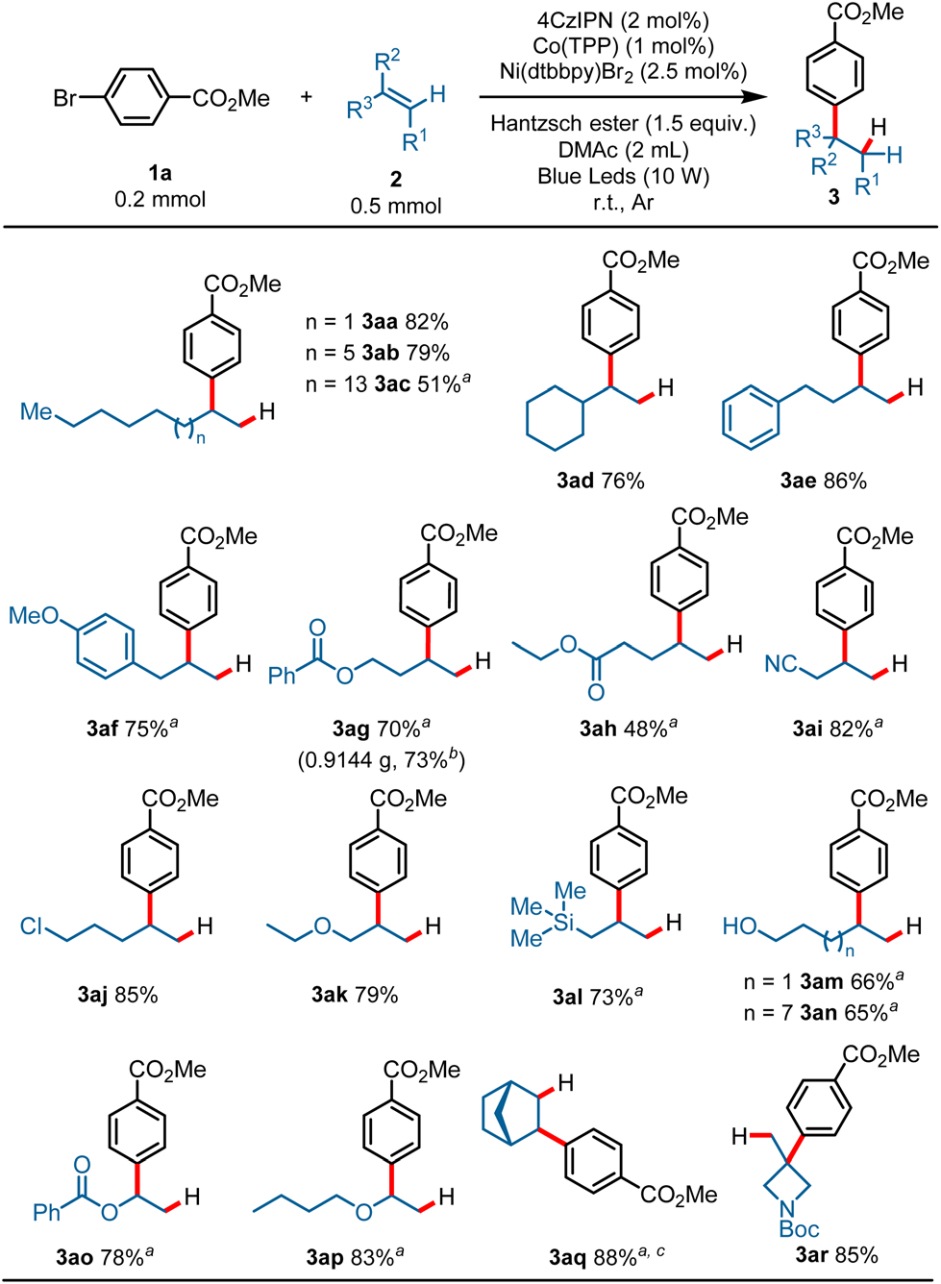
Scope of olefins. Reaction conditions: 1a (0.2 mmol), 2 (0.5 mmol, 2.5 equiv.), 4CzIPN (2 mol%), Co(TPP) (1 mol%), Ni(dtbbpy)Br_2_ (2.5 mol%), Hantzsch ester (0.3 mmol, 1.5 equiv.), DMAc (2 mL), blue LEDs, r.t., Ar, 15 h, cooling with a fan, isolated yield. ^*a*^ Ni(dtbbpy)Br_2_ (5 mol%). ^*b*^ 4.0 mmol scale, Ni(dtbbpy)Br_2_ (5 mol%) was used. ^*c*^ d.r. >20 : 1.

With the optimized reaction conditions established, the scope of the alkenes was first surveyed using methyl 4-bromobenzoate 1a as a model substrate. As shown in Scheme 2, a variety of terminal alkenes smoothly underwent the hydroarylation reaction, leading to branched alkylarenes in moderate to satisfactory yields with excellent regioselectivity. Exclusive Markovnikov selectivity was observed in all cases. A wide range of functional groups was well tolerated in this reaction. For example, alkyls (3aa–3ad, 51–82%), aryls (3ae, 86%; 3af, 75%), esters (3ag, 70%; 3ah, 48%), nitrile (3ai, 82%), halide (3aj, 85%), ether (3ak, 79%), and silicane (3al, 73%) all proved to be compatible with the reaction, and gave the target products in moderate to good yields. Product 3ac (51%) was produced in moderate yield probably due to the poor solubility of 1-eicosene in DMAc. The protonic substrates, such as alcohol, could also proceed smoothly with the transformation and gave the branched alkylarene products in moderate yields (3am, 66%; 3an, 65%). Activated alkenes proceeded well with the transformation, and gave the desired products in good yields (3ao, 78%; 3ap, 83%). To our delight, internal alkene was also compatible under the reaction conditions, as exemplified by the synthesis of 3aq in 88% yield with a high d.r. value from the corresponding norbornene. It is worth noting that 1,1-disubstituted aliphatic alkenes could also proceed with the transformation, and afforded the hydroarylation product with quaternary carbon center in good yield (3ar, 85%). This reaction was also applicable to gram-scale preparation. When this transformation was performed at the 4.0 mmol scale, the product 3ag was obtained in 73% yield on isolation (3ag, 0.9144 g) (Scheme 2).

**Scheme 3:**
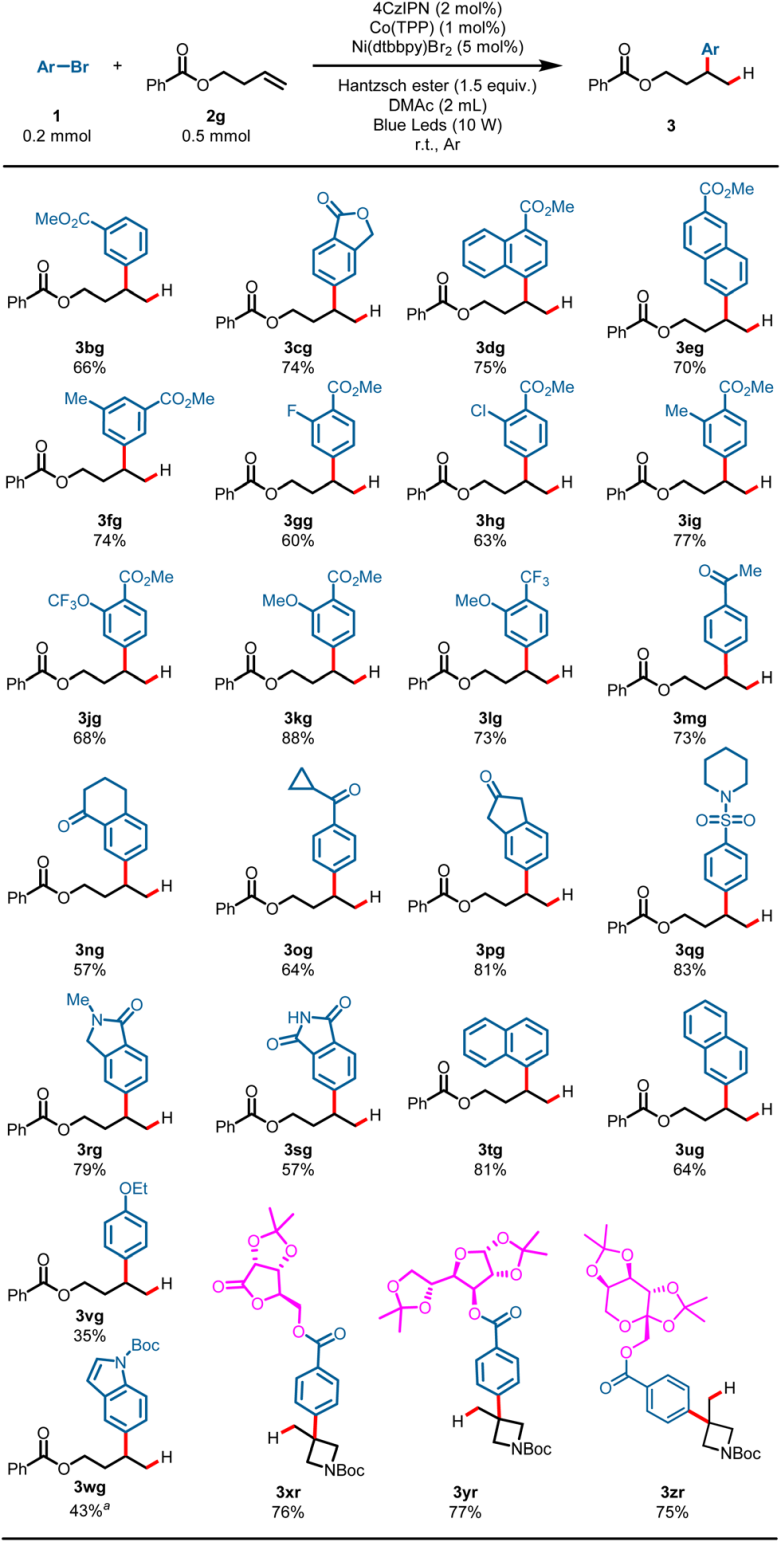
Scope of aryl bromides. Reaction conditions: 1 (0.2 mmol), 2a (0.5 mmol, 2.5 equiv.), 4CzIPN (2 mol%), Co(TPP) (1 mol%), Ni(dtbbpy)Br_2_ (5 mol%), Hantzsch ester (0.3 mmol, 1.5 equiv.), DMAc (2 mL), blue LEDs, r.t., Ar, 15 h, cooling with a fan, isolated yield. ^*a*^ Ni(dtbbpy)Br_2_ (10 mol%).

The generality of aryl bromides was then explored using but-3-en-1-yl benzoate 2g as a model substrate under standard conditions (Scheme 3). Arene electronics played a significant role in the hydroarylation reaction, plausibly due to rates of nickel oxidative addition to the aryl bromides.^7^ Substrates bearing synthetically useful electron-withdrawing substituent groups on the arenes proceeded well under these reaction conditions, and gave the products in satisfactory yields: for example, esters (3bg–3kg, 60–88%), a valuable trifluoromethyl substituent (3lg, 73%), ketones (3mg–3pg, 57–81%), and sulfamide (3qg, 83%). Phthalimide possessing an N–H bond could also survive the transformation and gave the product 3sg in 57% yield. Electron-neutral aryl bromides underwent the hydroarylation reaction well and gave the Markovnikov products in good yields (3tg, 81%; 3ug, 64%). While an electron-rich aromatic ring showed diminished yield, it was still competent in the reaction, as demonstrated by 3vg (35%). It is worth noting that heteroaryl bromide could also proceed with this reaction and gave the desired product in moderate yield (4wg, 43%) using *tert*-butyl 5-bromo-1*H*-indole-1-carboxylate as an arylation reagent. Furthermore, substrates derived from multiple-functionalized glycosides smoothly converted into the desired products with quaternary carbons, demonstrating the robustness of this hydroarylation reaction (3xr–3zr, 75–77%).

**Scheme 4:**
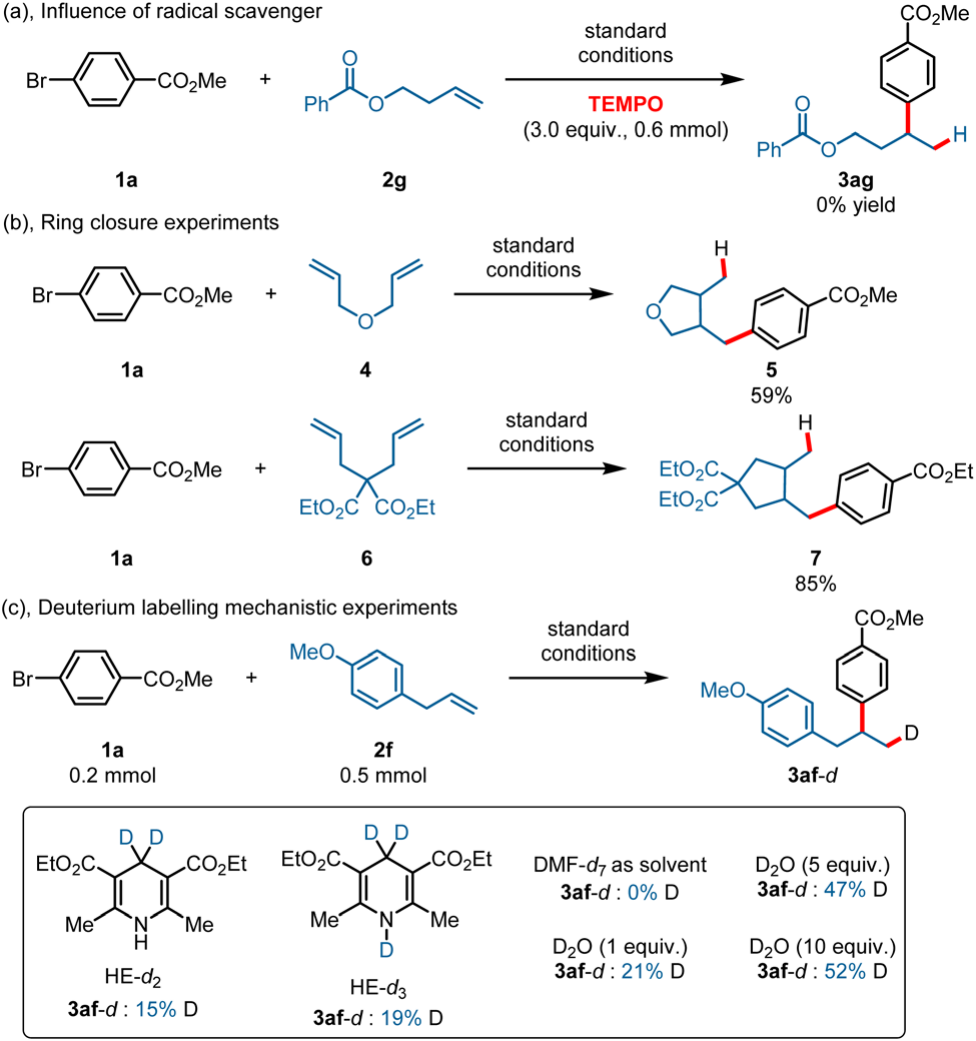
Mechanistic study experiments.

To gain more mechanistic information about this transformation, various mechanistic study experiments were conducted. The reaction was completely inhibited when a stoichiometric amount of radical scavenger (TEMPO) was added under the standard conditions (Scheme 4a). Ring-closure experiments were also conducted using 3-(allyloxy)prop-1-ene 4 and diethyl 2,2-diallylmalonate 6 as substrates, and the corresponding ring-closure products 5 and 7 were obtained in 59% and 85% yields (Scheme 4b). In addition, deuterium labelling studies were also conducted with HE-*d*_2_ and HE-*d*_3_ (Scheme 4c), and a deuteron was found in the products in all cases. The product contained no deuterons when DMF-*d*_7_ was used as the solvent (here, we used DMF-*d*_7_ instead of DMAc-*d*_9_). What is more, a deuteron was present in the products when using D_2_O as the D^+^ source under standard reaction conditions, and the results confirmed that a Co^III^–H species was generated through reduction of H^+^ by Co^I^.^50,52^ Stern–Volmer fluorescence quenching experiments were also conducted (ESI, 5.5† Stern–Volmer fluorescence quenching experiments), and the results showed that both HE and Co(TPP) could quench the excited state of 4CzIPN, whereas Ni(dtbbpy)Br_2_ showed only a slight quenching ability. However, the quenching rate constant of HE is larger than that of Co(TPP), suggesting a reductive quenching pathway. We also conducted a quenching experiment for Co(TPP) with Hantzsch ester, and the result indicated that the Hantzsch ester showed only a slight quenching ability for Co(TPP) under an excitation wavelength of 440 nm.

**Scheme 5:**
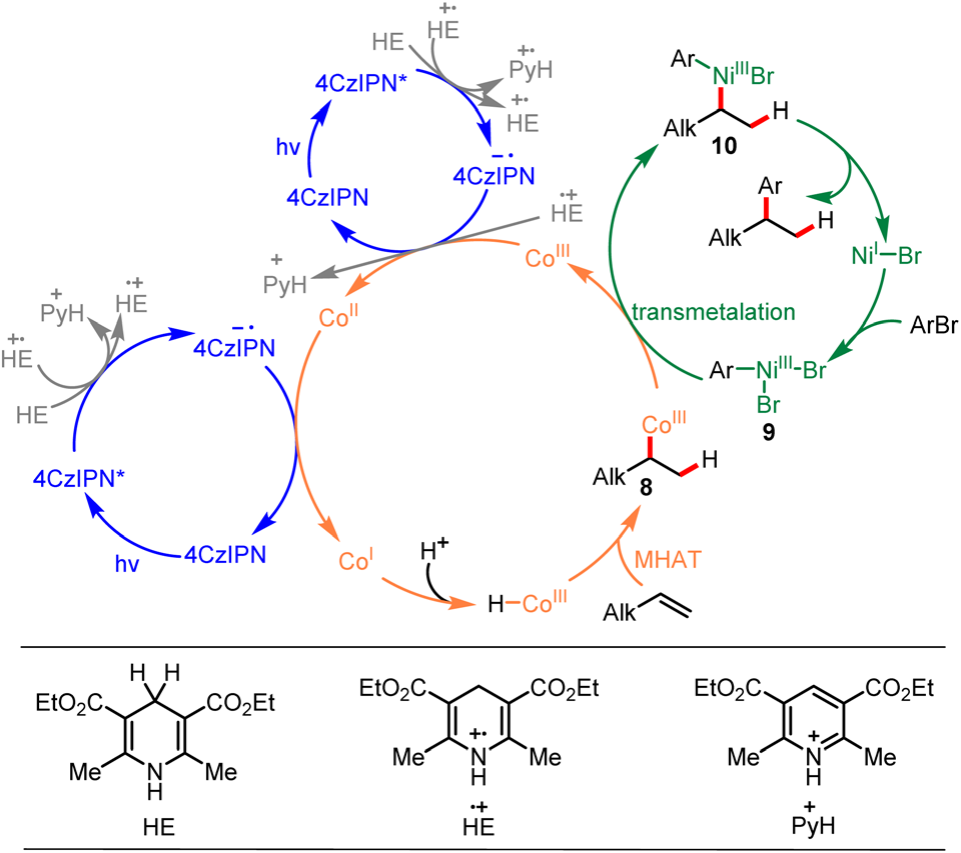
Proposed mechanism.

Based on the mechanistic study experiments and previous reports,^7,14,23,45,50,51^ a mechanism for this reaction was proposed, as depicted in Scheme 5. The reaction commenced by single electron transfer (SET) from the Hantzsch ester [HE (*E*_HE_˙^+^/_HE_ = +1.0 V *vs.* SCE)]^63^ to the photoexcited 4CzIPN* [*E*_1/2_ (4CzIPN*/4CzIPN˙^−^) = +1.43 V *vs.* SCE]^64^ generating the corresponding radical ions 4CzIPN˙^−^ and HE˙^+^. The produced HE˙^+^ could further participate in electron transfer events and produced pyH^+^. The strong reductant 4CzIPN˙^−^ [*E*_1/2_ (4CzIPN/4CzIPN˙^−^) = −1.24 V *vs.* SCE]^64^ reduced Co^III^ to Co^II^ [*E*_1/2_ (Co^III^/Co^II^) = +0.03 V *vs.* SCE]^27^ and then reduced Co^II^ to Co^I^ [*E*_1/2_ (Co^II^/Co^I^) = −0.87 V *vs.* SCE].^27^ The Co^III^–H species was formed through protonation of Co^I^ with the reaction media, and the formed Co^III^–H species underwent Markovnikov MHAT with alkene forming C(sp^3^)–Co^III^ species 8. Meanwhile, the Ni^II^ precatalyst was reduced to Ni^I^ [*E*_1/2_ (Ni^II^/Ni^I^) = −0.66 V *vs.* SCE]^65^ by reaction media, and Ni^I^ underwent oxidative addition with aryl bromide, forming Ar-Ni^III^ intermediate 9. Then, transmetalation between C(sp^3^)–Co^III^ species 8 and Ar–Ni^III^ intermediate 9 generated Co^III^ species and Ni^III^ intermediate 10. Ultimately, Ni^III^ intermediate 10 underwent reductive elimination, forming the desired product and regenerating the Ni^I^ intermediate. An alternative mechanism for this reaction proceeding *via* a radical pathway is also presented in ESI (ESI, 6.† An alternative mechanism).

## Conclusions

In conclusion, we have developed a novel photoinduced Co/Ni-cocatalyzed Markovnikov hydroarylation of unactivated alkenes with aryl bromides using protons as a hydrogen source in the absence of reductive hydrosilane for the first time. This reaction represents a novel example of employing protons as a hydrogen source in the photoinduced transition-metal-catalyzed reductive cross-coupling of alkenes with aryl halides. The key to this transformation was that Co(iii)–H was generated from protonation of the Co^I^ intermediate formed *via* a reductive route and the formed Co^III^–C(sp^3^) intermediate interacted with the organometallic coreactant rather than reacting with nucleophiles, a method which has been well developed in the photoinduced Co-catalyzed MHAT reactions. This reaction worked well with a broad range of aryl bromides as well as alkenes with high catalytic efficiency under mild reaction conditions. This reaction was suitable for building quaternary carbons, and was compatible with complex substrates derived from glycosides.

## Author contributions

Methodology, H.-C. L.; investigation, X.-X. Y.; writing original draft, H.-C. L.; writing review & editing, S. T., J. B., Y.-Z. W., Y. C., X. H., Y.-M. L. and K. Z.; funding acquisition, X. H.; supervision, X. H., Y.-M. L. and K. Z. All authors co-wrote the paper, discussed the results, analyzed the data and commented on the paper.

## Conflicts of interest

The authors declare no competing interests.

## Supplementary Material

SC-015-D4SC03355H-s001

## Data Availability

The data supporting this article have been included as part of the ESI.†
