# Supplementary material for: Photoinduced Co/Ni-cocatalyzed Markovnikov hydroarylation of unactivated olefins with aryl bromides
Source: Chem Sci. 2024 Aug 6;15(36):14865–71. doi: 10.1039/d4sc03355h (PMC11342154; doi:10.1039/d4sc03355h)

Supplementary Information (SI) for Chemical Science.
This journal is © The Royal Society of Chemistry 2024

Photoinduced Co/Ni-cocatalyzed Markovnikov Hydroarylation of Unactivated Olefins with Aryl Bromides

Hong-Chao Liu,‡[a] Xin-Yu Xu,‡[a] Siyuan Tang,[a] Jiawei Bao,[a] Yu-Zhao Wang,[c] Yiliang Chen,[a] Xinya Han,*[a] Yong-Min Liang*[b] and Kui Zhang*[a]

[a]School of Chemistry and Chemical Engineering, Anhui University of Technology, Ma'anshan, Anhui 243002, P. R. China

[b]State Key Laboratory of Applied Organic Chemistry and College of Chemistry and Chemical Engineering, Lanzhou University, Lanzhou 730000, P.R. China

[c]School of Pharmaceutical Sciences & Institute of Materia Medica, Shandong First Medical University & Shandong Academy of Medical Sciences, Jinan 250117, Shandong, China.

Table of Contents

1. General information S1

2. The optimization of the reaction S2

3. Unsuccessful substrates S3

4. Synthesis and characterization of products S3

5. Mechanism study experiments..S23

6. An Alternative Mechanism S35

7. References...S35

8. ^{1}H, ^{13}C and ^{19}F NMR spectra of all products S36

1. General information

Unless otherwise noted, all these reactions were carried out under nitrogen atmosphere. For column chromatography, silica gel (200-300 mesh) was employed. Solvent was freshly distilled prior to use unless otherwise noted. Organic solvents were concentrated under reduced pressure using a rotary evaporator.

Instrumentation. Deuterated solvents were purchased from Cambridge Isotope Laboratories. ^{1}H NMR spectra were recorded on Bruker AVANCE III 400 or Bruker AVANCE III HD 400 with 400 MHz frequencies, and ^{13}C NMR spectra were recorded on Bruker AVANCE III 400 or Bruker AVANCE III HD 400 with 101 MHz frequencies. ^{19}F NMR spectra were recorded on a Bruker AVANCE III HD 400 spectrometer with a ^{19}F operating frequency of 376 MHz. Chemical shifts (ppm) were recorded with TMS (tetramethylsilane) as the internal reference standard. Chemical shifts (δ) were reported in ppm relative to the residual solvent signal (TMS δ = 0 for ^{1}H NMR and $CDCl_3$ δ = 77.0 for ^{13}C NMR). Multiplicities are given as s (singlet), d (doublet), t (triplet), dd (doublet of doublets), td (triplet of doublets) or m (multiplet). HRMS obtained using a Q-TOF instrument equipped with an ESI source. All photochemical reactions were conducted using a blue light-emitting diode (LED) as the visible-light source (440 nm, Kessil LEDs lights).

Materials. Unless otherwise noted below, all other compounds have been reported in the literature or are commercially available. Commercial reagents were used without further purification. The HE-d_2 and HE-d_3 were synthesized according to reported literatures.[1, 2] The substrates of **3xr**, **3yr** and **3zr** were derived from 2,3-*O*-Isopropylidene-*D*-ribonic gamma-lactone (CAS number: 30725-00-9), Diacetone-D-glucose (CAS number: 14686-89-6) and Diacetonefructose (CAS number: 20880-92-6) respectively.

2. The optimization of the reaction[a]

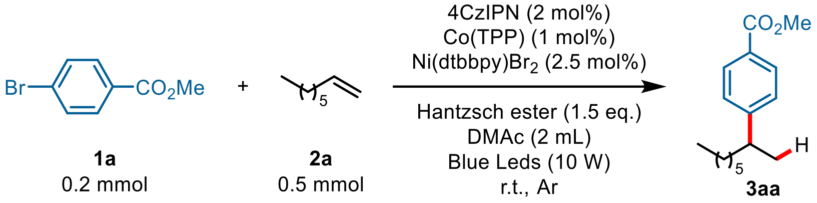

entry	modified conditions	yield of **3aa** (%)[b]
**1**	**none**	**82%**
2	Co-I	0
3	Co-II	0
4	Ir(dFCF3ppy)(dtbpy)	82%
5	Eosin Y	0
6	PC-I	0
7	DMF	81%
8	1,4-dioxane	74%
9	Et_3N as reductant	0
10	No light	0
11	No 4CzIPN	0
12	No Co(TPP)	0
13	No $Ni(dtbbpy)Br_2$	0
14	No Hantzsch ester	0

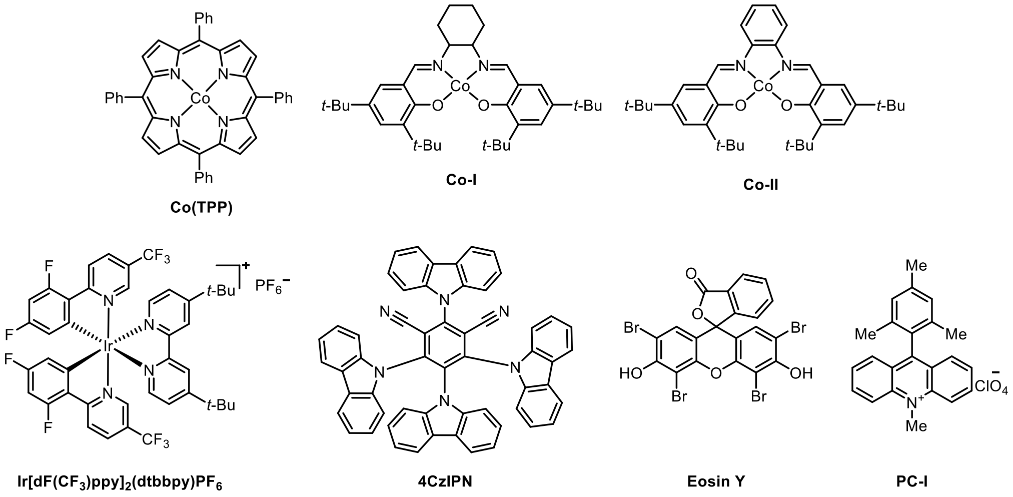

[a] Reaction conditions: **1a** (0.2 mmol), **2a** (0.5 mmol, 2.5 equiv), 4CzIPN (2 mol%), Co(TPP) (1 mol%), $Ni(dtbbpy)Br_2$ (2.5 mol%), Hantzsch ester (0.3 mmol, 1.5 eq.), DMAc (2 mL), Blue Leds

(10 W), r.t., Ar, 15 h, cooling with a fan. [b] Isolated yield. DMAc = *N*,*N*-Dimethylacetamide. Hantzsch ester = Diethyl 1,4-dihydro-2,6-dimethyl-3,5-pyridinedicarboxylate.

3. Unsuccessful substrates

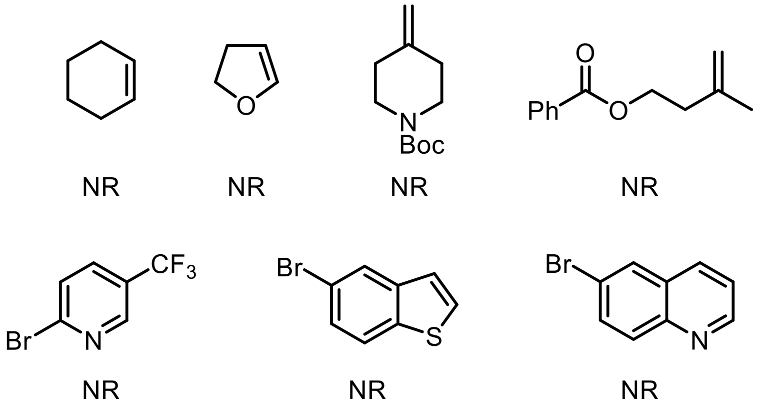

4. Synthesis and characterization of products

4.1 General Procedures I (GPI)

In an oven-dried 10 mL tube equipped with a stirring bar, aryl bromide compounds (0.2 mmol, 1.0 equiv.), unactivated olefins (0.5 mmol, 2.5 equiv.), diethyl 1,4-dihydro-2,6-dimethyl-3,5-pyridinedicarboxylate (Hantzsch ester, 0.3 mmol, 1.5 equiv.), 4CzIPN (0.004 mmol, 2 mol%), Ni(dtbbpy)Br_2 (0.005 mmol, 2.5 mol%), and Co(TPP) (0.002 mmol, 1 mol%) were added. The tube was charged with nitrogen (repeated three times), then *N*,*N*-Dimethylacetamide (DMAc, 2 mL) was injected. The resulting suspension was stirred at room temperature and irradiated with blue LEDs (440 nm, 10 W) by cooling with a fan for 15 h. After completion, the resulting mixture was quenched with H_2O (30 mL). Then the mixture was extracted with ethyl acetate (30 mL). The organic layer was washed with brine (30 mL × 2), dried over anhydrous Na_2SO_4, and concentrated in vacuum. The residue was purified by flash chromatography on silica gel to afford the desired products.

4.2 General Procedures II (GPII)

In an oven-dried 10 mL tube equipped with a stirring bar, aryl bromide compounds (0.2 mmol, 1.0 equiv.), unactivated olefins (0.5 mmol, 2.5 equiv.), Hantzsch ester (0.3 mmol, 1.5 equiv.), 4CzIPN (0.004 mmol, 2 mol%), Ni(dtbbpy)Br_2 (0.01 mmol, 5 mol%), and Co(TPP) (0.002 mmol, 1 mol%) were added. The tube was charged with nitrogen (repeated three times), then DMAc (2 mL) was injected. The resulting

suspension was stirred at room temperature and irradiated with blue LEDs (440 nm, 10 W) by cooling with a fan for 15 h. After completion, the resulting mixture was quenched with H_2O (30 mL). Then the mixture was extracted with ethyl acetate (30 mL). The organic layer was washed with brine (30 mL × 2), dried over anhydrous Na_2SO_4, and concentrated in vacuum. The residue was purified by flash chromatography on silica gel to afford the desired products.

4.3 Gram-scale reaction procedures

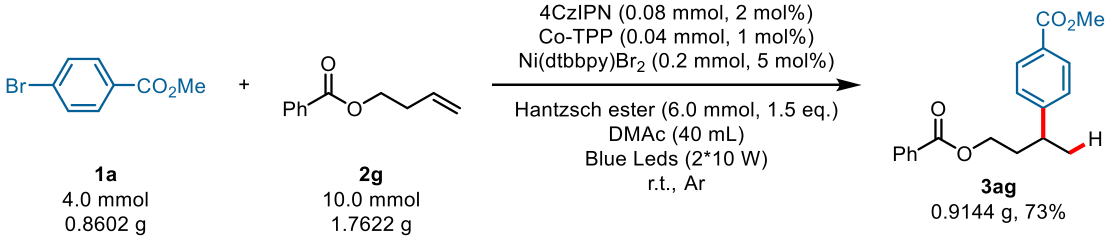

In an oven-dried 100 mL tube equipped with a stirring bar, **1a** (4.0 mmol, 1 equiv.), **2g** (10 mmol, 2.5 equiv.), Hantzsch ester (6.0 mmol, 1.5 equiv.), 4CzIPN (0.08 mmol, 2 mol%), Ni(dtbbpy)Br_2 (0.2 mmol, 5 mol%), and Co(TPP) (0.04 mmol, 1 mol%) were added. The tube was charged with nitrogen (repeated three times), then DMAc (40.0 mL) was injected. The resulting suspension was stirred at room temperature and irradiated with blue LEDs (440 nm, 2*10 W) by cooling with a fan for 15 h. After completion, the resulting mixture was quenched with H_2O (100 mL). Then the mixture was extracted with ethyl acetate (100 mL). The organic layer was washed with brine (100 mL × 2), dried over anhydrous Na_2SO_4, and concentrated in vacuum. The residue was purified by flash chromatography on silica gel to afford the desired product **3ag** (0.9144 g, 73%).

4.4 Characterization of products

methyl 4-(octan-2-yl)benzoate (3aa)

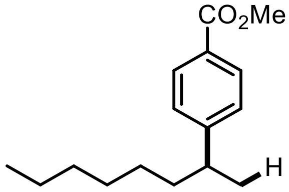

GPI, 41.0 mg, 82% yield. ^{1}H NMR (400 MHz, $CDCl_3$) δ 7.96 (d, J = 8.3 Hz, 2H), 7.24 (d, J = 8.3 Hz, 2H), 3.89 (s, 3H), 2.78 – 2.68 (m, 1H), 1.62 – 1.52 (m, 2H), 1.29

– 1.19 (m, 10H), 1.16 – 1.07 (m, 1H), 0.85 (t, J = 6.8 Hz, 3H). ^{13}C NMR (101 MHz, $CDCl_3$) δ 167.1, 153.5, 129.6, 127.7, 127.0, 51.9, 40.0, 38.1, 31.7, 29.3, 27.5, 22.6, 22.0, 14.0. HRMS (ESI-TOF) (m/z): Calcd for $C_{16}H_{25}O_2$ ([M + H]$^+$): 249.1849; found: 249.1848.

methyl 4-(dodecan-2-yl)benzoate (3ab)

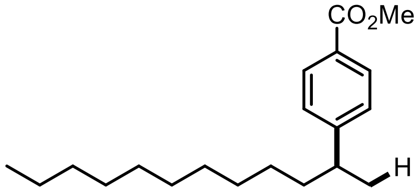

GPI, 48.2 mg, 79% yield. ^{1}H NMR (400 MHz, $CDCl_3$) δ 7.98 – 7.93 (m, 2H), 7.26 – 7.22 (m, 2H), 3.90 (s, 3H), 2.78 – 2.68 (m, 1H), 1.60 – 1.51 (m, 2H), 1.28 – 1.18 (m, 18H), 1.16 – 1.08 (m, 1H), 0.87 (t, J = 6.9 Hz, 3H).^{13}C NMR (101 MHz, $CDCl_3$) δ 167.2, 153.5, 129.6, 127.7, 127.0, 51.9, 40.0, 38.1, 31.9, 29.6, 29.6, 29.6, 29.5, 29.3, 27.6, 22.7, 22.0, 14.1. HRMS (ESI-TOF) (m/z): Calcd for $C_{20}H_{33}O_2$ ([M + H]$^+$): 305.2475; found: 305.2475.

methyl 4-(icosan-2-yl)benzoate (3ac)

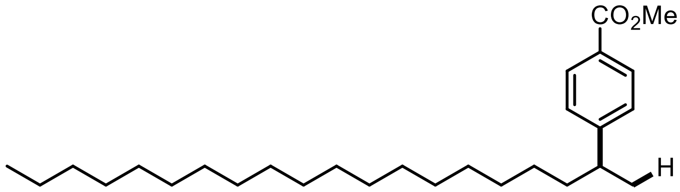

GPII, 42.9 mg, 51% yield. ^{1}H NMR (400 MHz, $CDCl_3$) δ 7.95 (d, J = 8.0 Hz, 2H), 7.24 (d, J = 8.0 Hz, 2H), 3.90 (s, 3H), 2.78 – 2.68 (m, 1H), 1.62 – 1.51 (m, 3H), 1.28 – 1.19 (m, 34H), 0.88 (t, J = 6.7 Hz, 3H). ^{13}C NMR (101 MHz, $CDCl_3$) δ 167.2, 153.5, 129.7, 127.7, 127.0, 51.9, 40.1, 38.2, 31.9, 30.1 – 29.1 (m), 27.6, 22.7, 22.1, 14.1. HRMS (ESI-TOF) (m/z): Calcd for $C_{28}H_{49}O_2$ ([M + H]$^+$): 417.3727; found: 417.3727.

methyl 4-(1-cyclohexylethyl)benzoate (3ad)

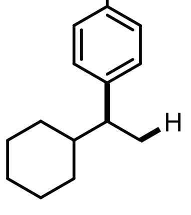

GPI, 37.6 mg, 76% yield. ^{1}H NMR (400 MHz, $CDCl_3$) δ 7.99 – 7.90 (m, 2H), 7.24 – 7.16 (m, 2H), 3.90 (s, 3H), 2.55 – 2.44 (m, 1H), 1.87 (m, 1H), 1.78 – 1.70 (m, 1H), 1.67 – 1.55 (m, 2H), 1.47 – 1.35 (m, 2H), 1.26 – 1.19 (m, 4H), 1.14 – 1.04 (m, 2H), 0.99 – 0.87 (m, 1H), 0.85 – 0.73 (m, 1H). ^{13}C NMR (101 MHz, $CDCl_3$) δ 167.2, 152.7, 129.4, 127.7, 127.7, 51.9, 46.0, 44.0, 31.4, 30.5, 26.4, 18.6. HRMS (ESI-TOF) (m/z): Calcd for $C_{16}H_{23}O_2$ ($[M + H]^+$): 247.1693; found: 247.1693.

methyl 4-(4-phenylbutan-2-yl)benzoate (3ae)

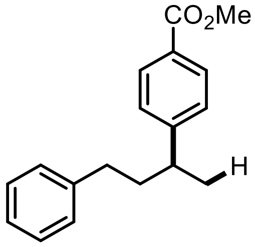

GPI, 46.5 mg, 86% yield. ^{1}H NMR (400 MHz, $CDCl_3$) δ 8.01 – 7.96 (m, 2H), 7.29 – 7.22 (m, 4H), 7.19 – 7.14 (m, 1H), 7.13 – 7.08 (m, 2H), 3.90 (s, 3H), 2.83 – 2.72 (m, 1H), 2.55 – 2.42 (m, 2H), 1.97 – 1.87 (m, 2H), 1.28 (d, J = 6.9 Hz, 3H). ^{13}C NMR (101 MHz, $CDCl_3$) δ 167.1, 152.7, 142.1, 129.8, 128.3, 127.9, 127.1, 125.7, 51.9, 39.6, 39.5, 33.7, 22.2. HRMS (ESI-TOF) (m/z): Calcd for $C_{18}H_{21}O_2$ ($[M + H]^+$): 269.1536; found: 269.1537.

methyl 4-(1-(4-methoxyphenyl)propan-2-yl)benzoate (3af)

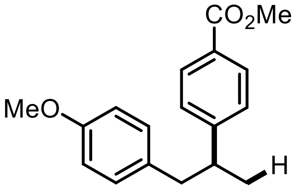

GPII, 43.1 mg, 75% yield. ^{1}H NMR (400 MHz, $CDCl_3$) δ 7.95 – 7.91 (m, 2H), 7.23 – 7.17 (m, 2H), 6.96 – 6.90 (m, 2H), 6.78 – 6.72 (m, 2H), 3.89 (s, 3H), 3.75 (s, 3H), 3.06 – 2.95 (m, 1H), 2.88 – 2.70 (m, 2H), 1.25 (d, J = 6.9 Hz, 3H). ^{13}C NMR (101 MHz, $CDCl_3$) δ 167.1, 157.8, 152.3, 132.2, 129.9, 129.6, 127.9, 127.1, 113.4, 55.1, 52.0, 43.8, 42.1, 21.0. HRMS (ESI-TOF) (m/z): Calcd for $C_{18}H_{20}O_3Na$ ($[M + Na]^+$): 307.1305; found: 307.1304.

methyl 4-(4-(benzoyloxy)butan-2-yl)benzoate (3ag)

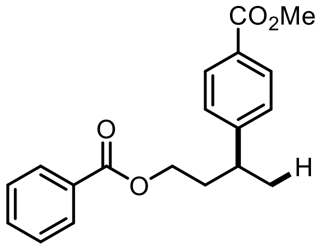

GPII, 44.1 mg, 70% yield. ^{1}H NMR (400 MHz, $CDCl_3$) δ 8.00 – 7.94 (m, 4H), 7.58 – 7.52 (m, 1H), 7.42 (t, J = 7.6 Hz, 2H), 7.30 (d, J = 8.0 Hz, 2H), 4.32 – 4.25 (m, 1H), 4.22 – 4.14 (m, 1H), 3.90 (s, 3H), 3.07 – 2.96 (m, 1H), 2.12 – 2.05 (m, 2H), 1.35 (d, J = 7.0 Hz, 3H). ^{13}C NMR (101 MHz, $CDCl_3$) δ 167.0, 166.4, 151.7, 132.9, 130.2, 129.9, 129.5, 128.3, 128.2, 127.0, 63.2, 52.0, 37.1, 36.7, 22.1. HRMS (ESI-TOF) (m/z): Calcd for $C_{19}H_{20}O_4Na$ ($[M + Na]^+$): 335.1254; found: 335.1252.

methyl 4-(5-ethoxy-5-oxopentan-2-yl)benzoate (3ah)

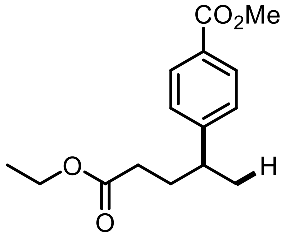

GPII, 25.8 mg, 48% yield. ^{1}H NMR (400 MHz, $CDCl_3$) δ 7.97 (d, J = 8.3 Hz, 2H), 7.28 – 7.23 (m, 2H), 4.09 (q, J = 7.1 Hz, 2H), 3.91 (s, 3H), 2.84 – 2.73 (m, 1H), 2.24 – 2.10 (m, 2H), 2.02 – 1.85 (m, 2H), 1.28 (d, J = 6.8 Hz, 3H), 1.23 (t, J = 7.1 Hz, 3H). ^{13}C NMR (101 MHz, $CDCl_3$) δ 173.4, 167.0, 151.8, 129.8, 128.2, 127.0, 60.3, 52.0, 39.4, 32.9, 32.4, 21.8, 14.2. HRMS (ESI-TOF) (m/z): Calcd for $C_{15}H_{20}O_4Na$ ($[M + Na]^+$): 287.1254; found: 287.1255.

methyl 4-(1-cyanopropan-2-yl)benzoate (3ai)

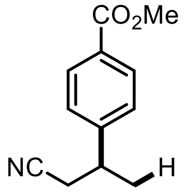

GPII, 33.6 mg, 82% yield. ^{1}H NMR (400 MHz, $CDCl_3$) δ 8.05 – 7.99 (m, 2H), 7.35 – 7.29 (m, 2H), 3.91 (s, 3H), 3.28 – 3.18 (m, 1H), 2.68 – 2.54 (m, 2H), 1.47 (d, J = 7.0 Hz, 3H). ^{13}C NMR (101 MHz, $CDCl_3$) δ 166.6, 148.1, 130.1, 129.3, 126.6, 118.1, 52.1, 36.5, 26.0, 20.5. HRMS (ESI-TOF) (m/z): Calcd for $C_{12}H_{13}NO_2Na$ ($[M + Na]^+$):

226.0839; found: 226.0838.

methyl 4-(5-chloropentan-2-yl)benzoate (3aj)

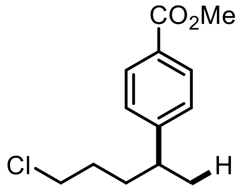

GPI, 41.0 mg, 85% yield. ^{1}H NMR (400 MHz, $CDCl_3$) δ 7.97 (d, *J* = 8.1 Hz, 2H), 7.25 (d, *J* = 8.2 Hz, 2H), 3.90 (s, 3H), 3.50 – 3.45 (m, 2H), 2.82 – 2.72 (m, 1H), 1.79 – 1.68 (m, 3H), 1.65 – 1.55 (m, 1H), 1.28 (d, *J* = 6.9 Hz, 3H). ^{13}C NMR (101 MHz, $CDCl_3$) δ 167.0, 152.3, 129.8, 128.1, 126.9, 52.0, 45.0, 39.5, 35.1, 30.6, 22.1. HRMS (ESI-TOF) (m/z): Calcd for $C_{13}H_{18}ClO_2$ ([M + H]$^+$): 241.0990; found: 241.0990.

methyl 4-(1-ethoxypropan-2-yl)benzoate (3ak)

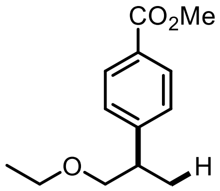

GPI, 35.5 mg, 79% yield. ^{1}H NMR (400 MHz, $CDCl_3$) δ 8.0 (d, *J* = 8.1 Hz, 2H), 7.3 (d, *J* = 8.0 Hz, 2H), 3.9 (s, 3H), 3.6 – 3.5 (m, 1H), 3.5 – 3.4 (m, 3H), 3.1 – 3.0 (m, 1H), 1.3 (d, *J* = 7.0 Hz, 3H), 1.2 (t, *J* = 7.0 Hz, 3H). ^{13}C NMR (101 MHz, $CDCl_3$) δ 167.1, 150.1, 129.6, 128.2, 127.4, 75.9, 66.4, 52.0, 40.1, 18.2, 15.1. HRMS (ESI-TOF) (m/z): Calcd for $C_{13}H_{19}O_3$ ([M + H]$^+$): 223.1329; found: 223.1329.

methyl 4-(1-(trimethylsilyl)propan-2-yl)benzoate (3al)

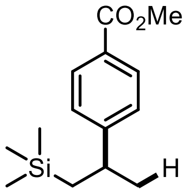

GPII, 36.7 mg, 73% yield. ^{1}H NMR (400 MHz, $CDCl_3$) δ 7.98 – 7.92 (m, 2H), 7.30 – 7.25 (m, 2H), 3.90 (s, 3H), 2.97 – 2.88 (m, 1H), 1.28 (d, *J* = 6.9 Hz, 3H), 1.02 – 0.87 (m, 2H), -0.12 (s, 9H). ^{13}C NMR (101 MHz, $CDCl_3$) δ 167.1, 155.3, 129.7, 127.7, 126.7, 51.9, 36.5, 26.7, 26.1, -1.0. HRMS (ESI-TOF) (m/z): Calcd for $C_{14}H_{23}O_2Si$ ([M + H]$^+$): 251.1462; found: 251.1462.

methyl 4-(5-hydroxypentan-2-yl)benzoate (3am)

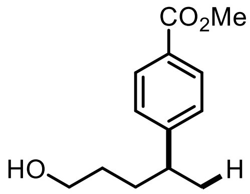

GPII, 29.7 mg, 66% yield. ^{1}H NMR (400 MHz, $CDCl_3$) δ 7.96 (d, *J* = 8.1 Hz, 2H), 7.25 (d, *J* = 8.1 Hz, 2H), 3.90 (s, 3H), 3.59 (t, *J* = 6.5 Hz, 2H), 2.82 – 2.71 (m, 1H), 1.69 – 1.60 (m, 3H), 1.55 – 1.48 (m, 1H), 1.44 – 1.37 (m, 1H), 1.27 (d, *J* = 6.9 Hz, 3H)· ^{13}C NMR (101 MHz, $CDCl_3$) δ 167.1, 152.8, 129.7, 127.9, 127.0, 62.8, 52.0, 39.9, 34.1, 30.7, 22.1. HRMS (ESI-TOF) (m/z): Calcd for $C_{13}H_{18}O_3Na$ ([M + Na]$^+$): 245.1148; found: 245.1150.

methyl 4-(11-hydroxyundecan-2-yl)benzoate (3an)

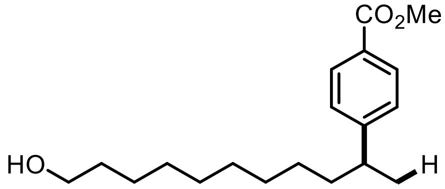

GPII, 40.2 mg, 65% yield. ^{1}H NMR (400 MHz, $CDCl_3$) δ 7.98 – 7.93 (m, 2H), 7.26 – 7.21 (m, 2H), 3.90 (s, 3H), 3.62 (t, *J* = 6.6 Hz, 2H), 2.78 – 2.68 (m, 1H), 1.61 – 1.50 (m, 5H), 1.32 – 1.20 (m, 14H), 1.15 – 1.06 (m, 1H). ^{13}C NMR (101 MHz, $CDCl_3$) δ 167.2, 153.4, 129.6, 127.7, 127.0, 63.0, 51.9, 40.0, 38.1, 32.7, 30.3 – 28.8 (m), 27.5, 25.7, 22.0. HRMS (ESI-TOF) (m/z): Calcd for $C_{19}H_{31}O_3$ ([M + H]$^+$): 307.2268; found: 307.2267.

methyl 4-(1-(benzoyloxy)ethyl)benzoate (3ao)

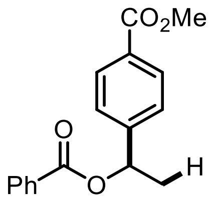

GPII, 44.9 mg, 78% yield. ^{1}H NMR (400 MHz, $CDCl_3$) δ 8.11 – 8.02 (m, 4H), 7.60 – 7.54 (m, 1H), 7.53 – 7.43 (m, 4H), 6.16 (q, *J* = 6.6 Hz, 1H), 3.91 (s, 3H), 1.68 (d, *J* = 6.6 Hz, 3H). ^{13}C NMR (101 MHz, $CDCl_3$) δ 166.7, 165.6, 146.8, 133.1, 130.1, 129.9, 129.6, 128.4, 125.8, 72.3, 52.1, 22.4. HRMS (ESI-TOF) (m/z): Calcd for $C_{17}H_{16}O_4Na$ ([M + Na]$^+$): 307.0941; found: 307.0939.

methyl 4-(1-butoxyethyl)benzoate (3ap)

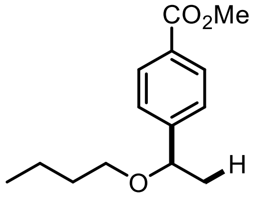

GPII, 39.3 mg, 83% yield. ^{1}H NMR (400 MHz, $CDCl_3$) δ 8.04 – 7.99 (m, 2H), 7.41 – 7.36 (m, 2H), 4.43 (q, *J* = 6.5 Hz, 1H), 3.91 (s, 3H), 3.35 – 3.24 (m, 2H), 1.61 – 1.50 (m, 2H), 1.43 (d, *J* = 6.6 Hz, 3H), 1.40 – 1.31 (m, 2H), 0.89 (t, *J* = 7.4 Hz, 3H). ^{13}C NMR (101 MHz, $CDCl_3$) δ 167.0, 149.7, 129.7, 129.1, 126.0, 77.5, 68.7, 52.0, 32.0, 24.1, 19.3, 13.9. HRMS (ESI-TOF) (m/z): Calcd for $C_{14}H_{21}O_3$ ($[M + H]^+$): 237.1485; found: 237.1485.

methyl 4-(bicyclo[2.2.1]heptan-2-yl)benzoate (3aq)

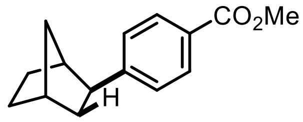

GPII, 40.6 mg, 88% yield, d.r. > 20: 1. ^{1}H NMR (400 MHz, $CDCl_3$) δ 7.94 (d, *J* = 8.4 Hz, 2H), 7.27 (d, *J* = 8.2 Hz, 2H), 3.89 (s, 3H), 2.81 – 2.75 (m, 1H), 2.41 – 2.33 (m, 2H), 1.84 – 1.76 (m, 1H), 1.67 – 1.56 (m, 3H), 1.54 – 1.48 (m, 1H), 1.40 – 1.33 (m, 1H), 1.31 – 1.24 (m, 1H), 1.23 – 1.18 (m, 1H). ^{13}C NMR (101 MHz, $CDCl_3$) δ 167.1, 153.1, 129.5, 127.2, 127.0, 51.9, 47.4, 42.7, 39.1, 36.8, 36.1, 30.5, 28.8. HRMS (ESI-TOF) (m/z): Calcd for $C_{15}H_{19}O_2$ ($[M + H]^+$): 231.1380; found: 231.1380.

***tert*-butyl 3-(4-(methoxycarbonyl)phenyl)-3-methylazetidine-1-carboxylate (3ar)**

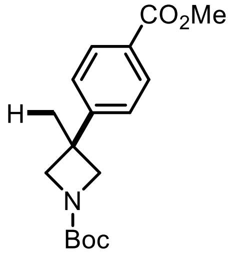

GPI, 52.2 mg, 85% yield. ^{1}H NMR (400 MHz, $CDCl_3$) δ 8.04 – 7.99 (m, 2H), 7.29 – 7.24 (m, 2H), 4.20 (d, *J* = 8.1 Hz, 2H), 3.95 (d, *J* = 8.1 Hz, 2H), 3.92 (s, 3H), 1.64 (s, 3H), 1.45 (s, 9H). ^{13}C NMR (101 MHz, $CDCl_3$) δ 166.7, 156.5, 152.0, 129.9, 128.3, 125.3, 79.6, 52.1, 38.1, 29.0, 28.4. HRMS (ESI-TOF) (m/z): Calcd for $C_{17}H_{23}NO_4Na$ ($[M + Na]^+$): 328.1519; found: 328.1519.

methyl 3-(4-(benzoyloxy)butan-2-yl)benzoate (3bg)

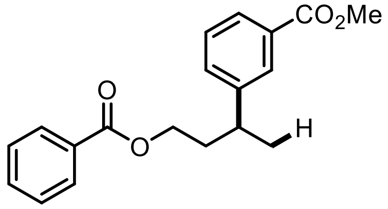

GPII, 41.3 mg, 66% yield. ^{1}H NMR (400 MHz, $CDCl_3$) δ 7.99 – 7.95 (m, 2H), 7.94 – 7.91 (m, 1H), 7.89 – 7.85 (m, 1H), 7.58 – 7.52 (m, 1H), 7.45 – 7.40 (m, 3H), 7.39 – 7.34 (m, 1H), 4.31 – 4.24 (m, 1H), 4.22 – 4.15 (m, 1H), 3.90 (s, 3H), 3.07 – 2.96 (m, 1H), 2.09 (q, *J* = 6.8 Hz, 2H), 1.35 (d, *J* = 7.0 Hz, 3H)· ^{13}C NMR (101 MHz, $CDCl_3$) δ 167.1, 166.4, 146.6, 132.8, 131.7, 130.4, 130.2, 129.5, 128.6, 128.3, 128.0, 127.5, 63.2, 52.1, 36.8, 36.7, 22.3. HRMS (ESI-TOF) (m/z): Calcd for $C_{19}H_{21}O_4$ ([M + H]$^+$): 313.1434; found: 313.1433.

3-(3-oxo-1,3-dihydroisobenzofuran-5-yl)butyl benzoate (3cg)

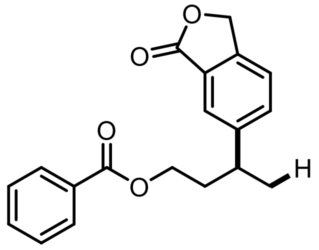

GPII, 46.1 mg, 74% yield. ^{1}H NMR (400 MHz, $CDCl_3$) δ 7.92 – 7.87 (m, 2H), 7.84 (d, *J* = 8.0 Hz, 1H), 7.58 – 7.52 (m, 1H), 7.44 – 7.38 (m, 3H), 7.32 (s, 1H), 5.22 (s, 2H), 4.35 – 4.28 (m, 1H), 4.26 – 4.19 (m, 1H), 3.15 – 3.04 (m, 1H), 2.13 (q, *J* = 6.7 Hz, 2H), 1.38 (d, *J* = 7.0 Hz, 3H). ^{13}C NMR (101 MHz, $CDCl_3$) δ 170.9, 166.3, 153.7, 147.3, 133.0, 129.9, 129.3, 128.3, 128.2, 125.9, 123.9, 120.4, 69.4, 63.1, 37.7, 36.7, 22.4. HRMS (ESI-TOF) (m/z): Calcd for $C_{19}H_{19}O_4$ ([M + H]$^+$): 311.1278; found: 311.1277.

methyl 4-(4-(benzoyloxy)butan-2-yl)-1-naphthoate (3dg)

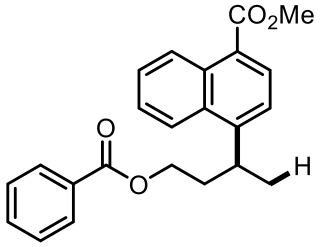

GPII, 54.6 mg, 75% yield. ^{1}H NMR (400 MHz, $CDCl_3$) δ 8.99 – 8.94 (m, 1H), 8.22 (d, *J* = 8.6 Hz, 1H), 8.15 (d, *J* = 7.7 Hz, 1H), 7.99 – 7.94 (m, 2H), 7.62 – 7.50 (m, 3H), 7.47 (d, *J* = 7.7 Hz, 1H), 7.42 (t, *J* = 7.7 Hz, 2H), 4.45 – 4.38 (m, 1H), 4.32 – 4.25 (m, 1H), 3.98 (s, 3H), 3.95 – 3.86 (m, 1H), 2.36 – 2.27 (m, 1H), 2.21 – 2.11 (m, 1H), 1.48 (d, *J* = 6.9 Hz, 3H). ^{13}C NMR (101 MHz, $CDCl_3$) δ 168.0, 166.4, 148.1, 132.9, 131.7, 131.7, 130.1, 129.5, 129.3, 128.3, 127.1, 126.5, 126.2, 125.5, 123.3, 121.5, 63.2, 52.2, 52.0, 36.4, 21.5. HRMS (ESI-TOF) (m/z): Calcd for $C_{23}H_{22}O_4Na$ ($[M + Na]^+$): 385.1410; found: 385.1408.

methyl 6-(4-(benzoyloxy)butan-2-yl)-2-naphthoate (3eg)

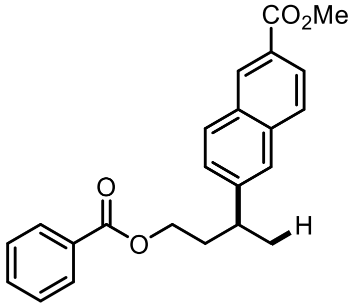

GPII, 51.4 mg, 70% yield. ^{1}H NMR (400 MHz, $CDCl_3$) δ 8.56 (s, 1H), 8.05 – 8.01 (m, 1H), 7.94 – 7.87 (m, 3H), 7.80 (d, *J* = 8.6 Hz, 1H), 7.68 (s, 1H), 7.54 – 7.48 (m, 1H), 7.46 – 7.42 (m, 1H), 7.39 – 7.33 (m, 2H), 4.35 – 4.28 (m, 1H), 4.27 – 4.20 (m, 1H), 3.97 (s, 3H), 3.19 – 3.09 (m, 1H), 2.24 – 2.10 (m, 2H), 1.42 (d, *J* = 7.0 Hz, 3H). ^{13}C NMR (101 MHz, $CDCl_3$) δ 167.3, 166.4, 146.5, 135.7, 132.8, 131.3, 130.7, 130.1, 129.7, 129.4, 128.2, 127.7, 126.8, 126.3, 125.4, 125.1, 63.4, 52.1, 37.3, 36.7, 22.2. HRMS (ESI-TOF) (m/z): Calcd for $C_{23}H_{23}O_4$ ($[M + H]^+$): 363.1591; found: 363.1590.

methyl 3-(4-(benzoyloxy)butan-2-yl)-5-methylbenzoate (3fg)

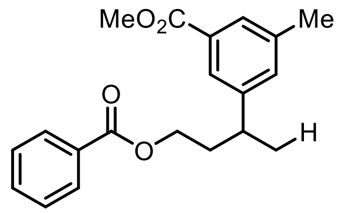

GPII, 48.7 mg, 74% yield. ^{1}H NMR (400 MHz, $CDCl_3$) δ 7.97 (d, J = 7.7 Hz, 2H), 7.70 (d, J = 11.9 Hz, 2H), 7.58 – 7.51 (m, 1H), 7.47 – 7.37 (m, 2H), 7.22 (s, 1H), 4.32 – 4.15 (m, 2H), 3.89 (s, 3H), 3.02 – 2.90 (m, 1H), 2.36 (s, 3H), 2.13 – 2.03 (m, 2H), 1.33 (d, J = 7.1 Hz, 3H). ^{13}C NMR (101 MHz, $CDCl_3$) δ 167.3, 166.4, 146.6, 138.3, 132.8, 132.5, 130.2, 130.2, 129.4, 128.2, 128.1, 125.1, 63.3, 52.0, 36.8, 36.7, 22.3, 21.2. HRMS (ESI-TOF) (m/z): Calcd for $C_{20}H_{23}O_4$ ([M + H]$^+$): 327.1591; found: 327.1590.

methyl 4-(4-(benzoyloxy)butan-2-yl)-2-fluorobenzoate (3gg)

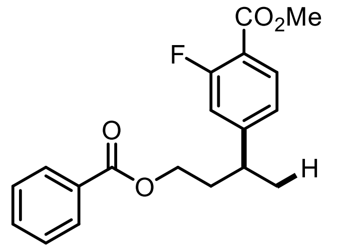

GPII, 39.7 mg, 60% yield. ^{1}H NMR (400 MHz, $CDCl_3$) δ 7.98 – 7.93 (m, 2H), 7.89 – 7.83 (m, 1H), 7.58 – 7.53 (m, 1H), 7.46 – 7.39 (m, 2H), 7.09 – 6.97 (m, 2H), 4.33 – 4.26 (m, 1H), 4.23 – 4.16 (m, 1H), 3.92 (s, 3H), 3.05 – 2.94 (m, 1H), 2.11 – 2.02 (m, 2H), 1.33 (d, *J* = 6.9 Hz, 3H). ^{13}C NMR (101 MHz, Chloroform-*d*) δ 166.4, 164.8 (d, *J* = 4.0 Hz), 162.1 (d, *J* = 260.1 Hz), 154.4 (d, *J* = 8.0 Hz), 132.9, 132.4 (d, *J* = 1.4 Hz), 130.0, 129.4, 128.3, 122.7, 116.4 (d, *J* = 10.0 Hz), 115.2 (d, *J* = 22.6 Hz), 62.9, 52.2, 36.9, 36.5, 21.8. ^{19}F NMR (376 MHz, $CDCl_3$) δ -109.34. HRMS (ESI-TOF) (m/z): Calcd for $C_{19}H_{20}FO_4$ ([M + H]$^+$): 331.1340; found: 331.1340.

methyl 4-(4-(benzoyloxy)butan-2-yl)-2-chlorobenzoate (3hg)

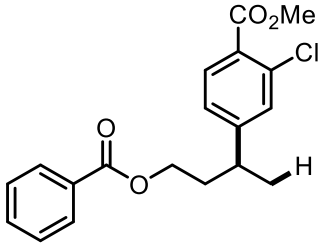

GPII, 43.7 mg, 63% yield. ^{1}H NMR (400 MHz, $CDCl_3$) δ 7.97 – 7.92 (m, 2H), 7.78 (d, J = 8.1 Hz, 1H), 7.58 – 7.52 (m, 1H), 7.46 – 7.40 (m, 2H), 7.32 (d, J = 1.7 Hz, 1H), 7.19 – 7.14 (m, 1H), 4.33 – 4.26 (m, 1H), 4.24 – 4.17 (m, 1H), 3.91 (s, 3H), 3.02 – 2.92 (m, 1H), 2.10 – 2.03 (m, 2H), 1.33 (d, J = 7.0 Hz, 3H). ^{13}C NMR (101 MHz, $CDCl_3$) δ 166.4, 165.9, 152.0, 134.1, 132.9, 131.8, 130.0, 129.6, 129.4, 128.3, 127.6, 125.3, 63.0, 52.3, 36.8, 36.5, 21.9. HRMS (ESI-TOF) (m/z): Calcd for $C_{19}H_{20}ClO_4$ ($[M + H]^+$): 347.1045; found: 347.1045.

methyl 4-(4-(benzoyloxy)butan-2-yl)-2-methylbenzoate (3ig)

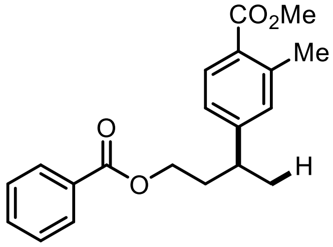

GPII, 50.3 mg, 77% yield. ^{1}H NMR (400 MHz, $CDCl_3$) δ 7.97 – 7.93 (m, 2H), 7.86 (d, *J* = 7.8 Hz, 1H), 7.57 – 7.52 (m, 1H), 7.45 – 7.39 (m, 2H), 7.12 – 7.07 (m, 2H), 4.31 – 4.24 (m, 1H), 4.23 – 4.16 (m, 1H), 3.87 (s, 3H), 2.99 – 2.89 (m, 1H), 2.57 (s, 3H), 2.11 – 2.04 (m, 2H), 1.33 (d, *J* = 7.0 Hz, 3H). ^{13}C NMR (101 MHz, $CDCl_3$) δ 167.8, 166.4, 150.7, 140.6, 132.8, 131.1, 130.4, 130.2, 129.4, 128.3, 127.4, 124.2, 63.3, 51.7, 36.9, 36.6, 22.1, 21.9. HRMS (ESI-TOF) (m/z): Calcd for $C_{20}H_{22}O_4Na$ ($[M + Na]^+$): 349.1410; found: 349.1410.

methyl 4-(4-(benzoyloxy)butan-2-yl)-2-(trifluoromethoxy)benzoate (3jg)

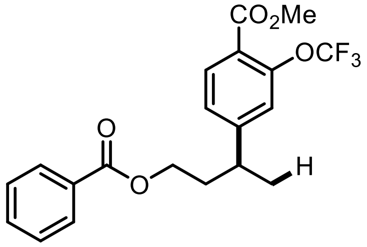

GPII, 54.5 mg, 68% yield. ^{1}H NMR (400 MHz, $CDCl_3$) δ 7.97 – 7.93 (m, 2H), 7.91 – 7.88 (m, 1H), 7.58 – 7.52 (m, 1H), 7.45 – 7.39 (m, 2H), 7.27 – 7.22 (m, 1H), 7.20 – 7.17 (m, 1H), 4.35 – 4.28 (m, 1H), 4.24 – 4.16 (m, 1H), 3.91 (s, 3H), 3.08 – 2.97 (m, 1H), 2.14 – 2.03 (m, 2H), 1.35 (d, J = 6.8 Hz, 3H). ^{13}C NMR (101 MHz, $CDCl_3$) δ 166.3, 164.8, 153.3, 148.0 (q, *J* = 2.0 Hz), 132.9, 132.3, 130.0, 129.4, 128.3, 125.6, 122.7, 121.3 – 121.1 (m), 120.3 (q, J = 257.7 Hz), 62.9, 52.3, 36.9, 36.5, 21.8. ^{19}F NMR (376 MHz, $CDCl_3$) δ -57.35. HRMS (ESI-TOF) (m/z): Calcd for $C_{20}H_{20}F_3O_5$ ($[M + H]^+$): 397.1257; found: 397.1258.

methyl 4-(4-(benzoyloxy)butan-2-yl)-2-methoxybenzoate (3kg)

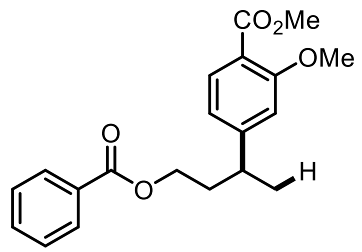

GPII, 60.6 mg, 88% yield. ^{1}H NMR (400 MHz, $CDCl_3$) δ 8.00 – 7.94 (m, 2H), 7.76 (d, *J* = 8.0 Hz, 1H), 7.58 – 7.52 (m, 1H), 7.43 (t, *J* = 7.7 Hz, 2H), 6.88 – 6.84 (m, 1H), 6.82 – 6.79 (m, 1H), 4.32 – 4.25 (m, 1H), 4.24 – 4.17 (m, 1H), 3.87 (s, 6H), 3.02 – 2.91 (m, 1H), 2.13 – 2.01 (m, 2H), 1.34 (d, *J* = 7.0 Hz, 3H). ^{13}C NMR (101 MHz, $CDCl_3$) δ 166.4, 166.4, 159.4, 152.8, 132.9, 132.1, 130.1, 129.4, 128.3, 118.5, 117.8, 110.8, 63.2, 55.9, 51.9, 37.4, 36.6, 22.0. HRMS (ESI-TOF) (m/z): Calcd for $C_{20}H_{23}O_5$ ($[M + H]^+$): 343.1540; found: 343.1540.

3-(3-methoxy-4-(trifluoromethyl)phenyl)butyl benzoate (3lg)

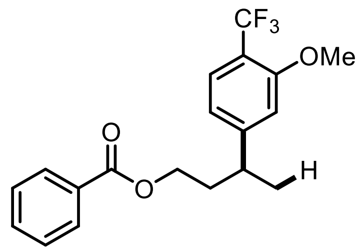

GPII, 51.8 mg, 73% yield. ^{1}H NMR (400 MHz, $CDCl_3$) δ 7.97 – 7.92 (m, 2H), 7.58 – 7.53 (m, 1H), 7.48 (d, *J* = 8.0 Hz, 1H), 7.45 – 7.40 (m, 2H), 6.89 – 6.82 (m, 2H), 4.33 – 4.26 (m, 1H), 4.26 – 4.19 (m, 1H), 3.86 (s, 3H), 3.03 – 2.93 (m, 1H), 2.12 – 2.04 (m,

2H), 1.34 (d, J = 6.9 Hz, 3H). ^{13}C NMR (101 MHz, $CDCl_3$) δ 166.4, 157.6, 152.4, 133.0, 130.1, 129.4, 128.3, 127.7 – 127.0 (m), 123.7 (q, J = 271.9 Hz), 118.3, 116.5, 111.0 – 110.5 (m), 63.2, 55.8, 37.5, 36.6, 22.2. ^{19}F NMR (376 MHz, $CDCl_3$) δ -62.08. HRMS (ESI-TOF) (m/z): Calcd for $C_{19}H_{19}F_3O_3Na$ ($[M + Na]^+$): 375.1179; found: 375.1178.

3-(4-acetylphenyl)butyl benzoate (3mg)

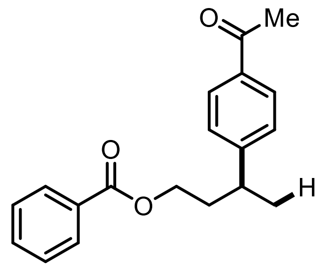

GPII, 43.7 mg, 73% yield. ^{1}H NMR (400 MHz, $CDCl_3$) δ 7.97 – 7.93 (m, 2H), 7.91 – 7.87 (m, 2H), 7.57 – 7.52 (m, 1H), 7.44 – 7.39 (m, 2H), 7.34 – 7.29 (m, 2H), 4.33 – 4.25 (m, 1H), 4.23 – 4.15 (m, 1H), 3.08 – 2.97 (m, 1H), 2.56 (s, 3H), 2.13 – 2.06 (m, 2H), 1.35 (d, J = 7.0 Hz, 3H). ^{13}C NMR (101 MHz, $CDCl_3$) δ 197.7, 166.4, 152.0, 135.4, 132.9, 130.1, 129.4, 128.7, 128.3, 127.1, 63.2, 37.1, 36.6, 26.5, 22.1. HRMS (ESI-TOF) (m/z): Calcd for $C_{19}H_{21}O_3$ ($[M + H]^+$): 297.1485; found: 297.1486.

3-(8-oxo-5,6,7,8-tetrahydronaphthalen-2-yl)butyl benzoate (3ng)

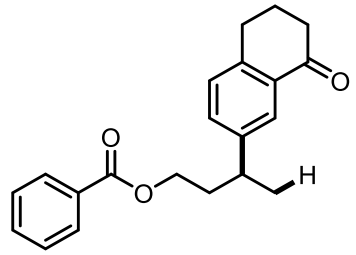

GPII, 36.8 mg, 57% yield. ^{1}H NMR (400 MHz, $CDCl_3$) δ 8.00 – 7.95 (m, 2H), 7.92 (d, J = 2.0 Hz, 1H), 7.58 – 7.52 (m, 1H), 7.45 – 7.40 (m, 2H), 7.35 – 7.31 (m, 1H), 7.18 (d, J = 7.9 Hz, 1H), 4.31 – 4.24 (m, 1H), 4.20 – 4.12 (m, 1H), 3.04 – 2.95 (m, 1H), 2.92 (t, J = 6.1 Hz, 2H), 2.66 – 2.61 (m, 2H), 2.15 – 2.04 (m, 4H), 1.33 (d, J = 7.0 Hz, 3H). ^{13}C NMR (101 MHz, $CDCl_3$) δ 198.6, 166.4, 144.8, 142.5, 132.8, 132.6, 132.4, 130.2, 129.5, 129.0, 128.3, 125.1, 63.3, 39.2, 36.6, 36.6, 29.3, 23.3. HRMS (ESI-TOF) (m/z): Calcd for $C_{21}H_{23}O_3$ ($[M + H]^+$): 323.1642; found: 323.1642.

3-(4-(cyclopropanecarbonyl)phenyl)butyl benzoate (3og)

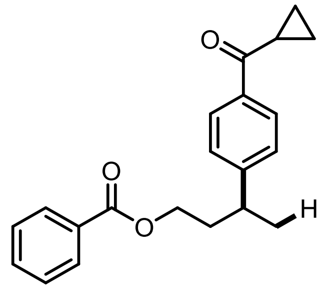

GPII, 41.3 mg, 64% yield. ^{1}H NMR (400 MHz, $CDCl_3$) δ 7.98 – 7.92 (m, 4H), 7.57 – 7.52 (m, 1H), 7.42 (t, *J* = 7.8 Hz, 2H), 7.33 (d, *J* = 8.3 Hz, 2H), 4.33 – 4.26 (m, 1H), 4.24 – 4.18 (m, 1H), 3.08 – 2.97 (m, 1H), 2.67 – 2.60 (m, 1H), 2.14 – 2.06 (m, 2H), 1.36 (d, *J* = 7.0 Hz, 3H), 1.24 – 1.20 (m, 2H), 1.05 – 0.99 (m, 2H). ^{13}C NMR (101 MHz, $CDCl_3$) δ 200.1, 166.4, 151.6, 136.3, 132.9, 130.2, 129.5, 128.4, 128.3, 127.1, 63.2, 37.1, 36.7, 17.0, 11.5. HRMS (ESI-TOF) (m/z): Calcd for $C_{21}H_{23}O_3$ ([M + H]$^+$): 323.1642; found: 323.1642.

3-(2-oxo-2,3-dihydro-1*H*-inden-5-yl)butyl benzoate (3pg)

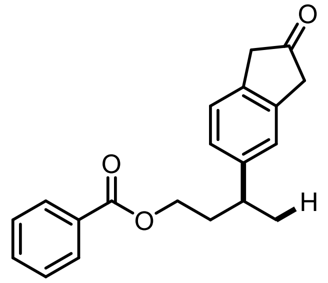

GPII, 50.5 mg, 81% yield. ^{1}H NMR (400 MHz, $CDCl_3$) δ 7.94 – 7.88 (m, 2H), 7.69 (d, *J* = 7.9 Hz, 1H), 7.57 – 7.51 (m, 1H), 7.41 (t, *J* = 7.8 Hz, 2H), 7.31 (s, 1H), 7.27 – 7.23 (m, 1H), 4.34 – 4.27 (m, 1H), 4.26 – 4.18 (m, 1H), 3.10 – 2.98 (m, 3H), 2.68 – 2.58 (m, 2H), 2.16 – 2.06 (m, 2H), 1.36 (d, *J* = 6.9 Hz, 3H). ^{13}C NMR (101 MHz, $CDCl_3$) δ 206.5, 166.4, 155.9, 154.1, 135.6, 132.9, 130.1, 129.4, 128.2, 126.5, 125.0, 123.9, 63.2, 37.7, 36.7, 36.4, 25.7, 22.3. HRMS (ESI-TOF) (m/z): Calcd for $C_{20}H_{21}O_3$ ([M + H]$^+$): 309.1485; found: 309.1485.

3-(4-(piperidin-1-ylsulfonyl)phenyl)butyl benzoate (3qg)

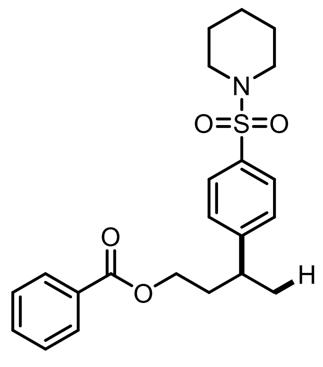

GPII, 67.4 mg, 83% yield. ^{1}H NMR (400 MHz, $CDCl_3$) δ 8.00 – 7.95 (m, 2H), 7.69 (d, J = 8.2 Hz, 2H), 7.60 – 7.54 (m, 1H), 7.47 – 7.42 (m, 2H), 7.38 (d, J = 8.2 Hz, 2H), 4.33 – 4.25 (m, 1H), 4.24 – 4.15 (m, 1H), 3.09 – 3.01 (m, 1H), 2.98 (t, J = 5.5 Hz, 4H), 2.15 – 2.06 (m, 2H), 1.67 – 1.60 (m, 4H), 1.46 – 1.40 (m, 2H), 1.36 (d, J = 7.0 Hz, 3H). ^{13}C NMR (101 MHz, $CDCl_3$) δ 166.4, 151.4, 134.1, 133.0, 130.0, 129.4, 128.3, 128.0, 127.5, 63.0, 46.8, 36.9, 36.6, 25.1, 23.4, 22.0. HRMS (ESI-TOF) (m/z): Calcd for $C_{22}H_{28}NO_4S$ ($[M + H]^+$): 402.1734; found: 402.1733.

3-(2-methyl-1-oxoisoindolin-5-yl)butyl benzoate (3rg)

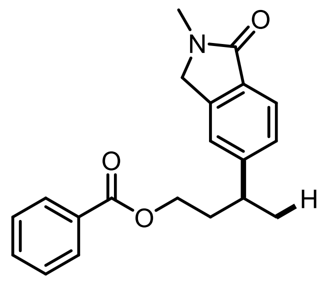

GPII, 51.3 mg, 79% yield. ^{1}H NMR (400 MHz, $CDCl_3$) δ 7.94 – 7.89 (m, 2H), 7.76 (d, J = 7.8 Hz, 1H), 7.57 – 7.51 (m, 1H), 7.40 (t, J = 7.7 Hz, 2H), 7.32 (d, J = 7.8 Hz, 1H), 7.26 (s, 1H), 4.32 – 4.17 (m, 4H), 3.16 (s, 3H), 3.08 – 3.00 (m, 1H), 2.10 (q, J = 6.7 Hz, 2H), 1.36 (d, J = 7.0 Hz, 3H). ^{13}C NMR (101 MHz, $CDCl_3$) δ 168.5, 166.4, 150.2, 141.5, 132.8, 131.1, 130.0, 129.4, 128.2, 126.9, 123.7, 121.0, 63.2, 51.8, 37.4, 36.8, 29.4, 22.4. HRMS (ESI-TOF) (m/z): Calcd for $C_{20}H_{22}NO_3$ ($[M + H]^+$): 324.1594; found: 324.1594.

3-(1,3-dioxoisoindolin-5-yl)butyl benzoate (3sg)

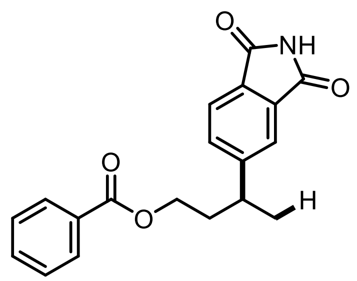

GPII, 37.2 mg, 57% yield. ^{1}H NMR (400 MHz, $CDCl_3$) δ 7.95 – 7.90 (m, 2H), 7.78 – 7.73 (m, 2H), 7.61 – 7.53 (m, 2H), 7.42 (t, *J* = 7.7 Hz, 2H), 4.37 – 4.29 (m, 1H), 4.25 – 4.18 (m, 1H), 3.19 – 3.08 (m, 1H), 2.14 (q, *J* = 6.7 Hz, 2H), 1.68 (s, 1H), 1.39 (d, *J* = 6.9 Hz, 3H). ^{13}C NMR (101 MHz, $CDCl_3$) δ 167.9, 167.6, 166.3, 154.2, 133.3, 133.3, 133.0, 130.7, 129.9, 129.4, 128.3, 123.9, 121.9, 62.9, 37.7, 36.7. HRMS (ESI-TOF) (m/z): Calcd for $C_{19}H_{18}NO_4$ ($[M + H]^+$): 324.1230; found: 324.1231.

3-(naphthalen-1-yl)butyl benzoate (3tg)

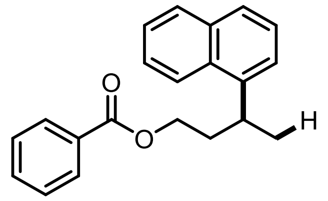

GPII, 49.4 mg, 81% yield. ^{1}H NMR (400 MHz, $CDCl_3$) δ 8.18 – 8.13 (m, 1H), 8.03 – 7.98 (m, 2H), 7.87 – 7.82 (m, 1H), 7.73 – 7.68 (m, 1H), 7.56 – 7.51 (m, 1H), 7.48 – 7.39 (m, 6H), 4.45 – 4.37 (m, 1H), 4.31 – 4.24 (m, 1H), 3.91 – 3.81 (m, 1H), 2.36 – 2.26 (m, 1H), 2.21 – 2.10 (m, 1H), 1.47 (d, *J* = 6.9 Hz, 3H).^{13}C NMR (101 MHz, $CDCl_3$) δ 166.5, 142.4, 133.9, 132.8, 131.4, 130.3, 129.5, 129.0, 128.3, 126.6, 125.8, 125.6, 125.3, 122.9, 122.5, 63.5, 36.6, 30.5, 21.6. HRMS (ESI-TOF) (m/z): Calcd for $C_{21}H_{21}O_2$ ($[M + H]^+$): 305.1536; found: 305.1536.

3-(naphthalen-2-yl)butyl benzoate (3ug)

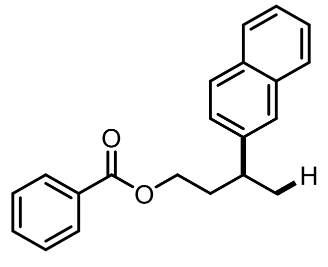

GPII, 39.1 mg, 64% yield. ^{1}H NMR (400 MHz, $CDCl_3$) δ 7.96 – 7.92 (m, 2H), 7.81 – 7.75 (m, 3H), 7.65 (d, *J* = 1.7 Hz, 1H), 7.54 – 7.49 (m, 1H), 7.47 – 7.40 (m, 2H), 7.39

– 7.34 (m, 3H), 4.33 – 4.27 (m, 1H), 4.26 – 4.19 (m, 1H), 3.16 – 3.06 (m, 1H), 2.19 – 2.09 (m, 2H), 1.41 (d, *J* = 7.0 Hz, 3H). ^{13}C NMR (101 MHz, $CDCl_3$) δ 166.5, 143.7, 133.6, 132.8, 132.3, 130.3, 129.4, 128.2, 128.2, 127.5, 127.5, 125.9, 125.4, 125.2, 125.2, 63.5, 37.1, 36.8, 22.4. HRMS (ESI-TOF) (m/z): Calcd for $C_{21}H_{21}O_2$ ([M + H]$^+$): 305.1536; found: 305.1536.

3-(4-ethoxyphenyl)butyl benzoate (3vg)

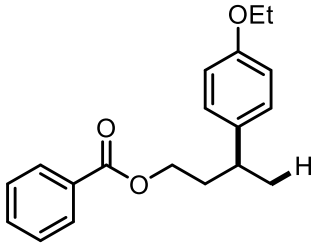

GPII, 21.2 mg, 35% yield. ^{1}H NMR (400 MHz, $CDCl_3$) δ 8.03 – 7.95 (m, 2H), 7.57 – 7.51 (m, 1H), 7.46 – 7.39 (m, 2H), 7.15 – 7.08 (m, 2H), 6.86 – 6.79 (m, 2H), 4.30 – 4.22 (m, 1H), 4.20 – 4.13 (m, 1H), 4.00 (q, *J* = 6.9 Hz, 2H), 2.94 – 2.84 (m, 1H), 2.10 – 1.92 (m, 2H), 1.40 (t, *J* = 6.9 Hz, 3H), 1.30 (d, *J* = 6.9 Hz, 3H). ^{13}C NMR (101 MHz, $CDCl_3$) δ 166.5, 157.3, 138.1, 132.8, 130.4, 129.5, 128.3, 127.8, 114.4, 63.5, 63.3, 37.1, 36.0, 22.5, 14.9. HRMS (ESI-TOF) (m/z): Calcd for $C_{19}H_{23}O_3$ ([M + H]$^+$): 299.1642; found: 299.1642.

***tert*-butyl 5-(4-(benzoyloxy)butan-2-yl)-1*H*-indole-1-carboxylate (3wg)**

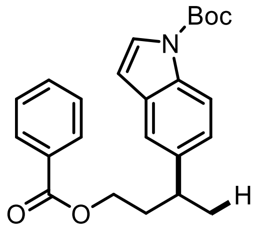

GPII（Ni(dtbbpy)Br_2, 10 mol%）, 34.5 mg, 43% yield. ^{1}H NMR (400 MHz, $CDCl_3$) δ 8.04 (d, *J* = 8.6 Hz, 1H), 7.99 – 7.95 (m, 2H), 7.59 – 7.49 (m, 2H), 7.44 – 7.37 (m, 3H), 7.21 – 7.15 (m, 1H), 6.52 – 6.49 (m, 1H), 4.33 – 4.15 (m, 2H), 3.10 – 2.98 (m, 1H), 2.15 – 2.06 (m, 2H), 1.66 (s, 9H), 1.37 (d, *J* = 7.0 Hz, 3H). ^{13}C NMR (101 MHz, $CDCl_3$) δ 166.5, 149.8, 140.7, 132.7, 130.9, 130.4, 129.5, 128.2, 126.1, 123.3, 118.9, 115.2, 107.2, 83.5, 63.6, 37.3, 36.9, 28.2. HRMS (ESI-TOF) (m/z): Calcd for $C_{24}H_{27}NO_4K$ ([M + K]$^+$): 432.1572; found: 432.1572.

***tert*-butyl 3-(4-((((3*aR*,4*R*,6*aR*)-2,2-dimethyl-6-oxotetrahydrofuro[3,4-*d*][1,3]dioxol-4-yl)methoxy)carbonyl)phenyl)-3-methylazetidine-1-carboxylate (3xr)**

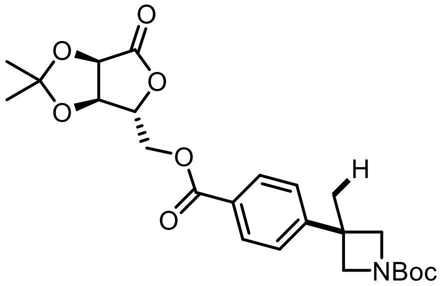

GPII, 70.7 mg, 76% yield. ^{1}H NMR (400 MHz, $CDCl_3$) δ 7.93 – 7.88 (m, 2H), 7.33 – 7.27 (m, 2H), 4.92 – 4.88 (m, 1H), 4.84 – 4.76 (m, 2H), 4.68 – 4.61 (m, 1H), 4.56 – 4.49 (m, 1H), 4.19 (d, *J* = 8.1 Hz, 2H), 3.96 (d, *J* = 7.8 Hz, 2H), 1.64 (s, 3H), 1.51 (s, 3H), 1.46 (s, 9H), 1.39 (s, 3H). ^{13}C NMR (101 MHz, $CDCl_3$) δ 173.4, 165.2, 156.4, 153.0, 129.9, 126.8, 125.7, 113.8, 79.8, 79.7, 77.8, 75.2, 63.7, 38.1, 28.9, 28.3, 26.7, 25.5. HRMS (ESI-TOF) (m/z): Calcd for $C_{24}H_{31}NO_8Na$ ($[M + Na]^+$): 484.1942; found: 484.1944.

***tert*-butyl 3-(4-((((3*aR*,5*R*,6*S*,6*aR*)-5-((*R*)-2,2-dimethyl-1,3-dioxolan-4-yl)-2,2-dimethyltetrahydrofuro[2,3-*d*][1,3]dioxol-6-yl)oxy)carbonyl)phenyl)-3-methylazetidine-1-carboxylate (3yr)**

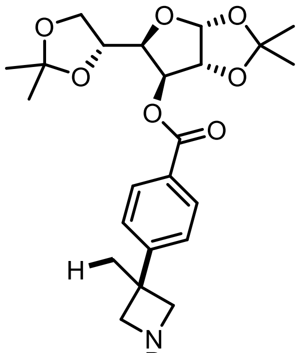

GPII, 83.2 mg, 77% yield. ^{1}H NMR (400 MHz, $CDCl_3$) δ 8.02 (d, *J* = 8.1 Hz, 2H), 7.32 (d, *J* = 8.1 Hz, 2H), 5.88 (d, *J* = 4.1 Hz, 1H), 5.32 (t, *J* = 6.0 Hz, 1H), 4.93 (t, *J* = 5.0 Hz, 1H), 4.76 (q, *J* = 7.2 Hz, 1H), 4.23 – 4.12 (m, 4H), 3.97 (d, *J* = 8.1 Hz, 2H), 3.63 (t, *J* = 7.8 Hz, 1H), 1.65 (s, 3H), 1.54 (s, 3H), 1.48 – 1.44 (m, 12H), 1.42 (s, 3H),

1.34 (s, 3H). ^{13}C NMR (101 MHz, $CDCl_3$) δ 165.1, 156.4, 152.8, 130.1, 127.1, 125.6, 114.8, 109.5, 105.1, 81.2, 79.7, 79.1, 75.2, 72.2, 66.3, 38.1, 28.3, 27.0, 26.6, 25.3. HRMS (ESI-TOF) (m/z): Calcd for $C_{28}H_{39}NO_9K$ ($[M + K]^+$): 572.2256; found: 572.2257.

***tert*-butyl 3-methyl-3-(4-((((3a*S*,5a*R*,8a*R*,8b*S*)-2,2,7,7-tetramethyltetrahydro-3a*H*-bis([1,3]dioxolo)[4,5-*b*:4',5'-*d*]pyran-3a-yl)methoxy)carbonyl)phenyl)azetidine-1-carboxylate (3zr)**

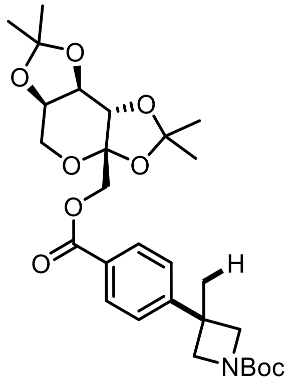

GPII, 80.3 mg, 75% yield. ^1H NMR (400 MHz, $CDCl_3$) δ 8.05 (d, J = 8.1 Hz, 2H), 7.29 – 7.25 (m, 2H), 4.70 – 4.62 (m, 2H), 4.46 (d, J = 2.6 Hz, 1H), 4.34 (d, J = 11.8 Hz, 1H), 4.27 (d, J = 7.9 Hz, 1H), 4.20 (d, J = 8.1 Hz, 2H), 3.99 – 3.92 (m, 3H), 3.81 (d, J = 13.0 Hz, 1H), 1.64 (s, 3H), 1.56 (s, 3H), 1.47 (s, 3H), 1.45 (s, 9H), 1.38 (s, 3H), 1.35 (s, 3H). ^{13}C NMR (101 MHz, $CDCl_3$) δ 165.6, 152.2, 130.1, 128.0, 125.3, 109.1, 108.8, 101.6, 79.7, 70.8, 70.5, 70.1, 65.2, 61.3, 38.1, 28.4, 26.5, 25.9, 25.5, 24.0. HRMS (ESI-TOF) (m/z): Calcd for $C_{28}H_{39}NO_9Na$ ($[M + Na]^+$): 556.2517; found: 556.2517.

methyl 4-((4-methyltetrahydrofuran-3-yl)methyl)benzoate (5)

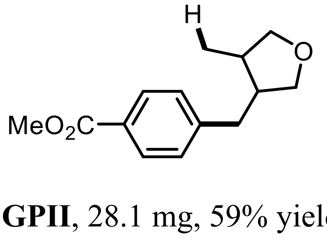

GPII, 28.1 mg, 59% yield. ^1H NMR (400 MHz, $CDCl_3$) δ 7.98 – 7.95 (m, 2H), 7.27 – 7.24 (m, 2H), 3.98 – 3.93 (m, 1H), 3.91 (s, 3H), 3.79 – 3.74 (m, 1H), 3.55 – 3.48 (m, 2H), 2.87 – 2.79 (m, 1H), 2.62 – 2.49 (m, 2H), 2.41 – 2.31 (m, 1H), 1.04 (d, J = 7.0

Hz, 3H). ^{13}C NMR (101 MHz, $CDCl_3$) δ 167.0, 146.4, 129.8, 128.6, 128.0, 75.0, 71.5, 52.0, 43.6, 35.9, 33.6, 13.1. HRMS (ESI-TOF) (m/z): Calcd for $C_{14}H_{19}O_3$ ([M + H]$^+$): 235.1329; found: 235.1329.

diethyl 3-(4-(methoxycarbonyl)benzyl)-4-methylcyclopentane-1,1-dicarboxylate (7)

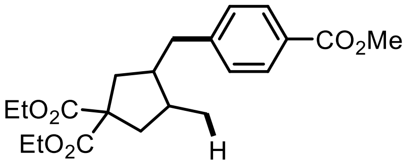

GPII, 64.1 mg, 85% yield. ^{1}H NMR (400 MHz, $CDCl_3$) δ 7.96 – 7.92 (m, 2H), 7.28 – 7.24 (m, 2H), 4.22 – 4.16 (m, 2H), 4.16 – 4.11 (m, 2H), 3.90 (s, 3H), 2.79 – 2.71 (m, 1H), 2.54 – 2.43 (m, 2H), 2.37 – 2.28 (m, 1H), 2.24 – 2.15 (m, 2H), 2.09 – 2.00 (m, 2H), 1.24 (t, J = 7.1 Hz, 3H), 1.20 (t, J = 7.1 Hz, 3H), 0.96 (d, J = 7.1 Hz, 3H). ^{13}C NMR (101 MHz, $CDCl_3$) δ 172.8, 167.1, 146.9, 129.6, 128.8, 127.8, 61.3, 58.7, 51.9, 44.1, 41.2, 37.7, 36.1, 35.7, 15.0, 13.9. HRMS (ESI-TOF) (m/z): Calcd for $C_{21}H_{29}O_6$ ([M + H]$^+$): 377.1959; found: 377.1958.

5. Mechanism study experiments

5.1 Influence of radical scavenger

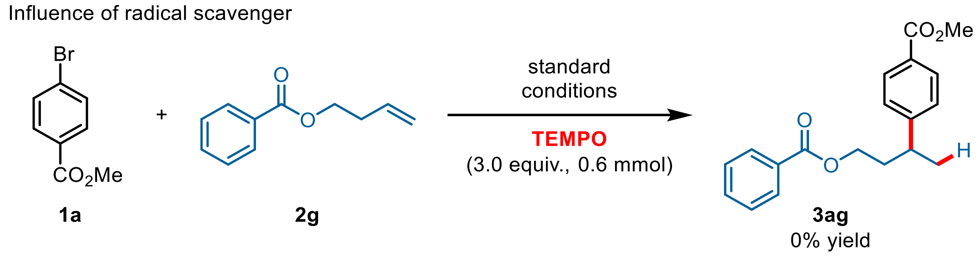

In an oven-dried 10 mL tube equipped with a stirring bar, aryl bromide compounds (0.2 mmol, 1.0 equiv.), unactivated olefins (0.5 mmol, 2.5 equiv.), Hantzsch ester (0.3 mmol, 1.5 equiv.), 4CzIPN (0.004 mmol, 2 mol%), Ni(dtbbpy)Br_2 (0.01 mmol, 5 mol%), Co(TPP) (0.002 mmol, 1 mol%) and TEMPO (0.6 mmol, 3.0 equiv.) were added. The tube was charged with nitrogen (repeated three times), then DMAc (2 mL) was injected. The resulting suspension was stirred at room temperature and irradiated with blue LEDs (440 nm, 10W) by cooling with a fan for 15 h. After completion, the resulting mixture was monitored by TLC, and no desired product (**3ag**) was formed.

5.2 Procedure of ring closure experiments

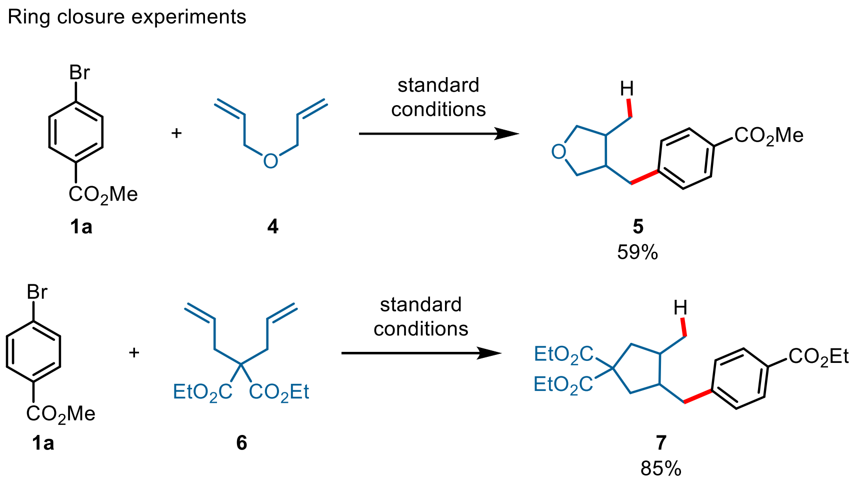

In an oven-dried 10 mL tube equipped with a stirring bar, **1a** (0.2 mmol, 1.0 equiv), Olefins **4** or **6** (0.5 mmol, 2.5 equiv), Hantzsch ester (0.3 mmol, 1.5 equiv.), 4CzIPN (0.004 mmol, 2 mol%), Ni(dtbbpy)Br_2 (0.01 mmol, 5 mol%), and Co(TPP) (0.002 mmol, 1 mol%) were added. The tube was charged with nitrogen (repeated three times), then DMAc (2 mL) was injected. The resulting suspension was stirred at room temperature and irradiated with blue LEDs (440 nm, 10 W) by cooling with a fan for 15 h. After completion, the resulting mixture was quenched with H_2O (30 mL). Then the mixture was extracted with ethyl acetate (30 mL). The organic layer was washed with brine (30 mL × 2), dried over anhydrous Na_2SO_4, and concentrated in vacuum. The residue was purified by flash chromatography on silica gel to afford the desired products.

5.3 Deuterium-labelling experiments

5.3.1 HE-d_2 was employed

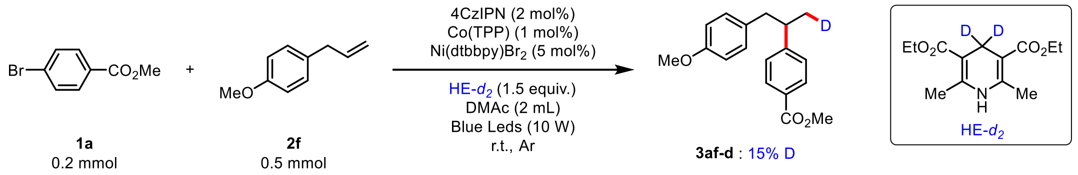

In an oven-dried 10 mL tube equipped with a stirring bar, **1a** (0.2 mmol, 1.0 equiv), HE-d_2 (0.3 mmol, 1.5 equiv.), 4CzIPN (0.004 mmol, 2 mol%), Ni(dtbbpy)Br_2 (0.01 mmol, 5 mol%), and Co(TPP) (0.002 mmol, 1 mol%) were added. The tube was charged with nitrogen (repeated three times), then DMAc (2 mL) and olefin **2f** (0.5

mmol, 2.5 equiv.) were injected. The resulting suspension was stirred at room temperature and irradiated with blue LEDs (440 nm, 10 W) by cooling with a fan for 6 h. After completion, the resulting mixture was quenched with H_2O (30 mL). Then the mixture was extracted with ethyl acetate (30 mL). The organic layer was washed with brine (30 mL × 2), dried over anhydrous Na_2SO_4, and concentrated in vacuum. The residue was purified by flash chromatography on silica gel to afford the desired products (40.3 mg, 70% yield).

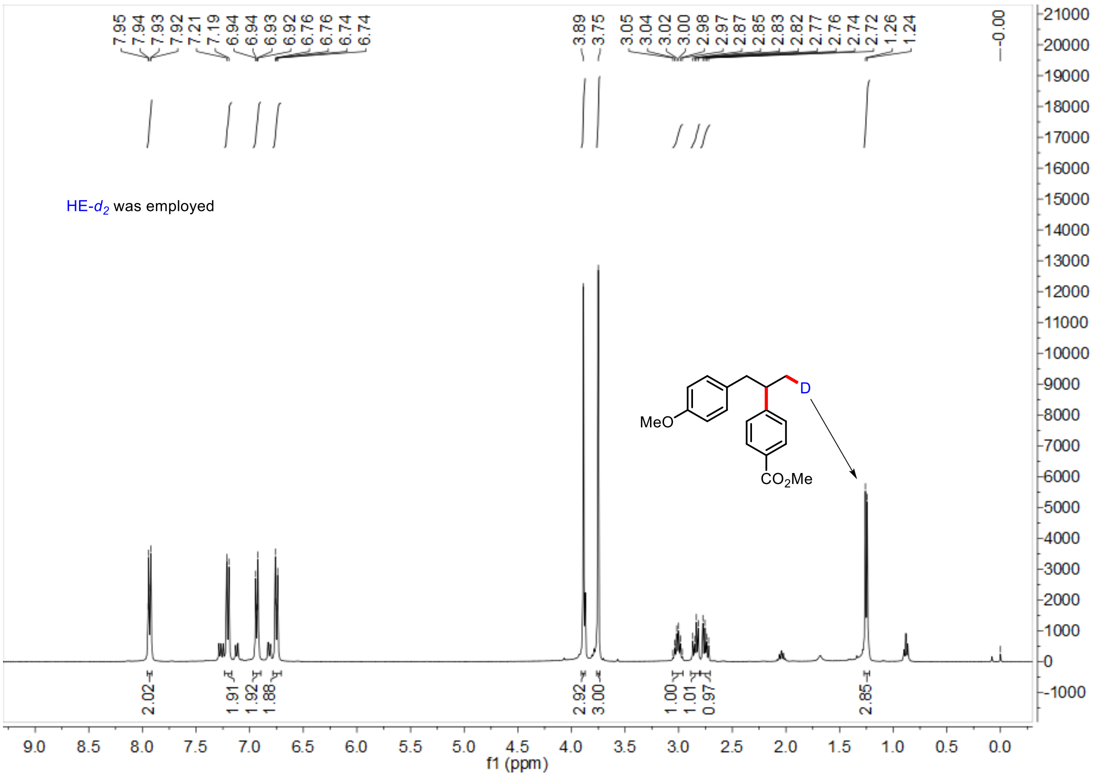

5.3.2 HE-*d*$_3$ was employed

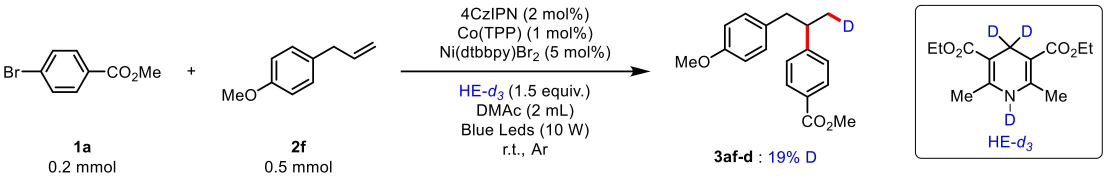

In an oven-dried 10 mL tube equipped with a stirring bar, **1a** (0.2 mmol, 1.0 equiv), HE-*d*$_3$ (0.3 mmol, 1.5 equiv.), 4CzIPN (0.004 mmol, 2 mol%), Ni(dtbbpy)Br_2 (0.01 mmol, 5 mol%), and Co(TPP) (0.002 mmol, 1 mol%) were added. The tube was charged with nitrogen (repeated three times), then DMAc (2 mL) and olefin **2f** (0.5 mmol, 2.5 equiv.) were injected. The resulting suspension was stirred at room temperature and irradiated with blue LEDs (440 nm, 10 W) by cooling with a fan for

6 h. After completion, the resulting mixture was quenched with H_2O (30 mL). Then the mixture was extracted with ethyl acetate (30 mL). The organic layer was washed with brine (30 mL × 2), dried over anhydrous Na_2SO_4, and concentrated in vacuum. The residue was purified by flash chromatography on silica gel to afford the desired products (41.5 mg, 72% yield).

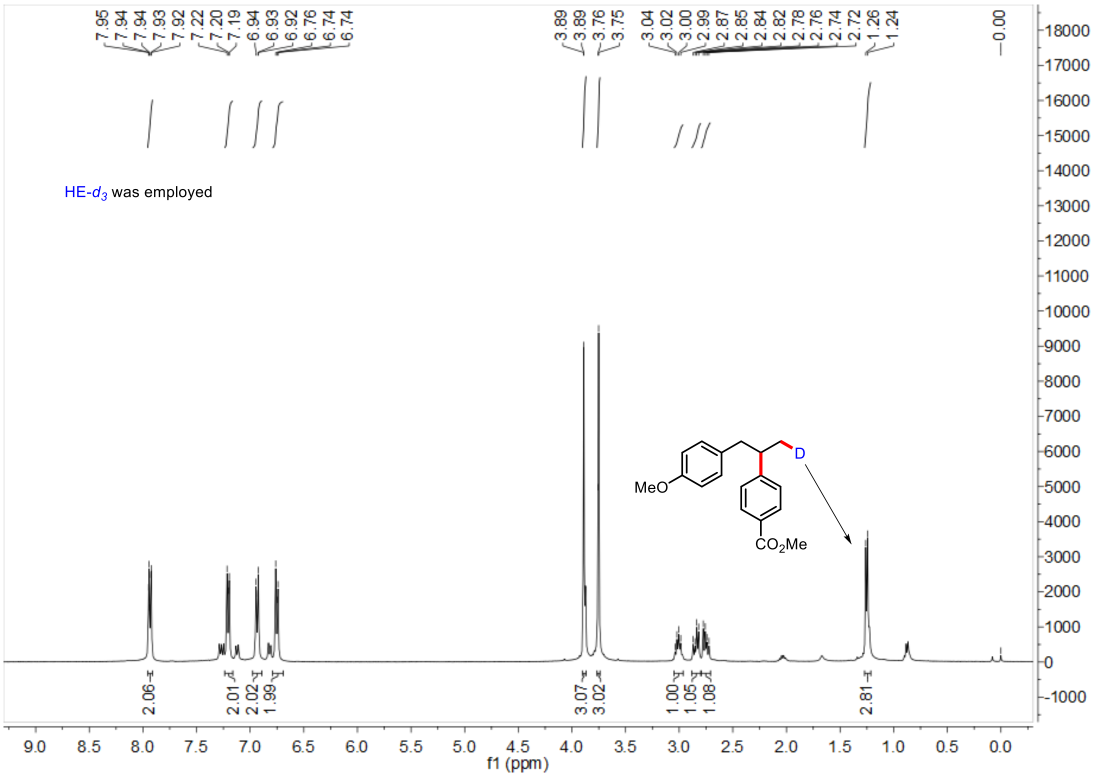

5.3.3 D_2O was employed

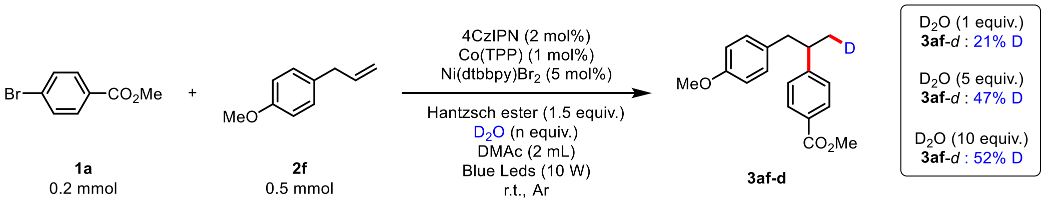

In an oven-dried 10 mL tube equipped with a stirring bar, **1a** (0.2 mmol, 1.0 equiv), Hantzsch ester (0.3 mmol, 1.5 equiv.), 4CzIPN (0.004 mmol, 2 mol%), Ni(dtbbpy)Br_2 (0.01 mmol, 5 mol%) and Co(TPP) (0.002 mmol, 1 mol%) were added. The tube was charged with nitrogen (repeated three times), then DMAc (2 mL), olefin **2f** (0.5 mmol, 2.5 equiv.) and D_2O (n equiv.) were injected. The resulting suspension was stirred at room temperature and irradiated with blue LEDs (440 nm, 10 W) by cooling with a fan for 15 h. After completion, the resulting mixture was quenched with H_2O (30 mL). Then the mixture was extracted with ethyl acetate (30 mL). The organic layer was

washed with brine (30 mL × 2), dried over anhydrous Na_2SO_4, and concentrated in vacuum. The residue was purified by flash chromatography on silica gel to afford the desired products.

Entry	n	D	Yield
1	1 equiv.	21% D	42.5 mg/74%
2	5 equiv.	47% D	41.7 mg/73%
3	10 equiv.	52% D	42.5 mg/74%

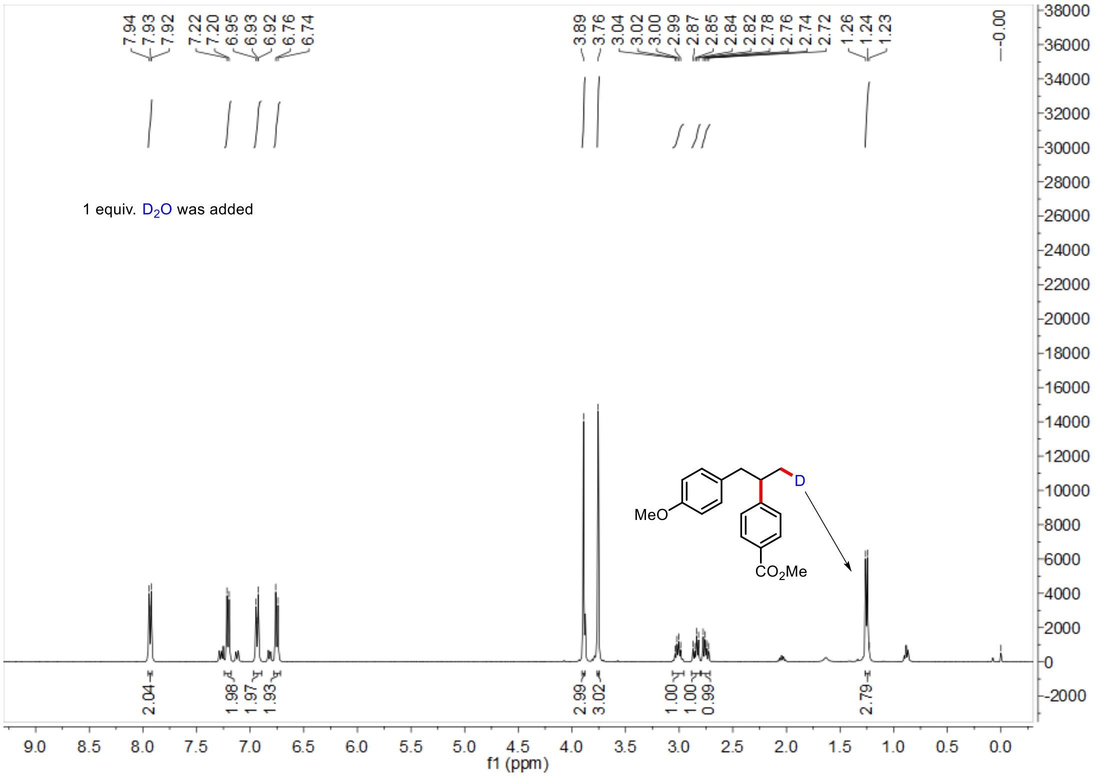

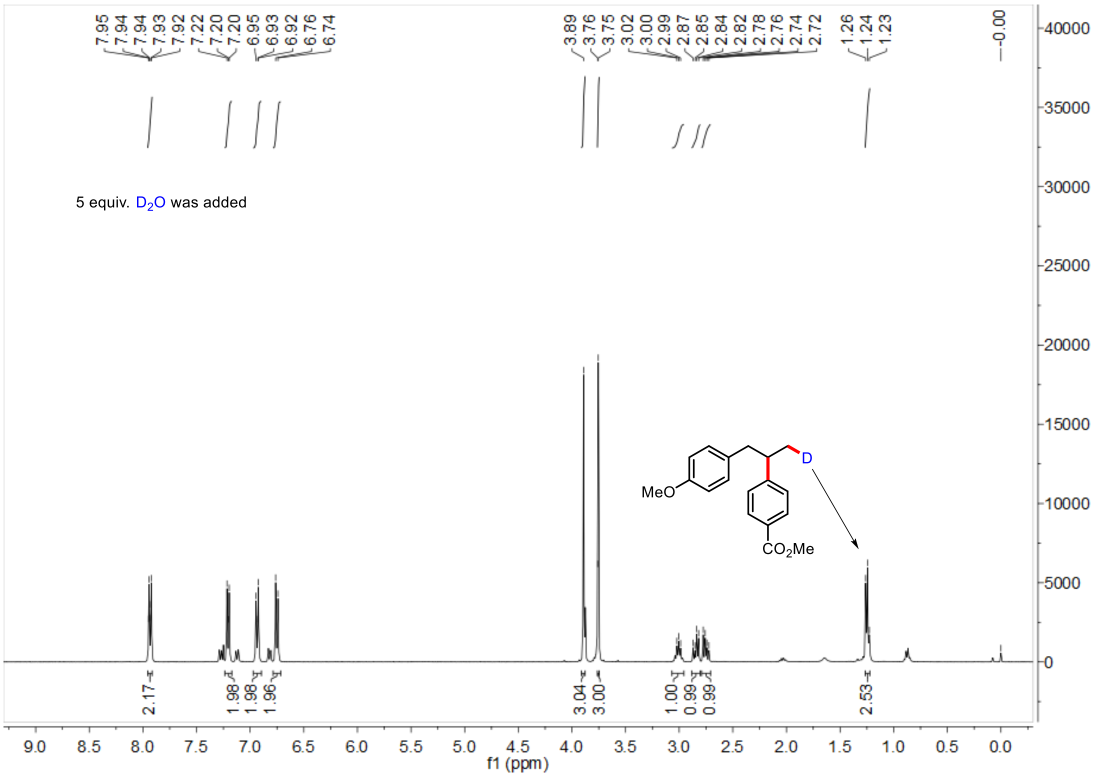
5 equiv. D_2O was added
MeO
D
CO_2Me
f1 (ppm)

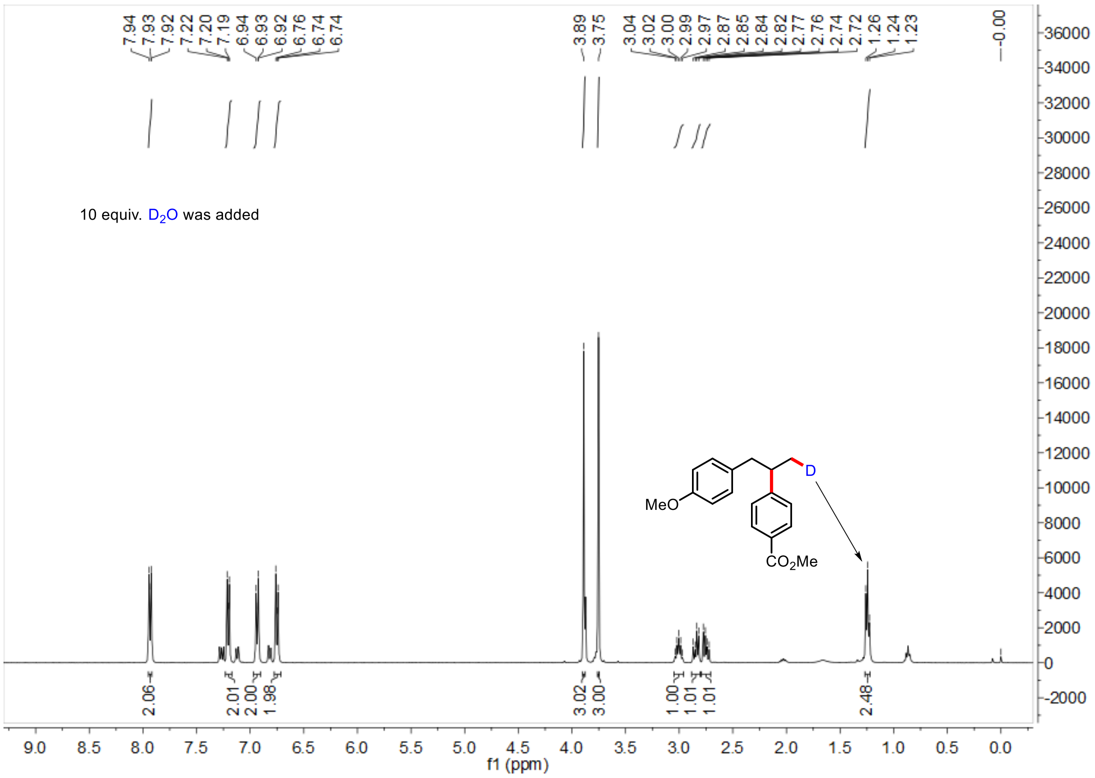
10 equiv. D_2O was added
MeO
D
CO_2Me
f1 (ppm)

5.3.4 DMF-d_7 was employed as solvent

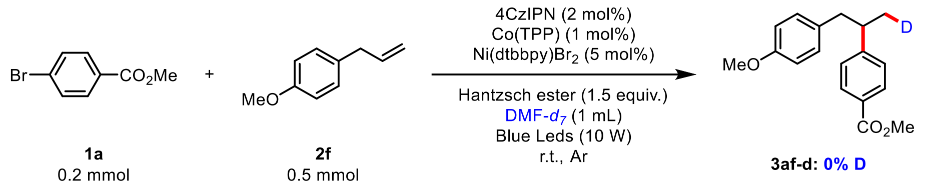

In an oven-dried 10 mL tube equipped with a stirring bar, **1a** (0.2 mmol, 1.0 equiv), Hantzsch ester (0.3 mmol, 1.5 equiv.), 4CzIPN (0.004 mmol, 2 mol%), Ni(dtbbpy)Br_2 (0.01 mmol, 5 mol%) and Co(TPP) (0.002 mmol, 1 mol%) were added. The tube was charged with nitrogen (repeated three times), then DMF-d_7 (1 mL, here we used DMF-d_7 instead of DMAc-d_9) and olefin **2f** (0.5 mmol, 2.5 equiv.) were injected. The resulting suspension was stirred at room temperature and irradiated with blue LEDs (440 nm, 10 W) by cooling with a fan for 15 h. After completion, the resulting mixture was quenched with H_2O (30 mL). Then the mixture was extracted with ethyl acetate (30 mL). The organic layer was washed with brine (30 mL × 2), dried over anhydrous Na_2SO_4, and concentrated in vacuum. The residue was purified by flash chromatography on silica gel to afford the desired products (32.8 mg, 57% yield).

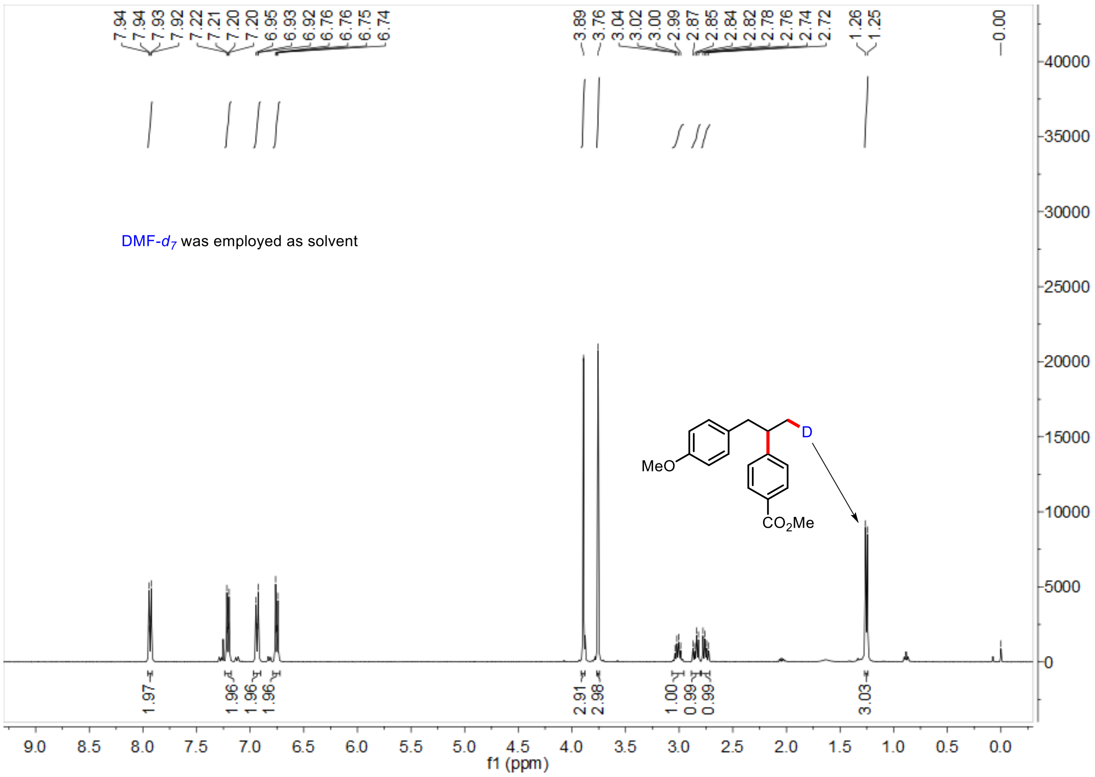

5.4 UV-Vis absorption experiments

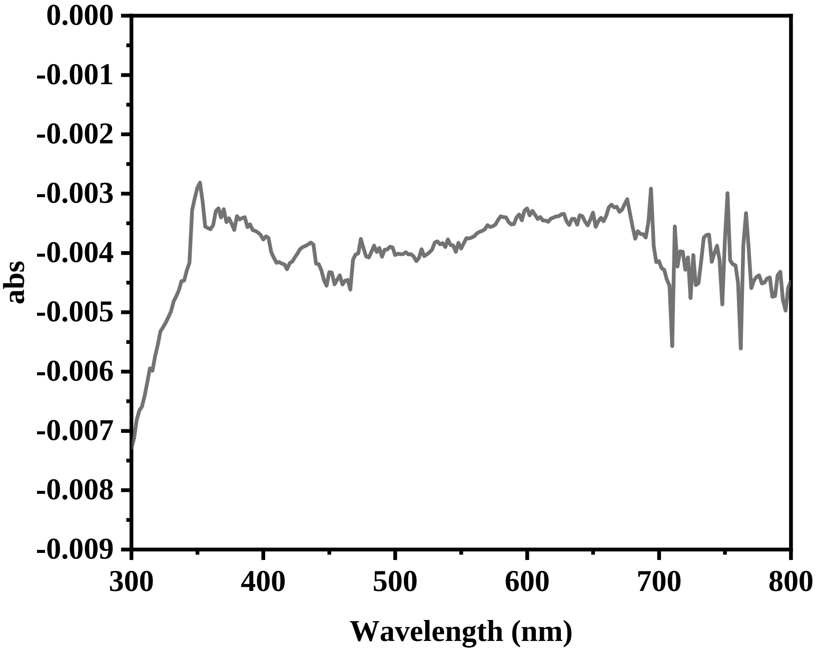

Figure S1 UV-Vis Absorption of **1a** in DMAc (Concentration: 0.1 mM)

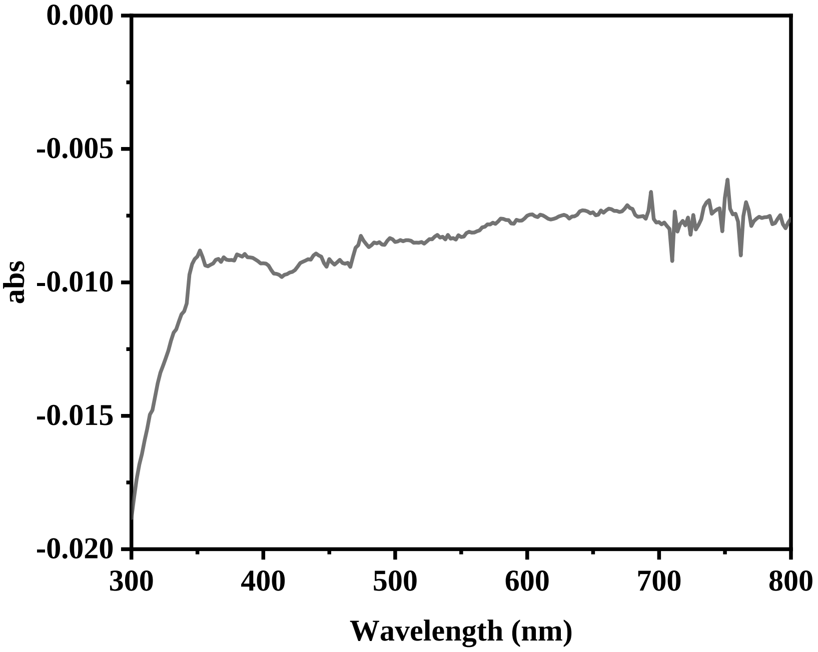

Figure S2 UV-Vis Absorption of **2a** in DMAc (Concentration: 0.1 mM)

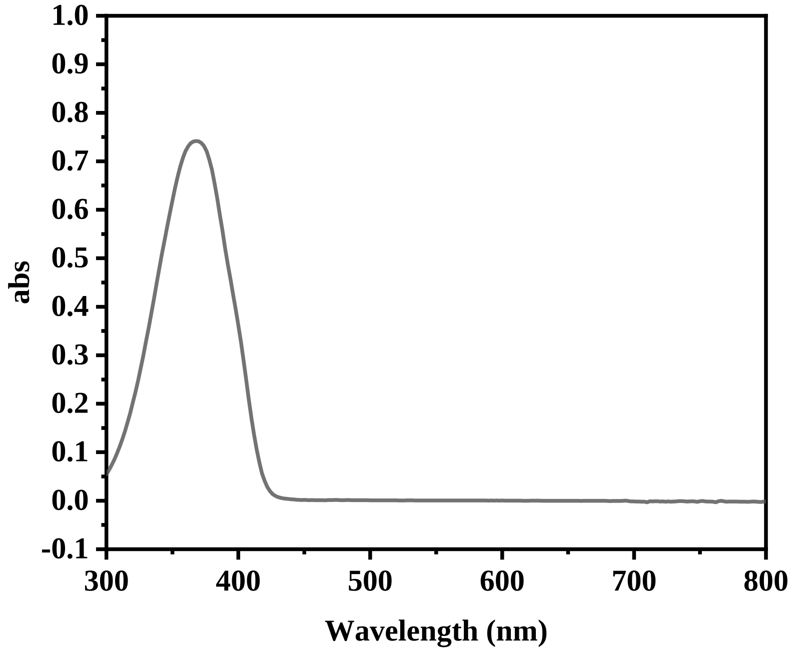

Figure S3 UV-Vis Absorption of Hantzsch ester in DMAc (Concentration: 0.1 mM)

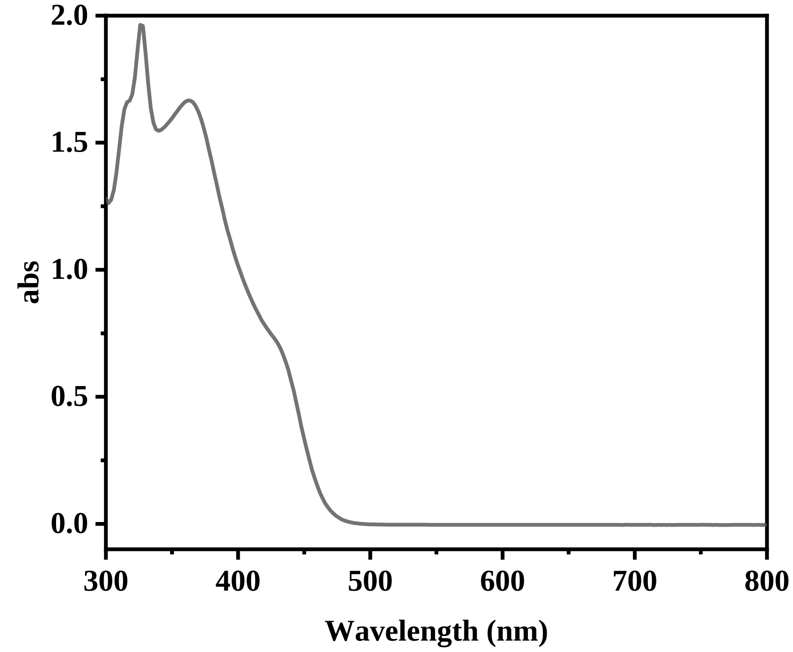

Figure S4 UV-Vis Absorption of 4CzIPN in DMAc (Concentration: 0.1 mM)

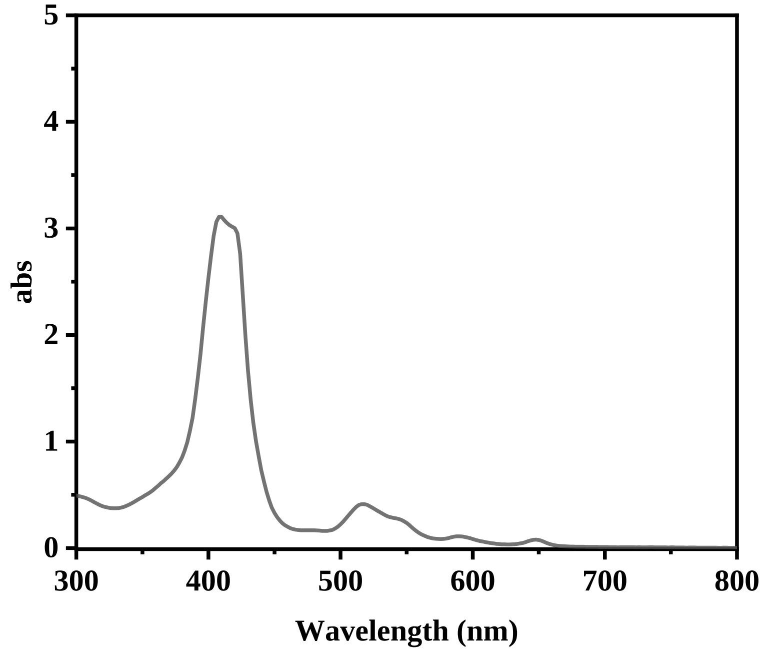

Figure S5 UV-Vis Absorption of Co(TPP) in DMAc (Concentration: 0.1 mM)

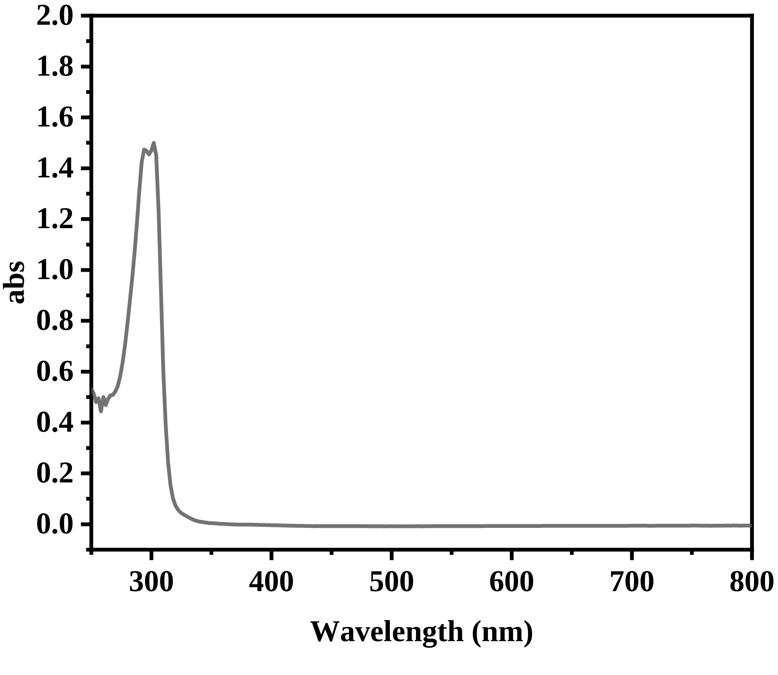

Figure S6 UV-Vis Absorption of Ni(dtbbpy)Br_2 in DMAc (Concentration: 0.1 mM)

5.5 Stern-Volmer fluorescence quenching experiments

5.5.1 Quenching of 4CzIPN with Hantzsch ester

Excitation wavelength: 440 nm.

Excitation slit: 5 nm; emission slit: 5 nm.

To a standard solution of 4CzIPN in DMAc (0.1 mM) were added different amounts of a solution of Hantzsch ester in DMAc to afford the final concentrations reported in the figure below. The fluorescence of the solutions was then measured. DMAc was degassed with a stream of nitrogen for 30 min.

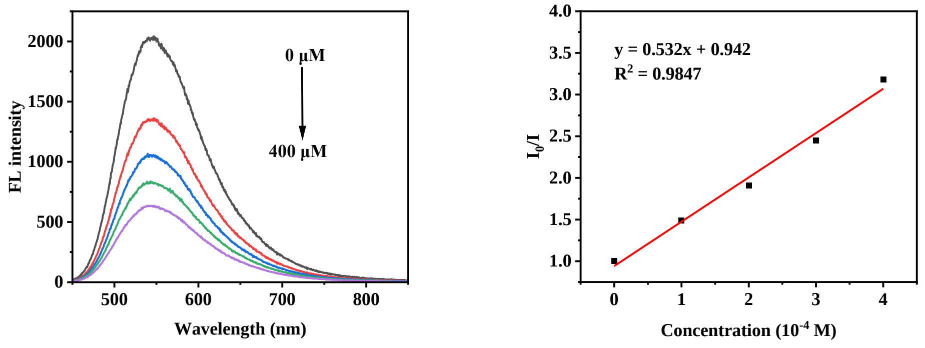

Figure S7 Emission spectra of the 4CzIPN with increasing concentrations of Hantzsch ester

5.5.2 Quenching of 4CzIPN with Ni(dtbbpy)Br_2

Excitation wavelength: 440 nm.

Excitation slit: 5 nm; emission slit: 5 nm.

To a standard solution of 4CzIPN in DMAc (0.1 mM) were added different amounts of a solution of Ni(dtbbpy)Br_2 in DMAc to afford the final concentrations reported in the figure below. The fluorescence of the solutions was then measured. DMAc was degassed with a stream of nitrogen for 30 min.

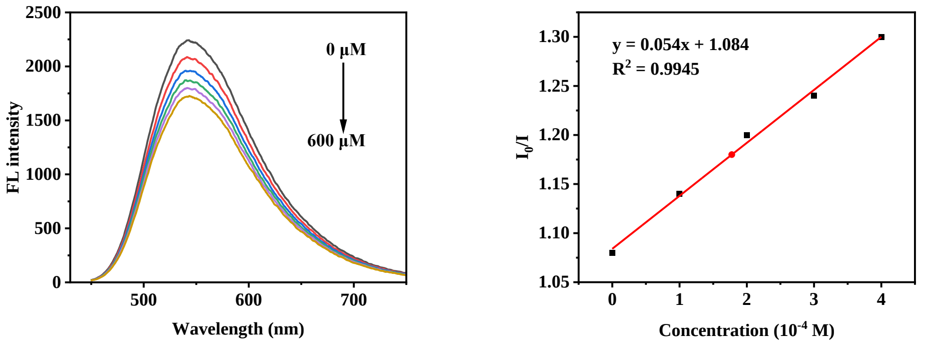

Figure S8 Emission spectra of the 4CzIPN with increasing concentrations of Ni(dtbbpy)Br_2

5.5.3 Quenching of 4CzIPN with Co(TPP)

Excitation wavelength: 440 nm.

Excitation slit: 5 nm; emission slit: 5 nm.

To a standard solution of 4CzIPN in DMAc (0.1 mM) were added different amounts of a solution of Co(TPP) in DMAc to afford the final concentrations reported in the figure below. The fluorescence of the solutions was then measured. DMAc was degassed with a stream of nitrogen for 30 min.

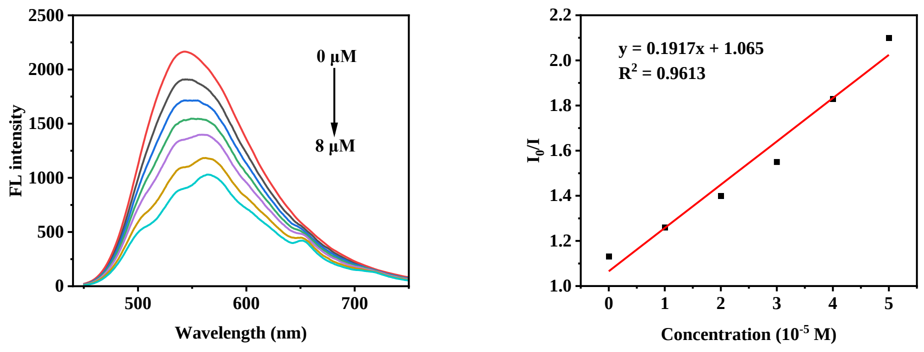

Figure S9 Emission spectra of the 4CzIPN with increasing concentrations of Co(TPP)

5.5.4 Quenching of Co(TPP) with Hantzsch ester

Excitation wavelength: 440 nm.

Excitation slit: 5 nm; emission slit: 5 nm.

To a standard solution of Co(TPP) in DMAc (0.1 mM) were added different amounts of a solution of Hantzsch ester in DMAc to afford the final concentrations reported in the figure below. The fluorescence of the solutions was then measured. DMAc was degassed with a stream of nitrogen for 30 min.

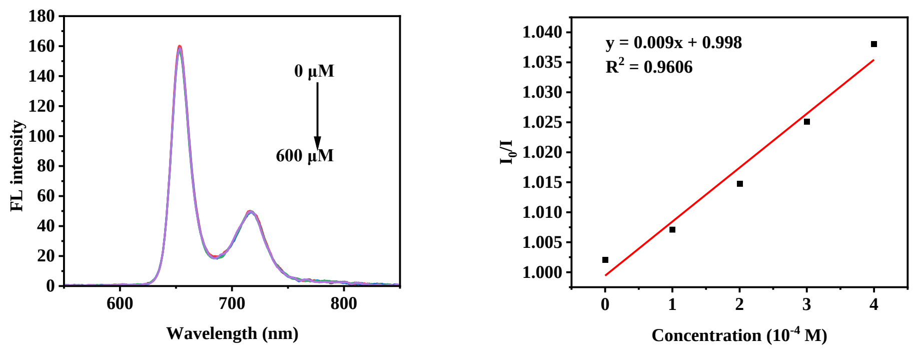

Figure S10 Emission spectra of the Co(TPP) with increasing concentrations of Hantzsch ester

6. An Alternative Mechanism

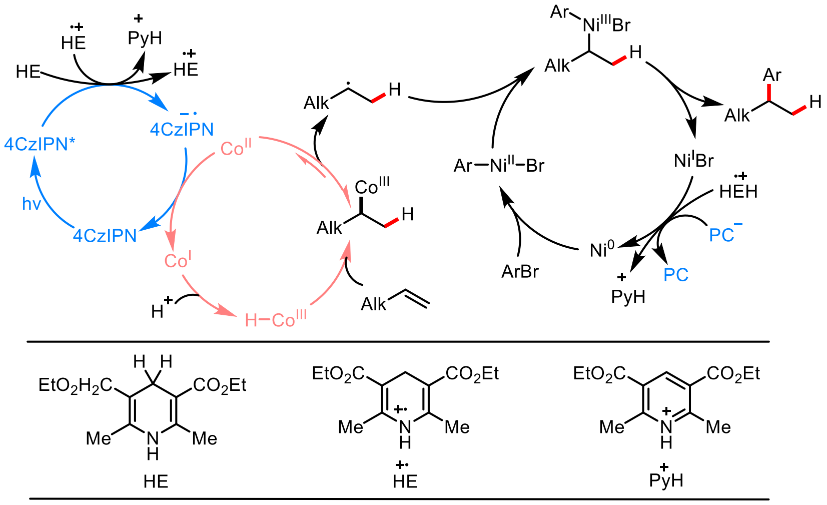

Figure S11 An alternative mechanism

7. References

1 J. Qin, M. Barday, S. Jana, N. Sanosa, I. Funes-Ardoiz and C. J. Teskey, *Angew. Chem. Int. Ed.*, 2023, **62**, e202310639.

2 E. Bergamaschi, V. J. Mayerhofer and C. J. Teskey, *ACS Catal.*, 2022, **12**, 14806-14811.

8. ^{1}H, ^{13}C and ^{19}F NMR spectra of all products

Compound 3aa ^{1}H NMR (400 MHz, $CDCl_3$)

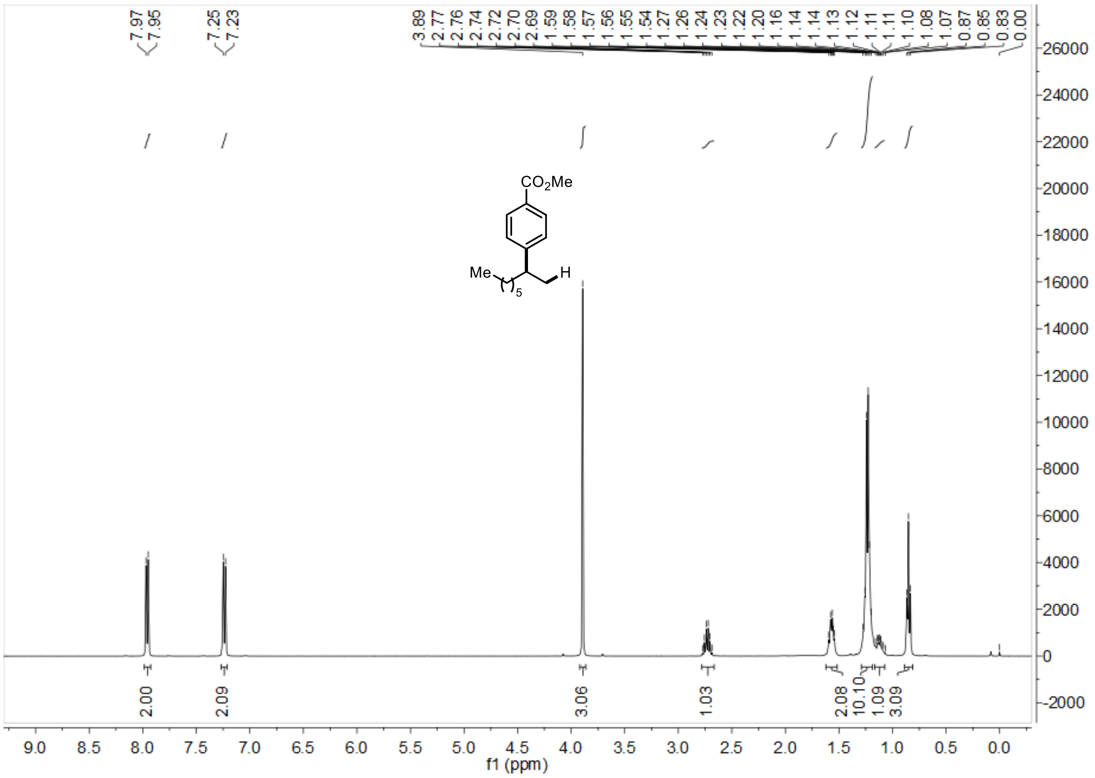

Compound 3aa ^{13}C NMR (101 MHz, $CDCl_3$)

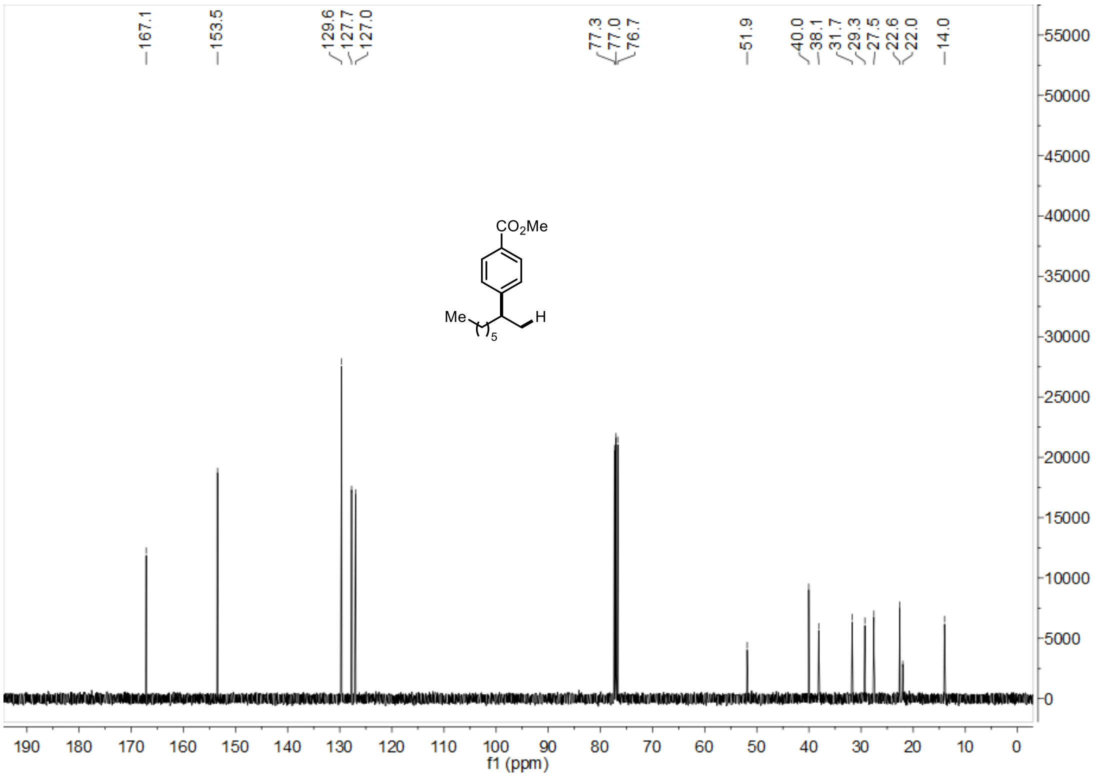

Compound 3ab ^1H NMR (400 MHz, $CDCl_3$)

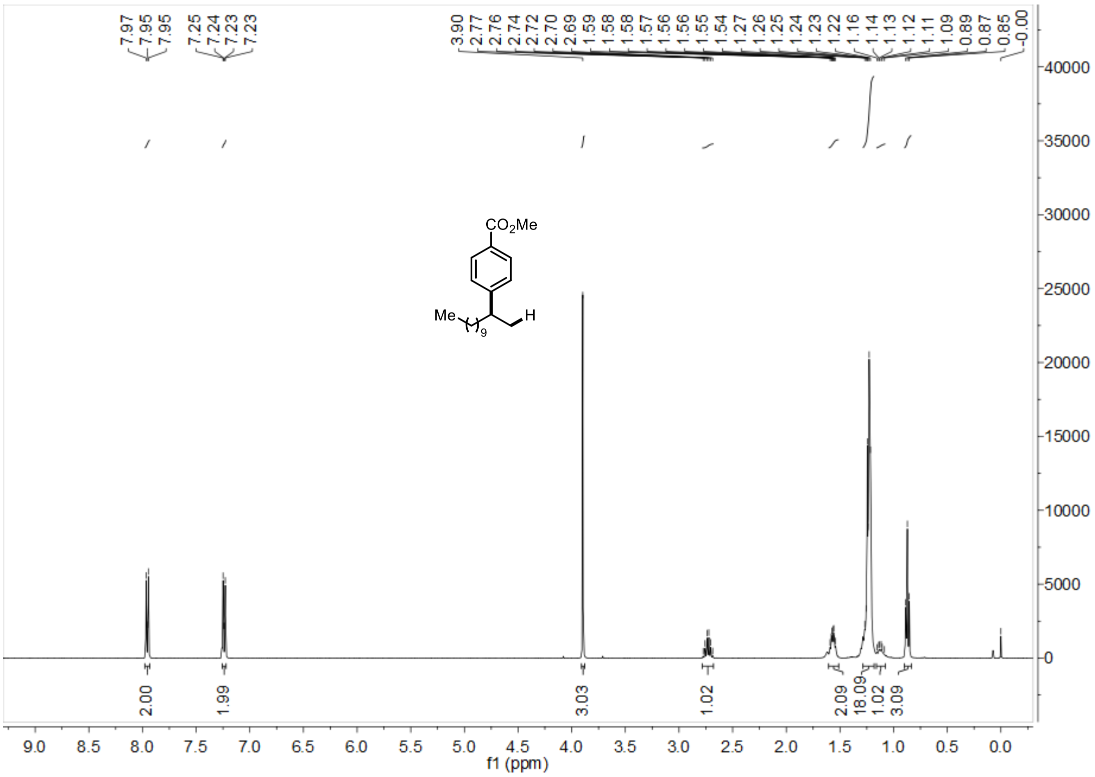

Compound 3ab ^{13}C NMR (101 MHz, $CDCl_3$)

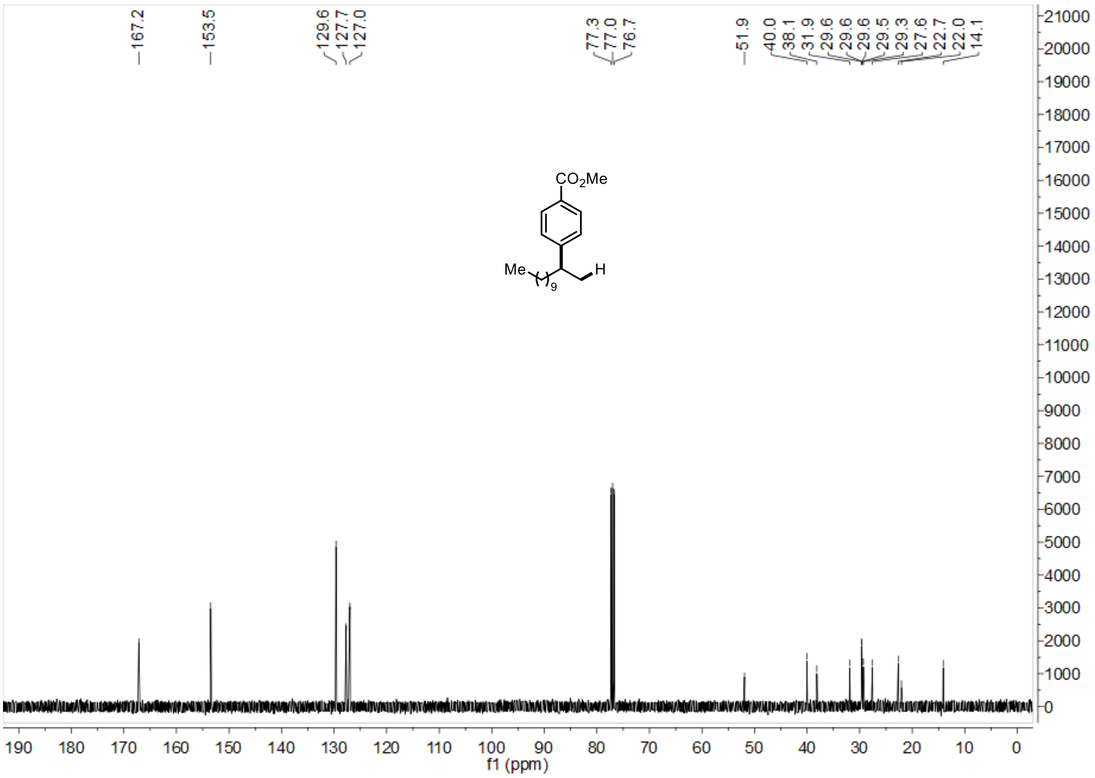

Compound 3ac ^{1}H NMR (400 MHz, $CDCl_3$)

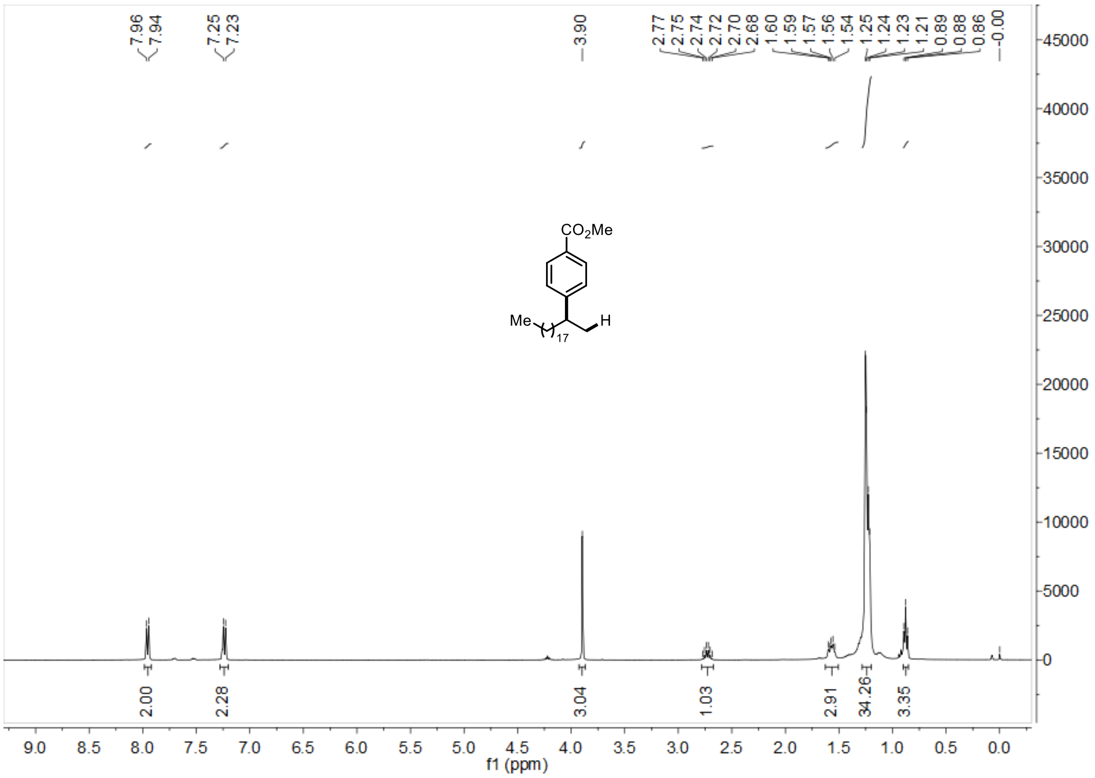

Compound 3ac ^{13}C NMR (101 MHz, $CDCl_3$)

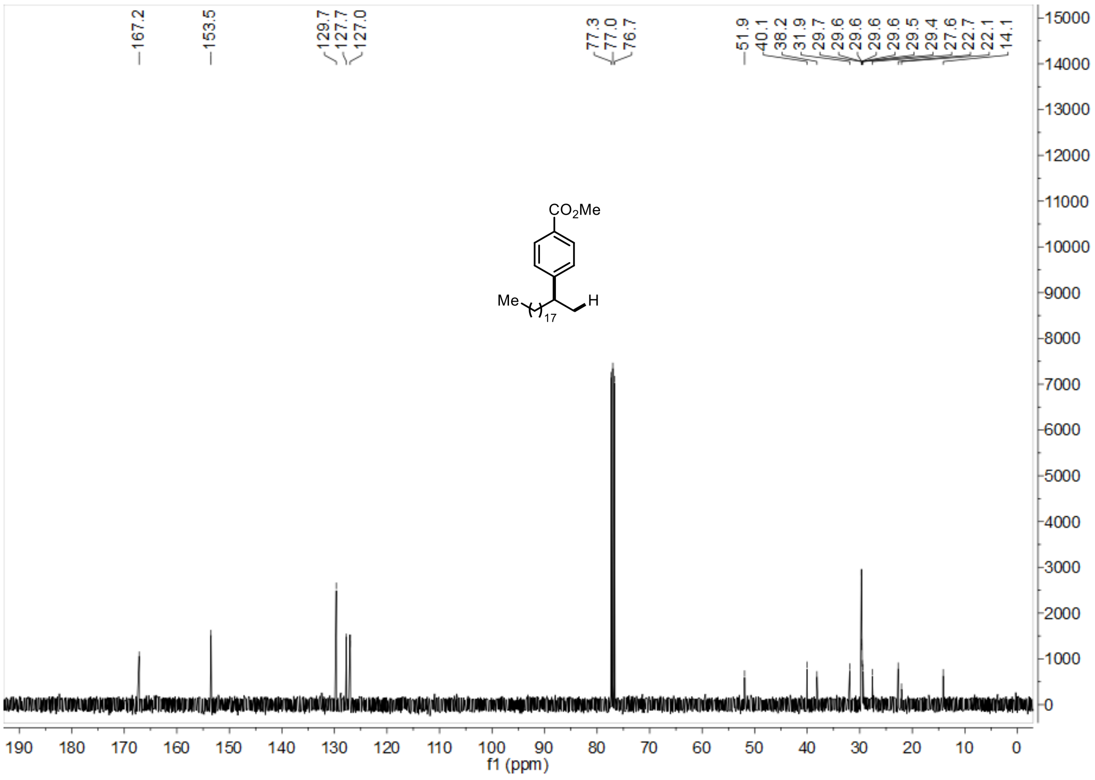

Compound 3ad ^{1}H NMR (400 MHz, $CDCl_3$)

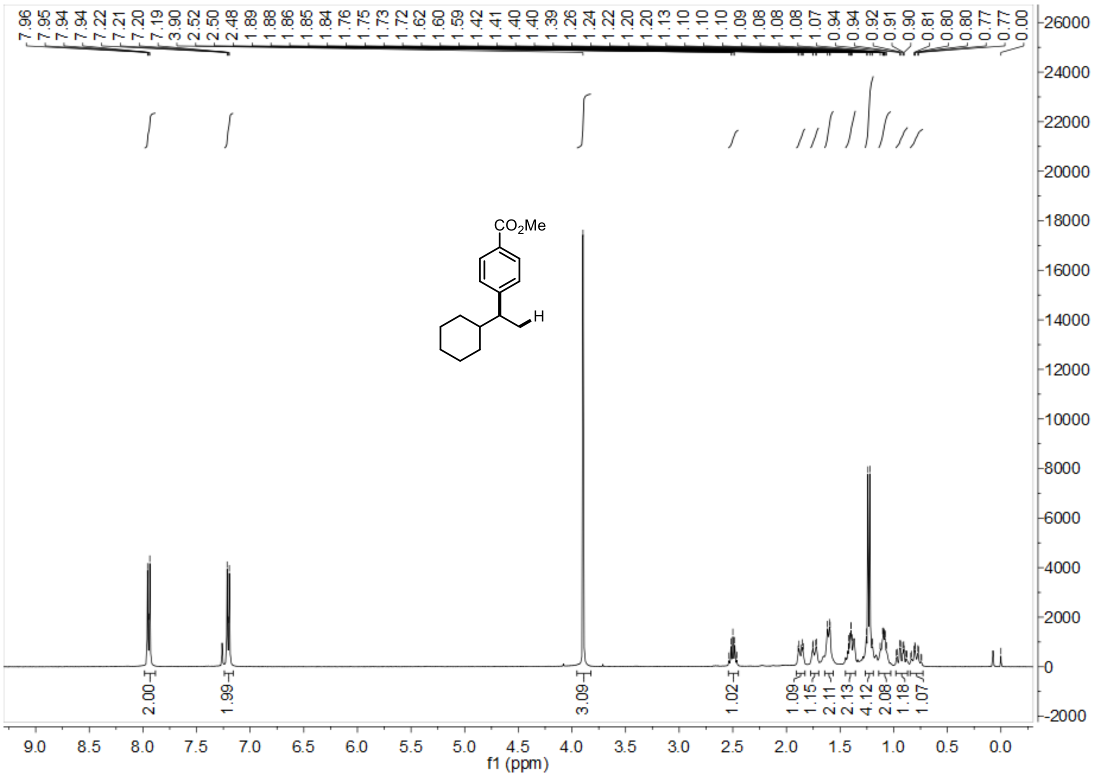

Compound 3ad ^{13}C NMR (101 MHz, $CDCl_3$)

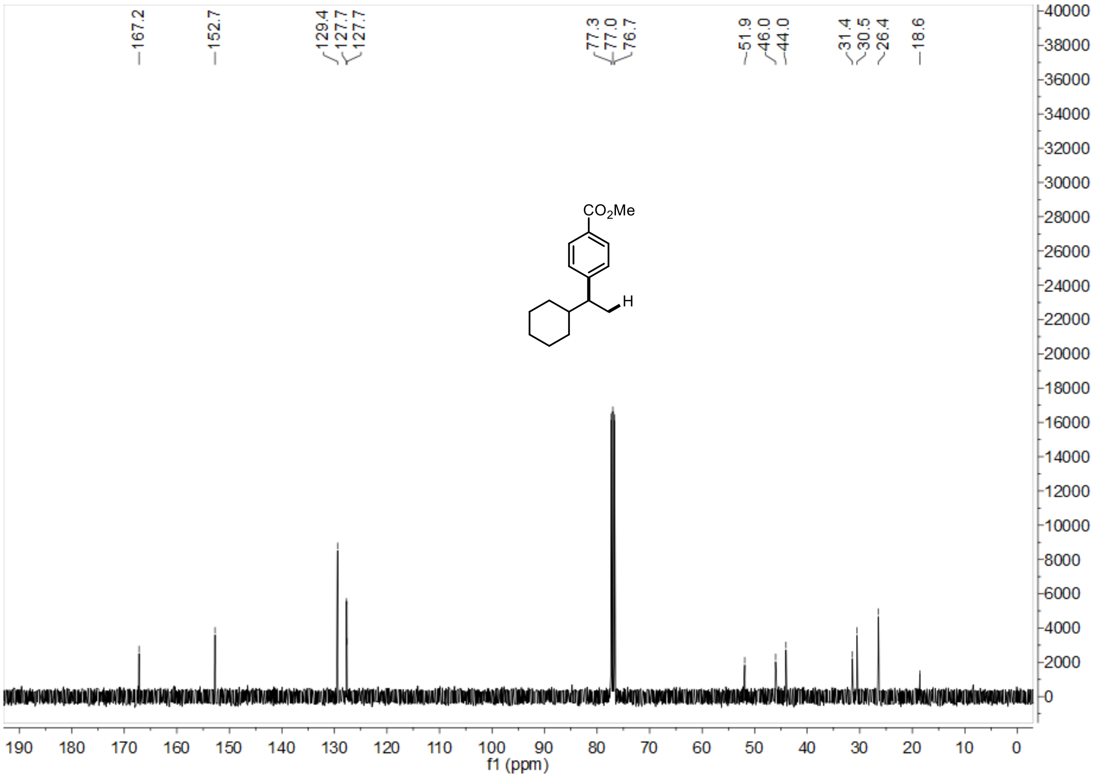

Compound 3ae ^{1}H NMR (400 MHz, $CDCl_3$)

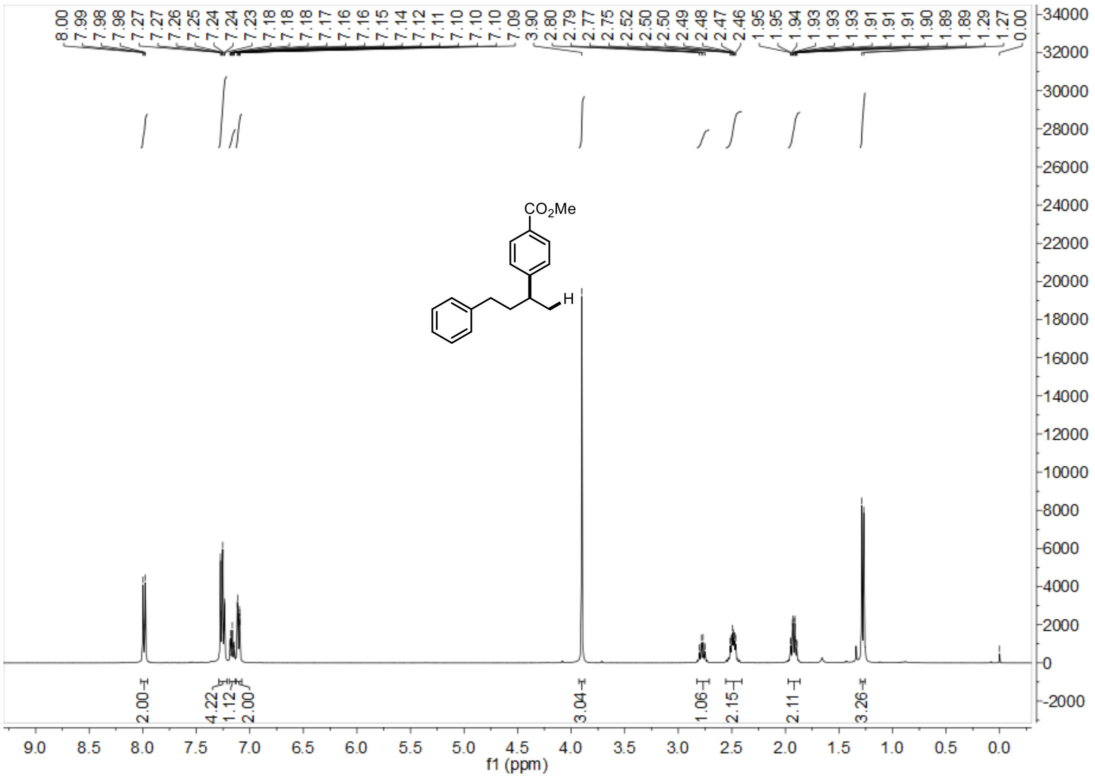

Compound 3ae ^{13}C NMR (101 MHz, $CDCl_3$)

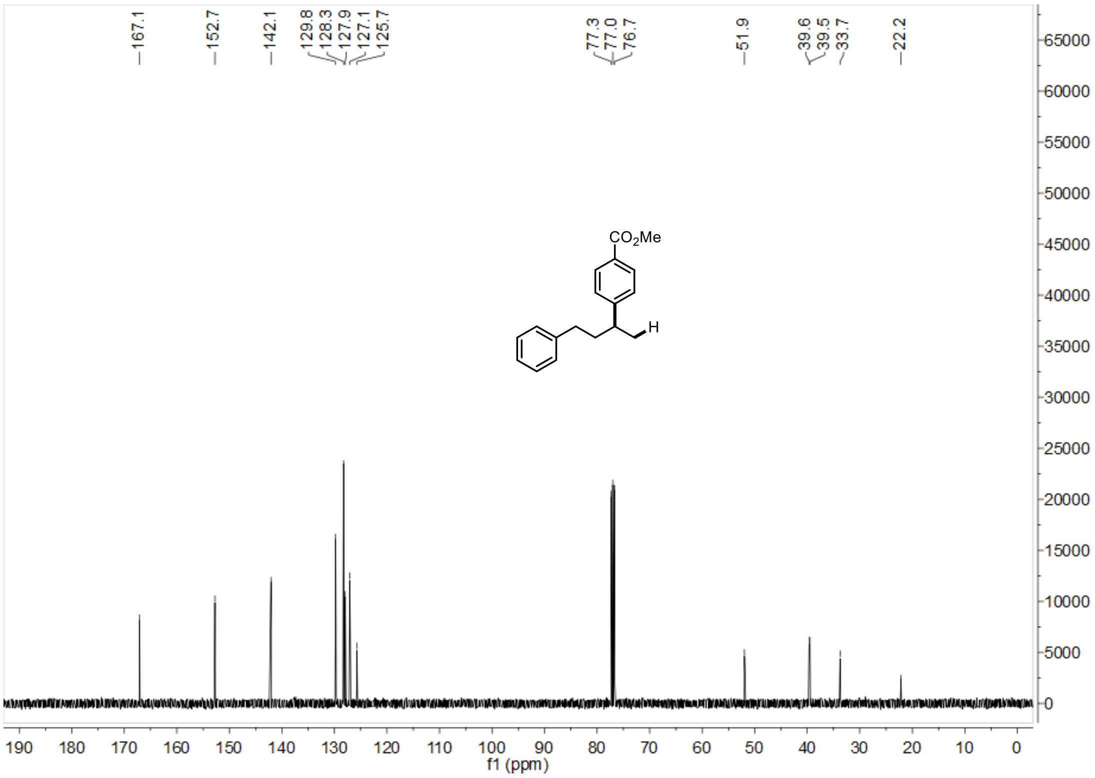

Compound 3af ^{1}H NMR (400 MHz, $CDCl_3$)

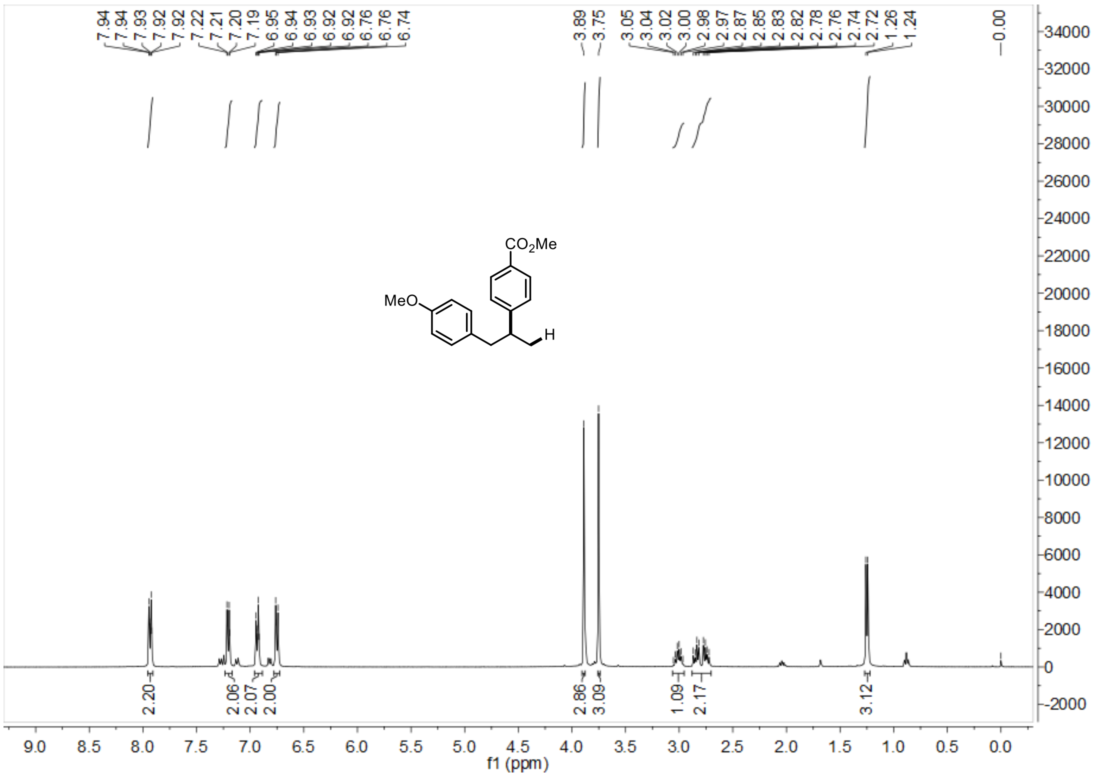

Compound 3af ^{13}C NMR (101 MHz, $CDCl_3$)

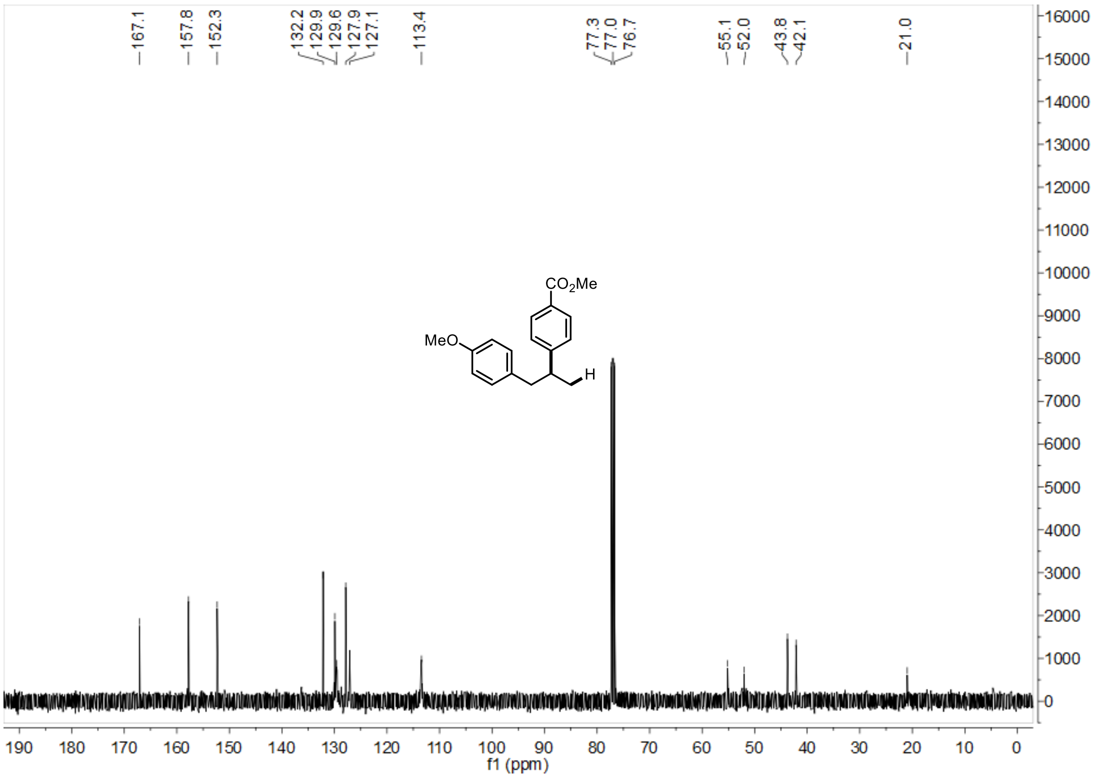

Compound 3ag ^{1}H NMR (400 MHz, $CDCl_3$)

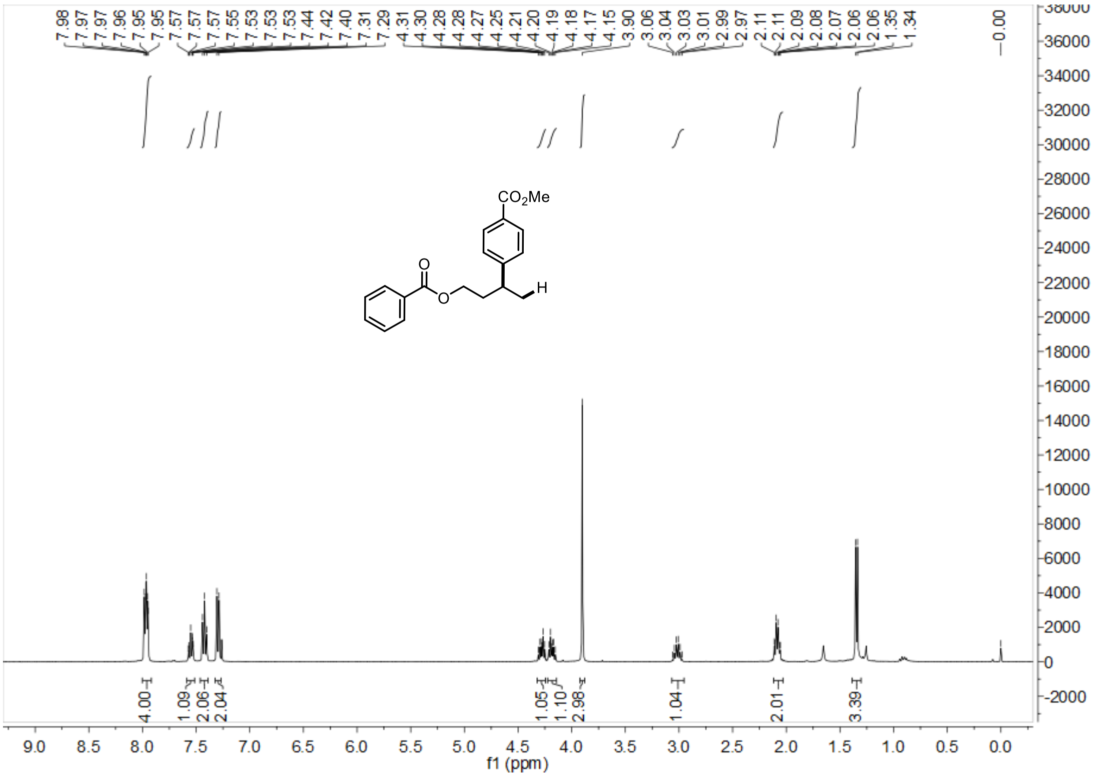

Compound 3ag ^{13}C NMR (101 MHz, $CDCl_3$)

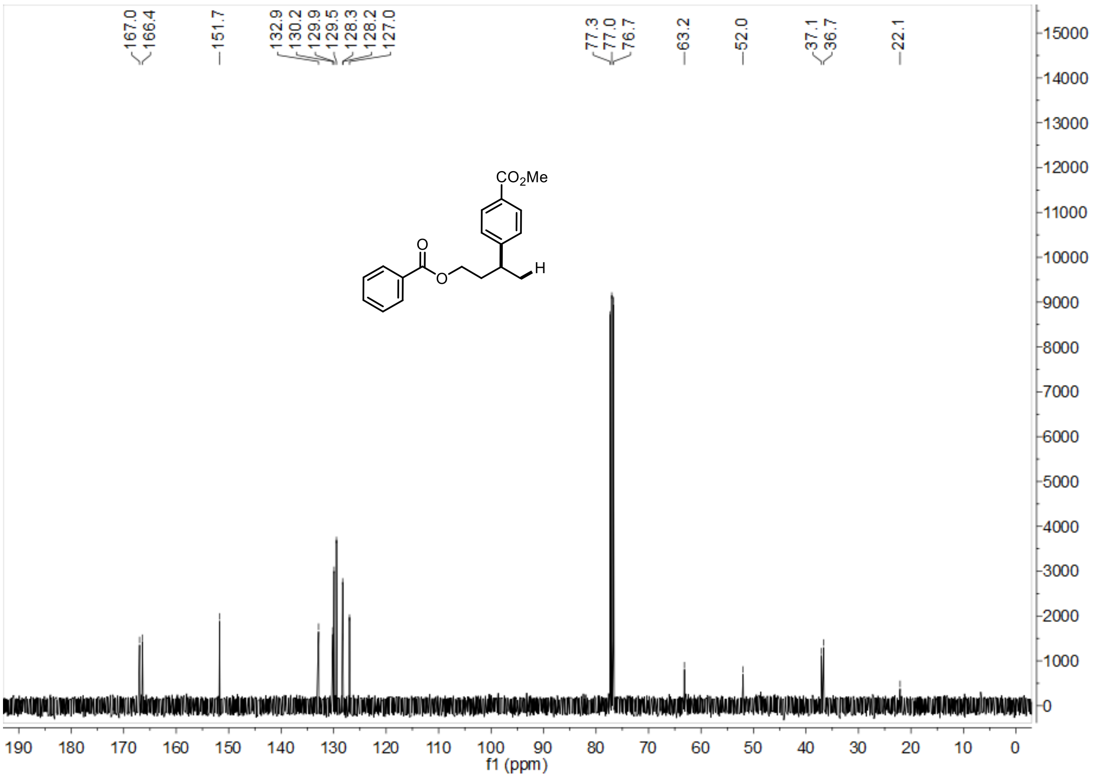

Compound 3ah ^{1}H NMR (400 MHz, $CDCl_3$)

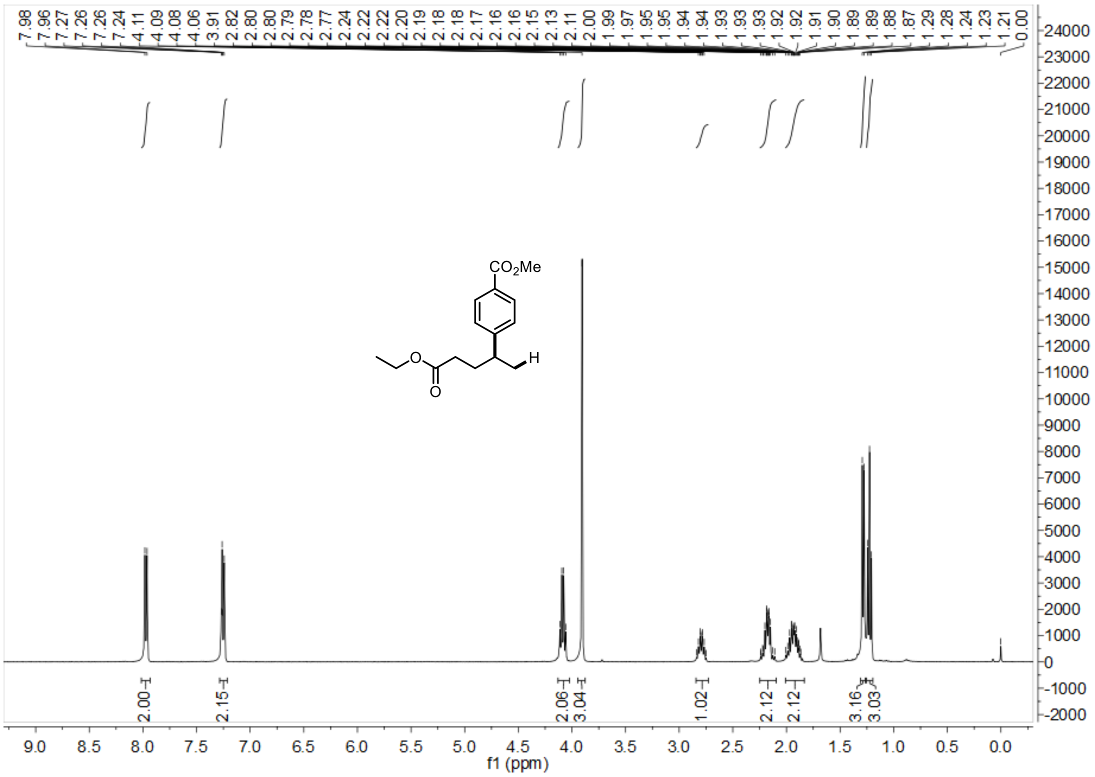

Compound 3ah ^{13}C NMR (101 MHz, $CDCl_3$)

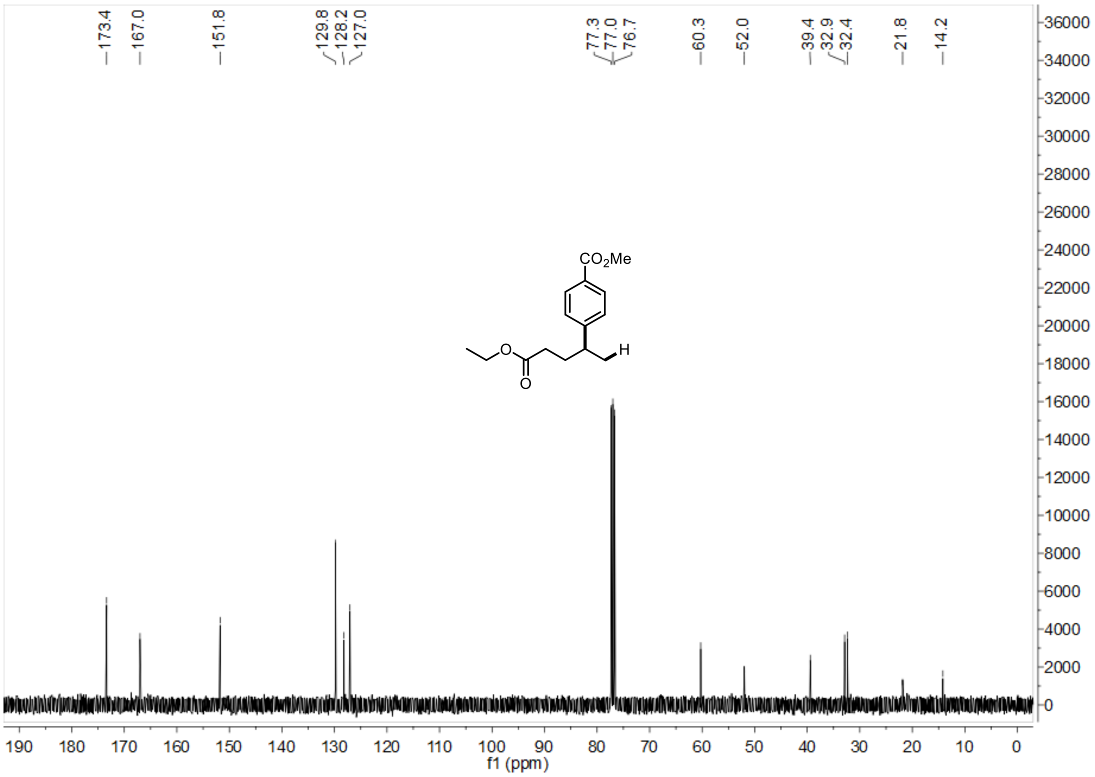

Compound 3ai ^{1}H NMR (400 MHz, $CDCl_3$)

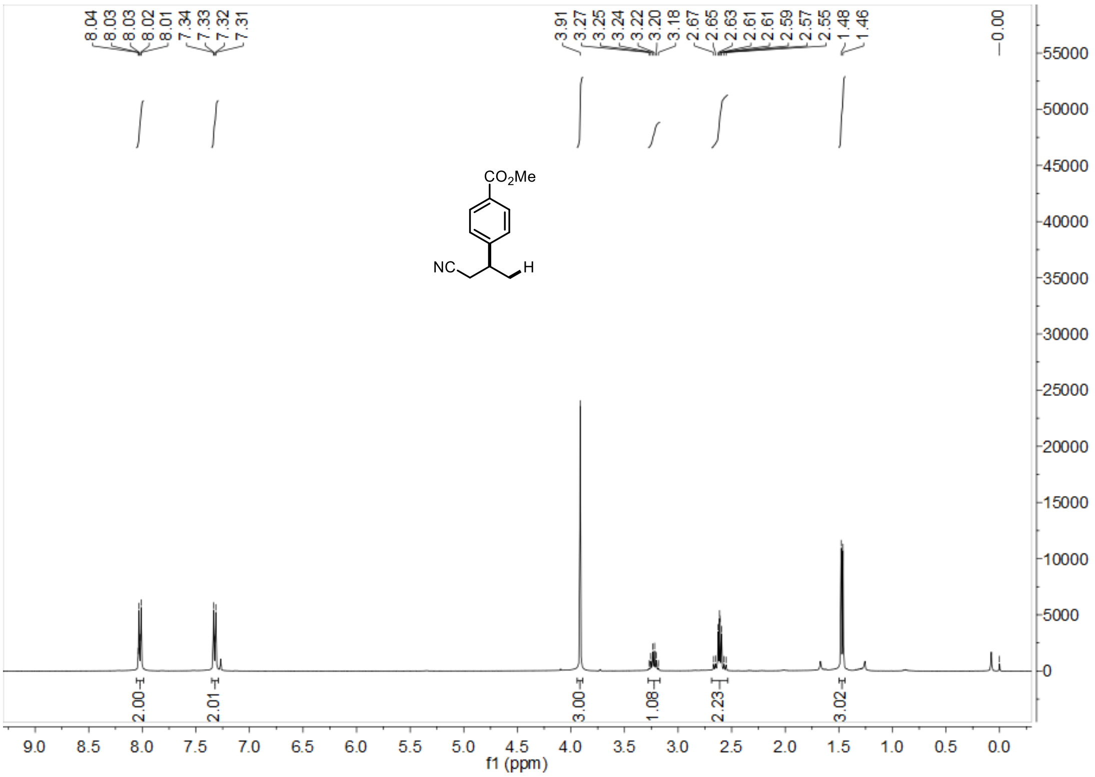

Compound 3ai ^{13}C NMR (101 MHz, $CDCl_3$)

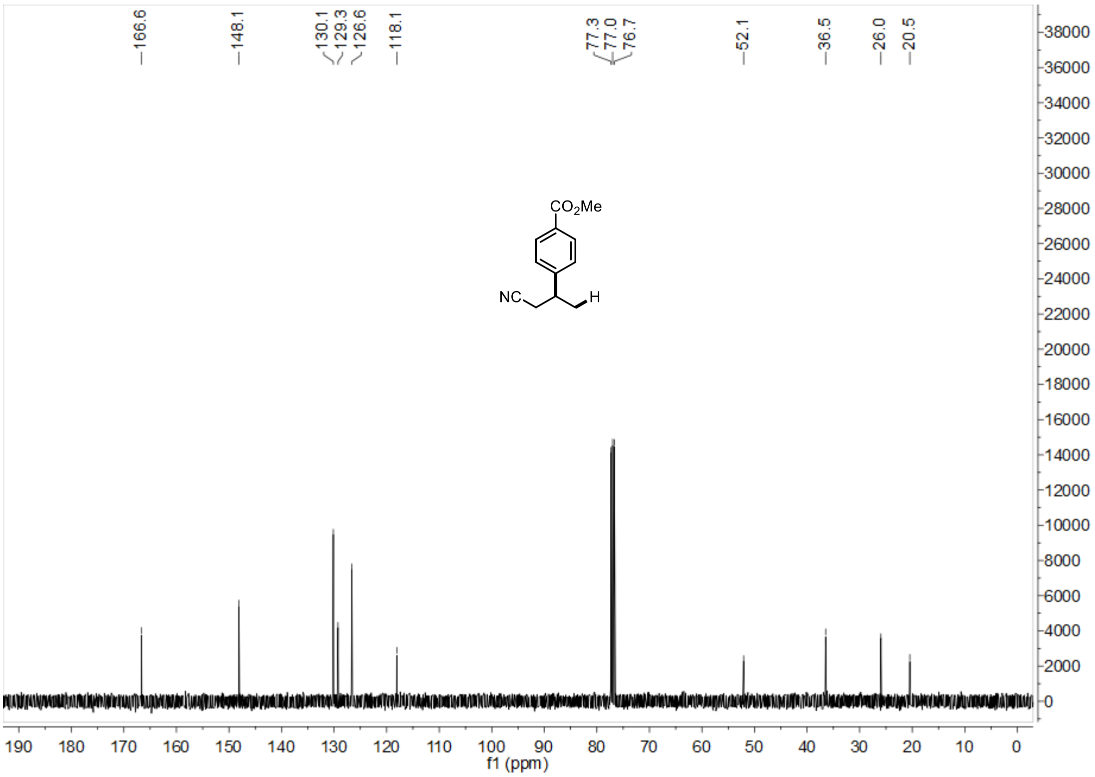

Compound 3aj ¹H NMR (400 MHz, $CDCl_3$)

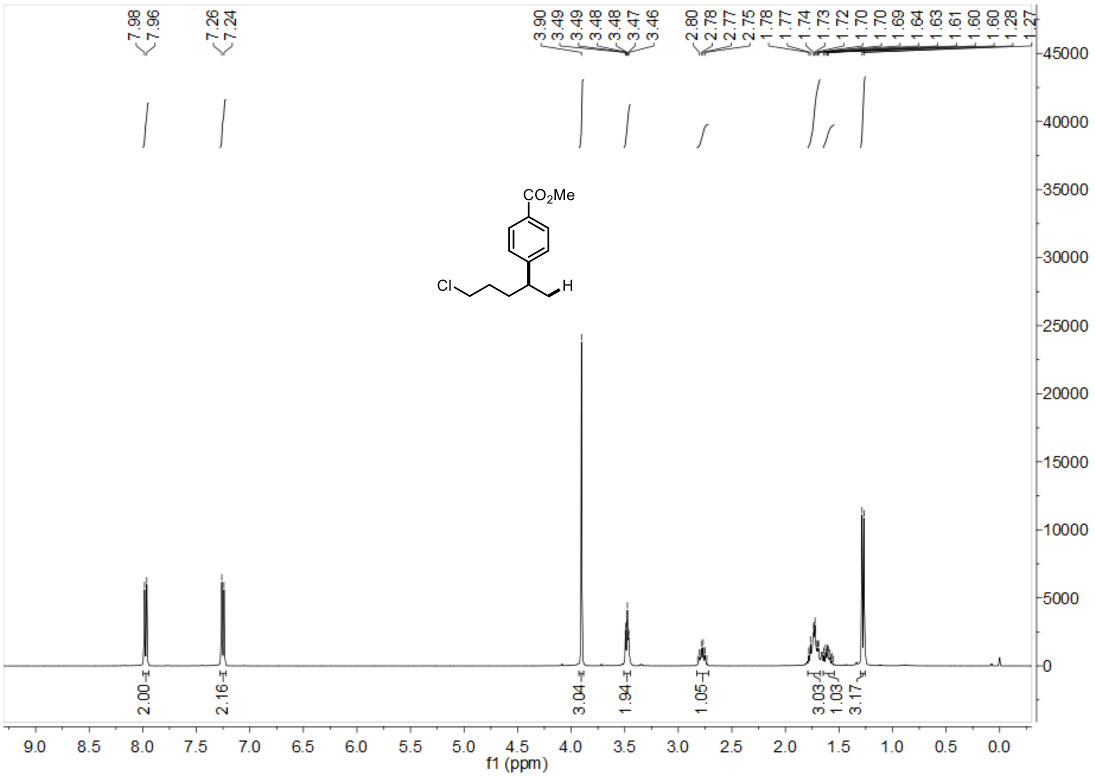

Compound 3aj ¹³C NMR (101 MHz, $CDCl_3$)

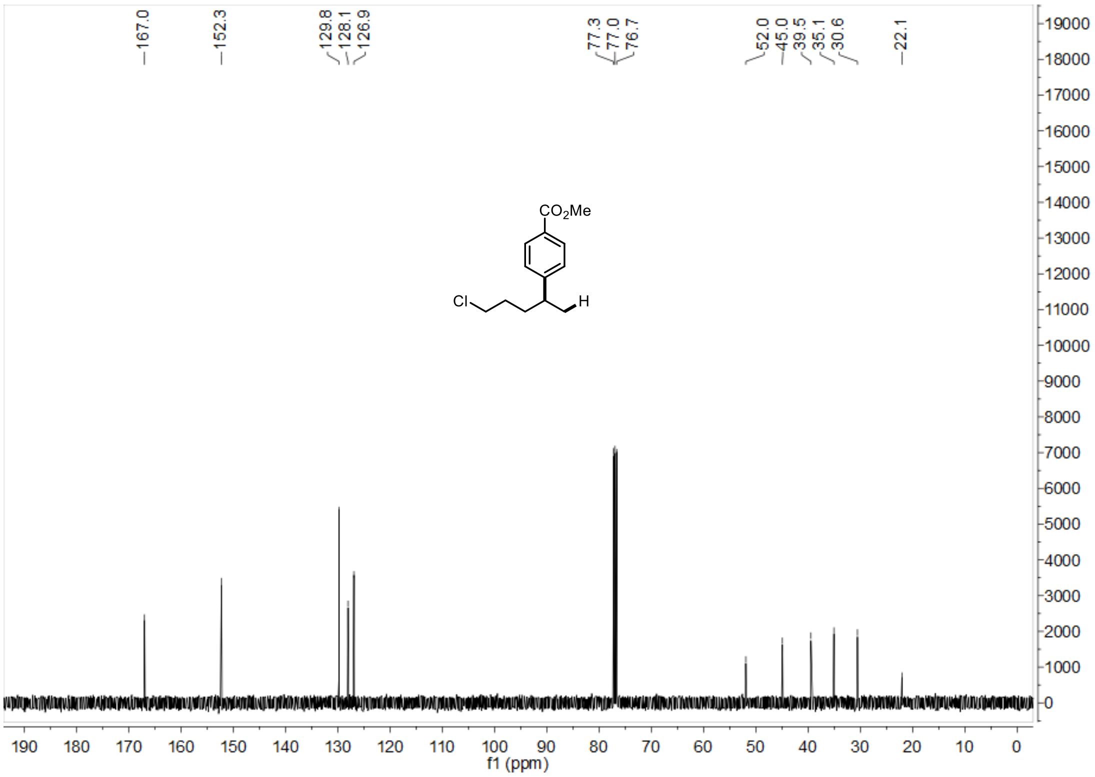

Compound 3ak ^1H NMR (400 MHz, $CDCl_3$)

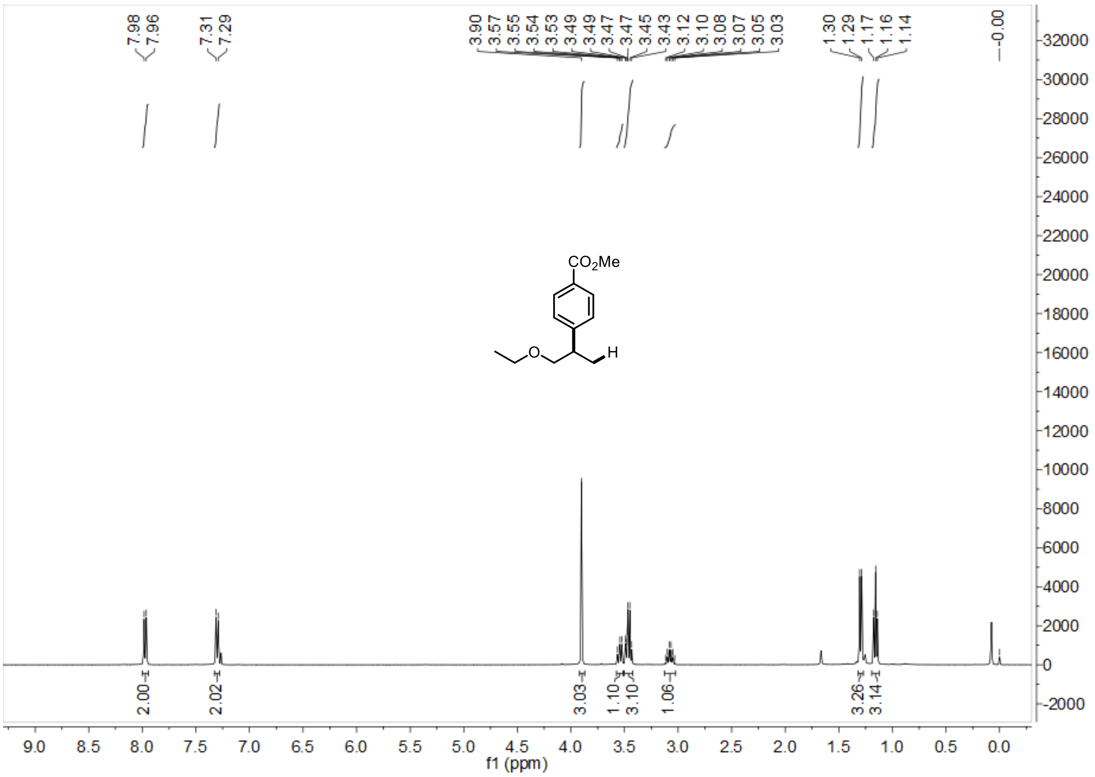

Compound 3ak ^{13}C NMR (101 MHz, $CDCl_3$)

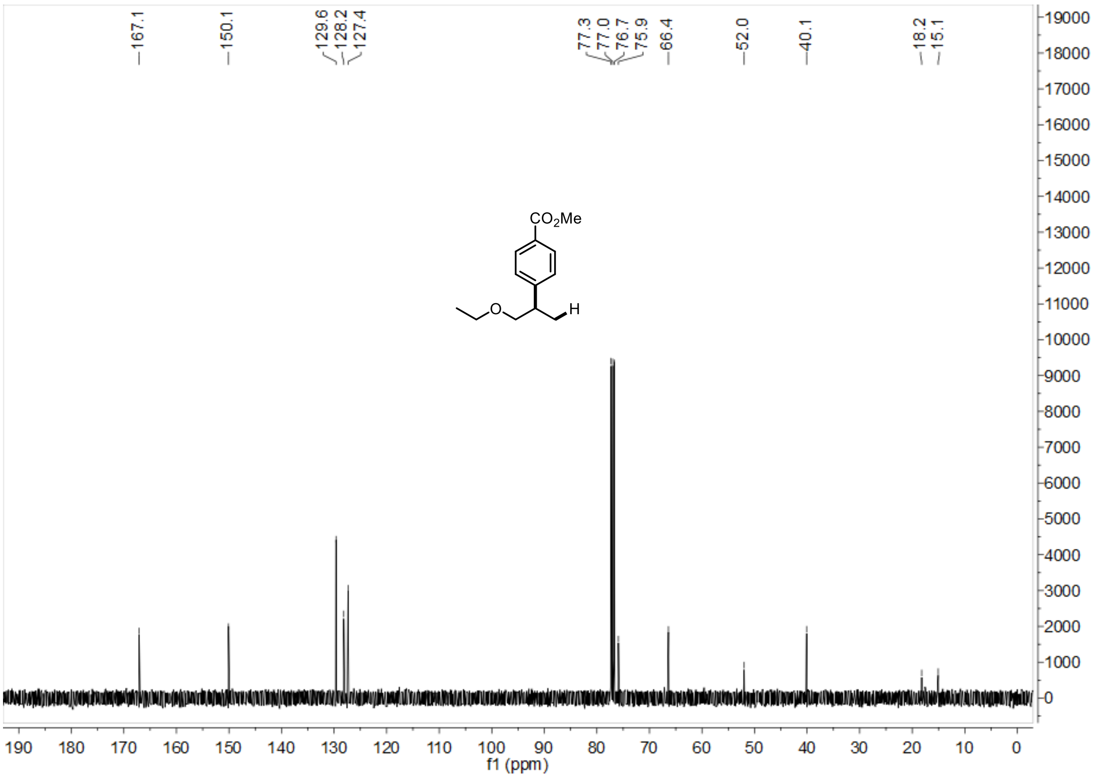

Compound 3al ^{1}H NMR (400 MHz, $CDCl_3$)

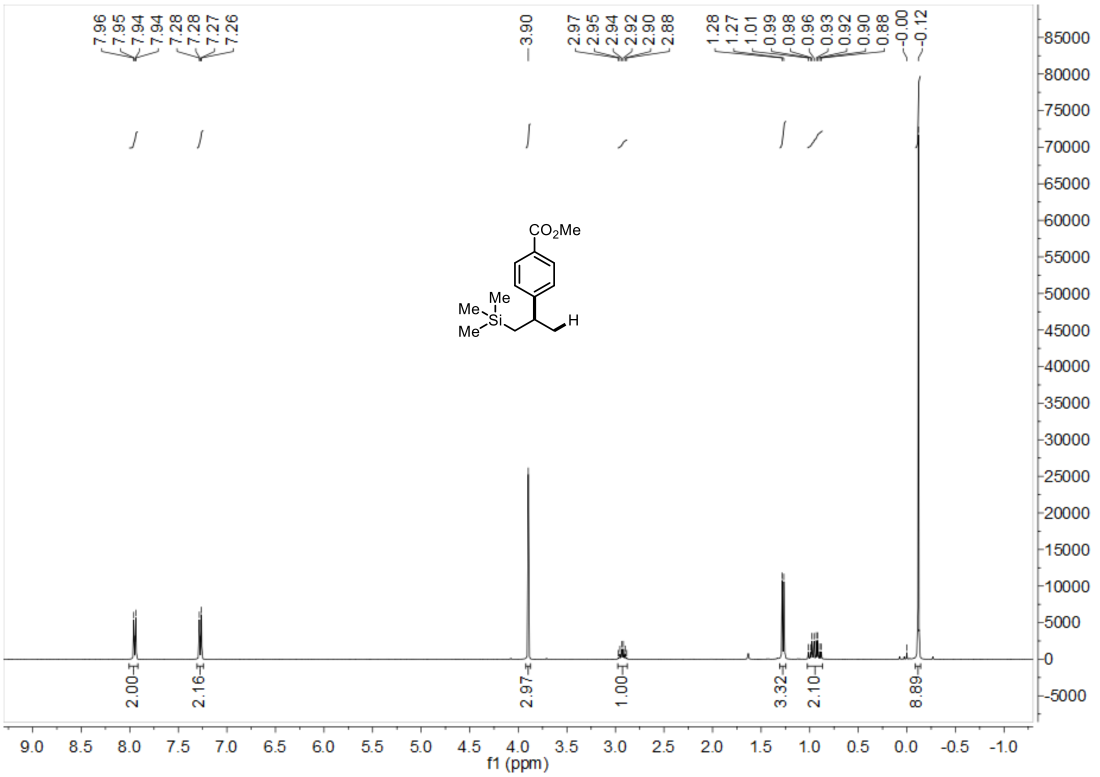

Compound 3al ^{13}C NMR (101 MHz, $CDCl_3$)

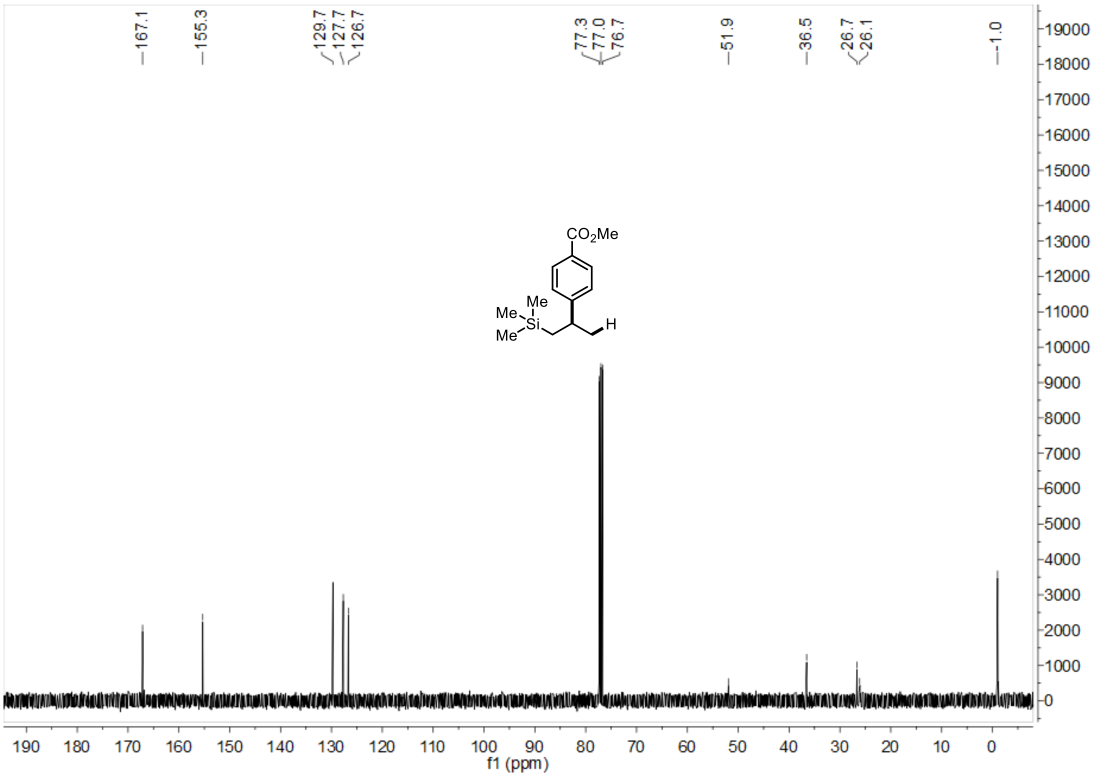

Compound 3am ¹H NMR (400 MHz, CDCl3)

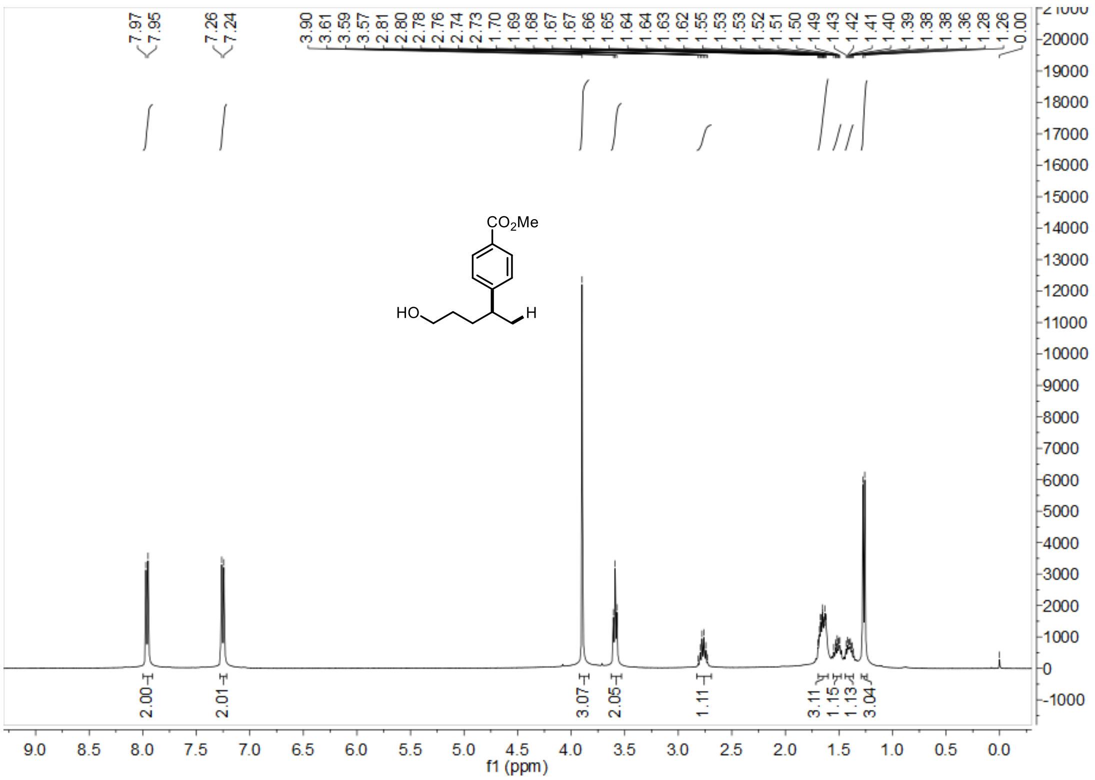

Compound 3am ¹³C NMR (101 MHz, CDCl3)

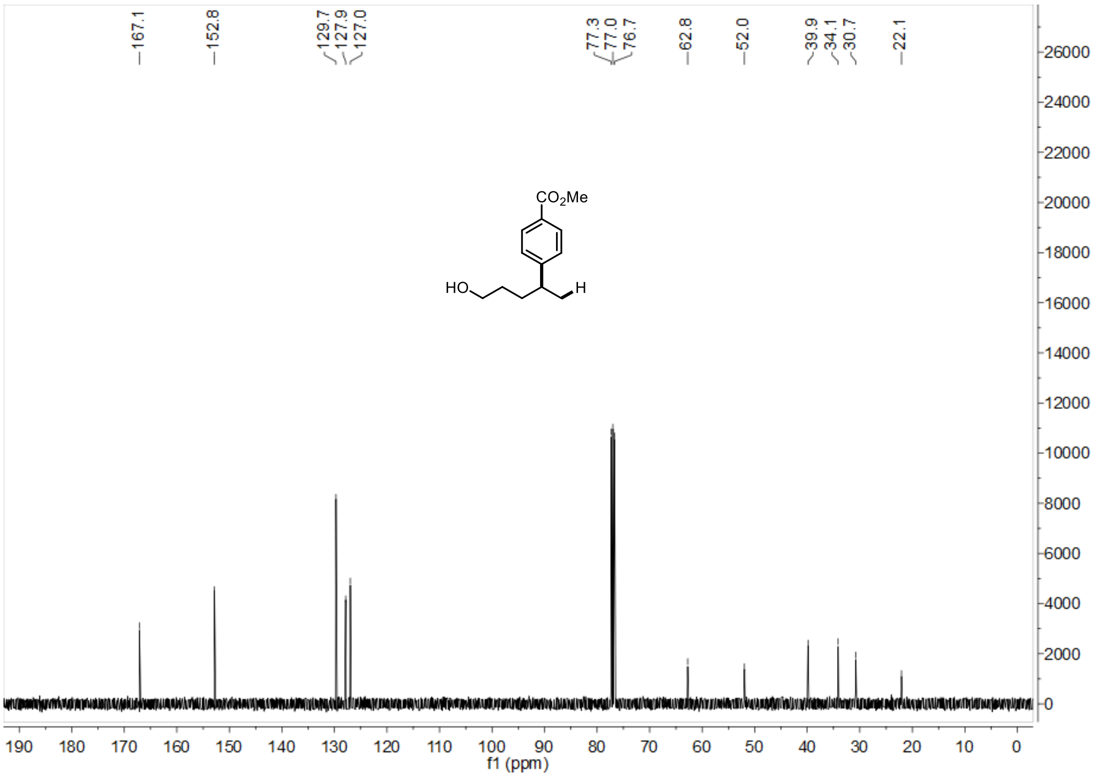

Compound 3an ^{1}H NMR (400 MHz, CDCl$_3$)

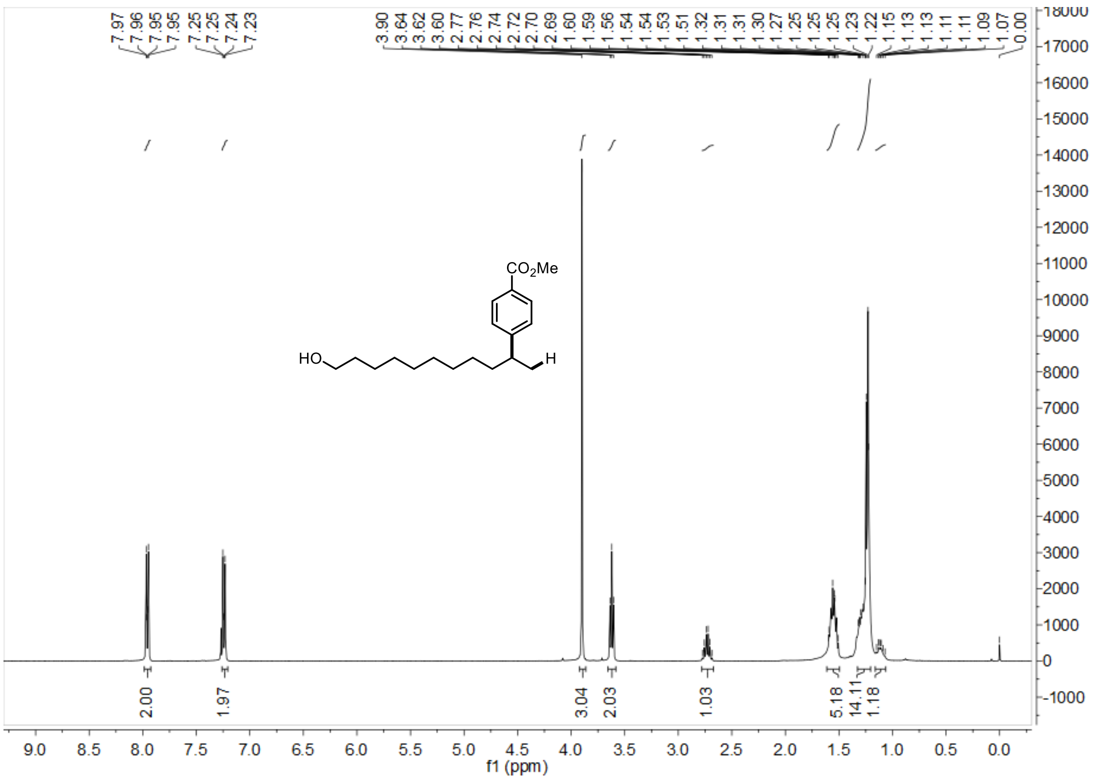

Compound 3an ^{13}C NMR (101 MHz, CDCl$_3$)

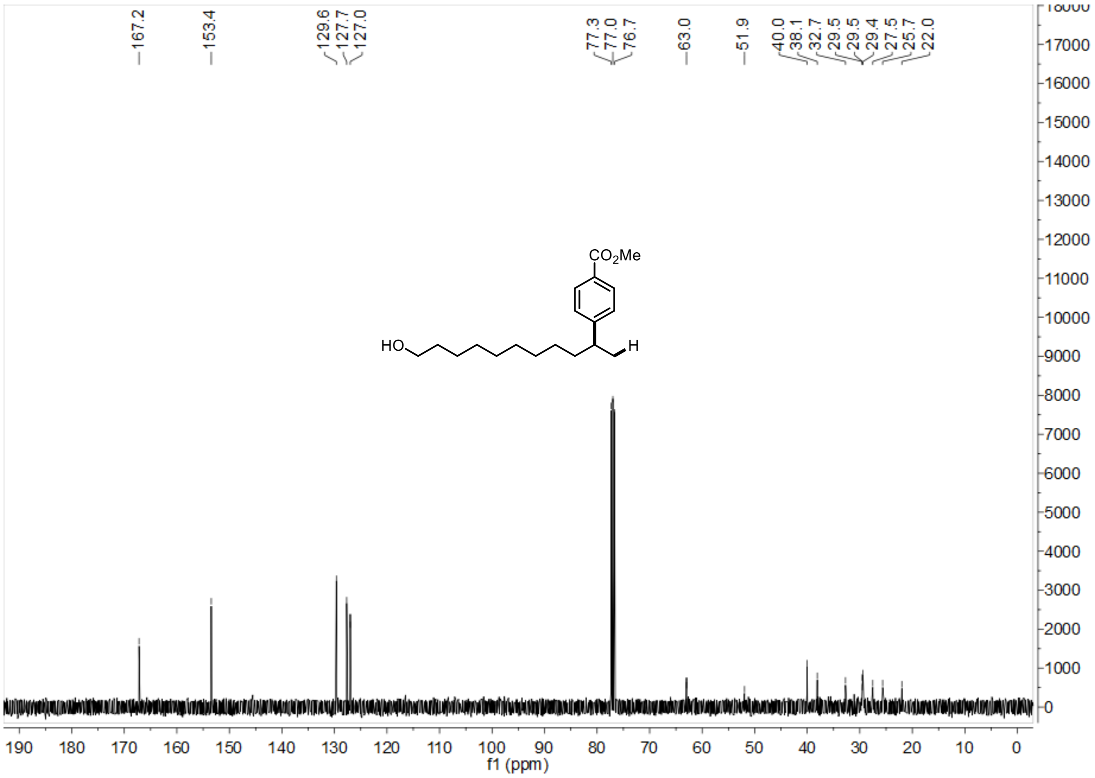

Compound 3ao ^{1}H NMR (400 MHz, $CDCl_3$)

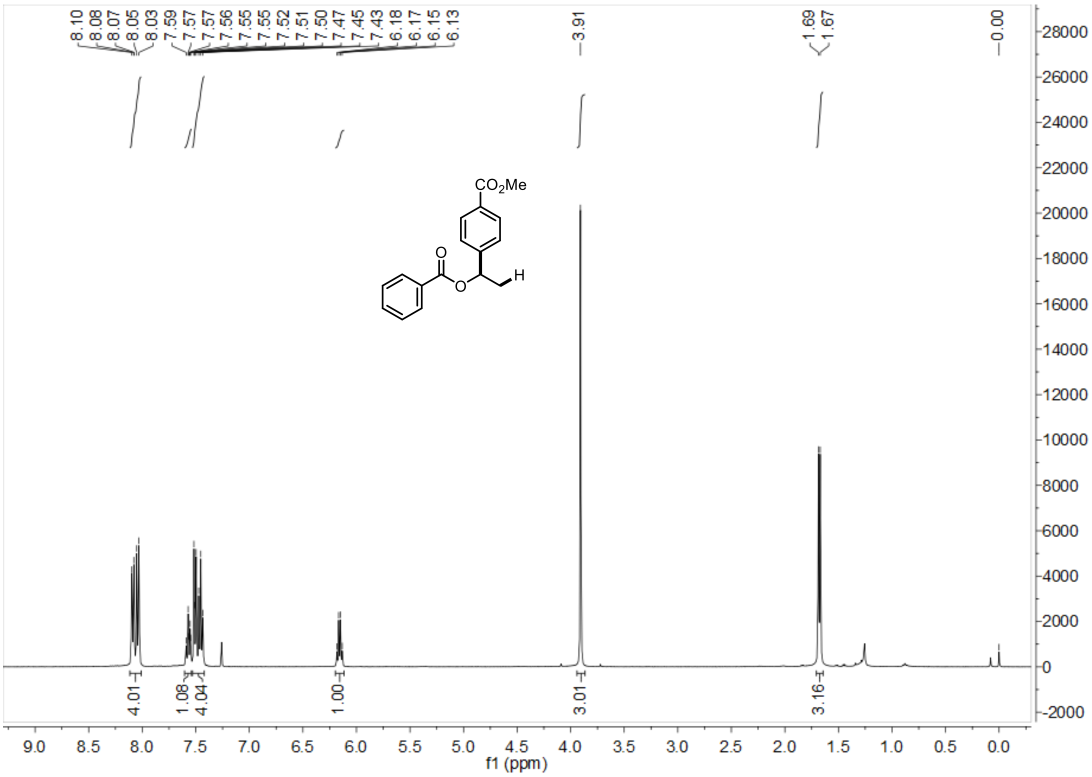

Compound 3ao ^{13}C NMR (101 MHz, $CDCl_3$)

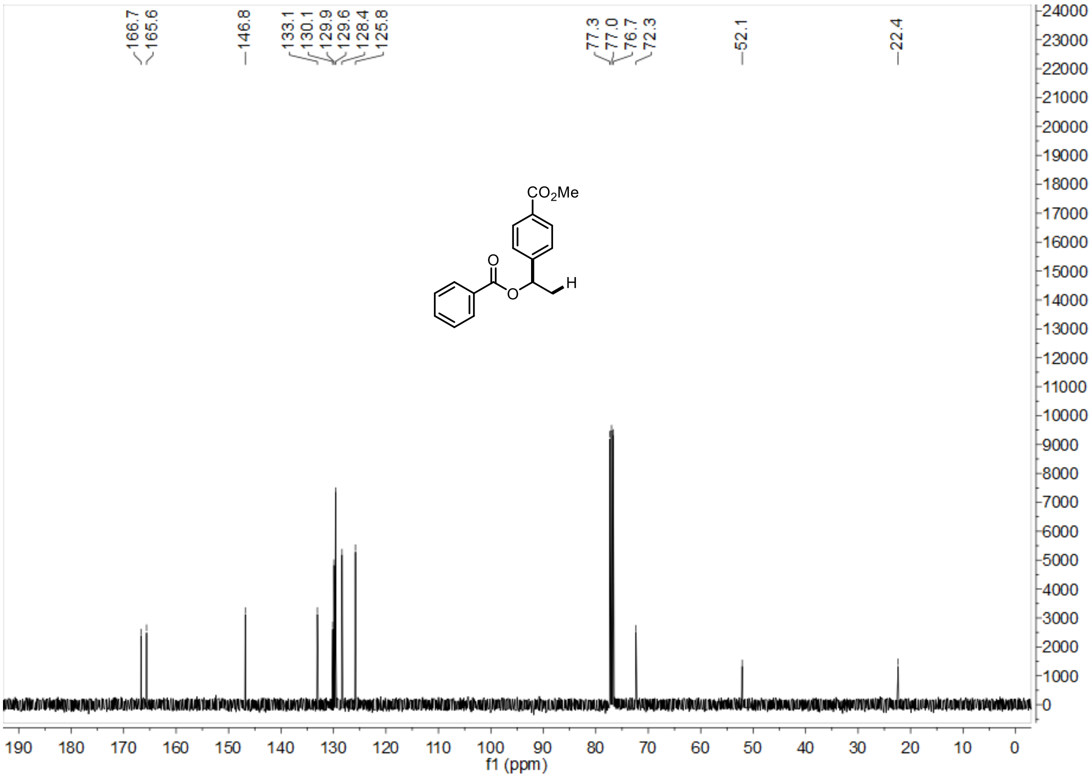

Compound 3ap ^{1}H NMR (400 MHz, $CDCl_3$)

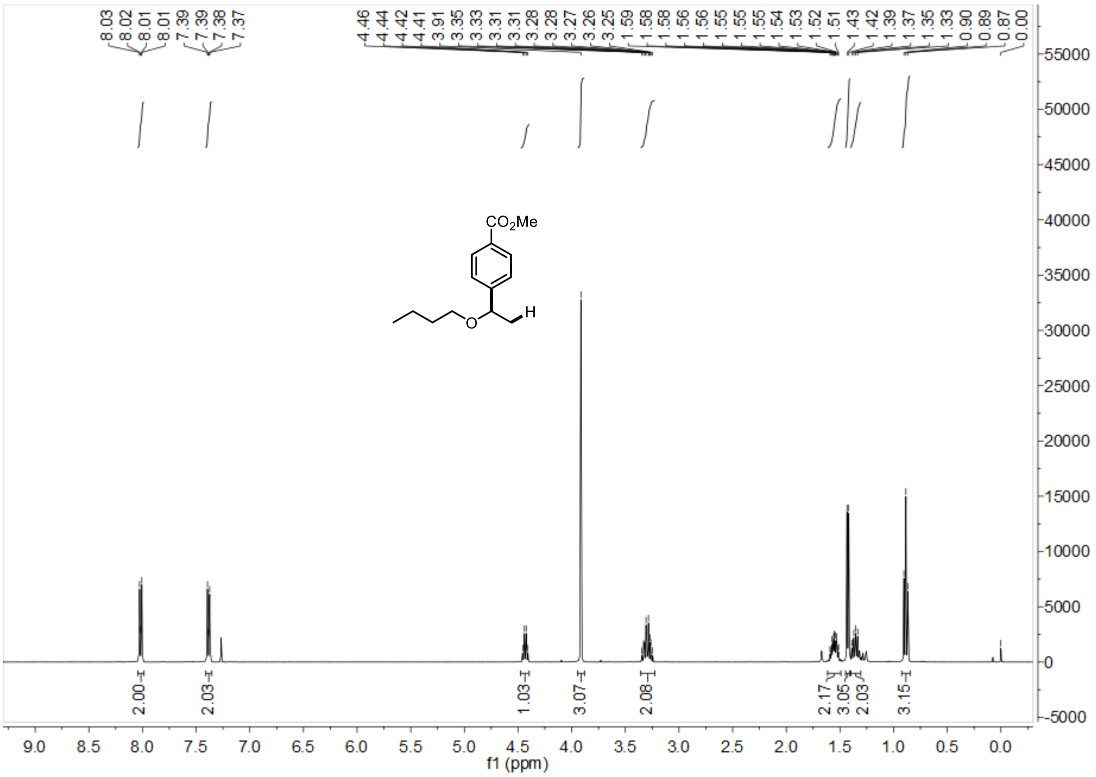

Compound 3ap ^{13}C NMR (101 MHz, $CDCl_3$)

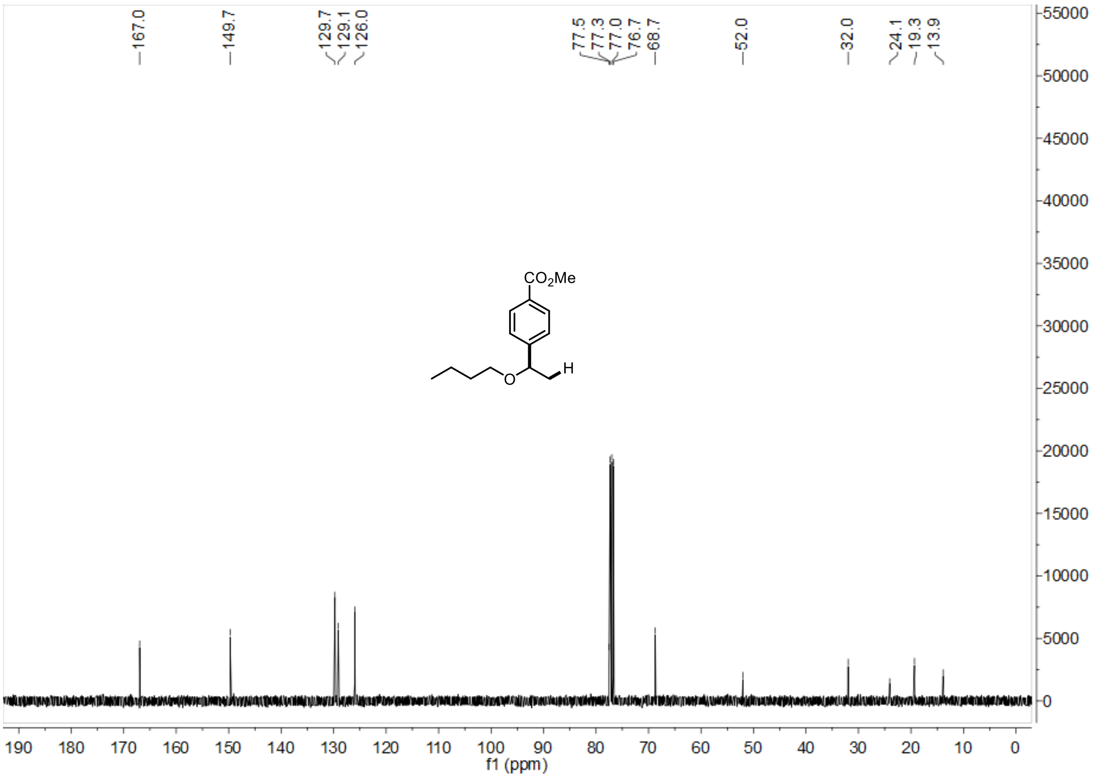

Compound 3aq ^{1}H NMR (400 MHz, CDCl3)

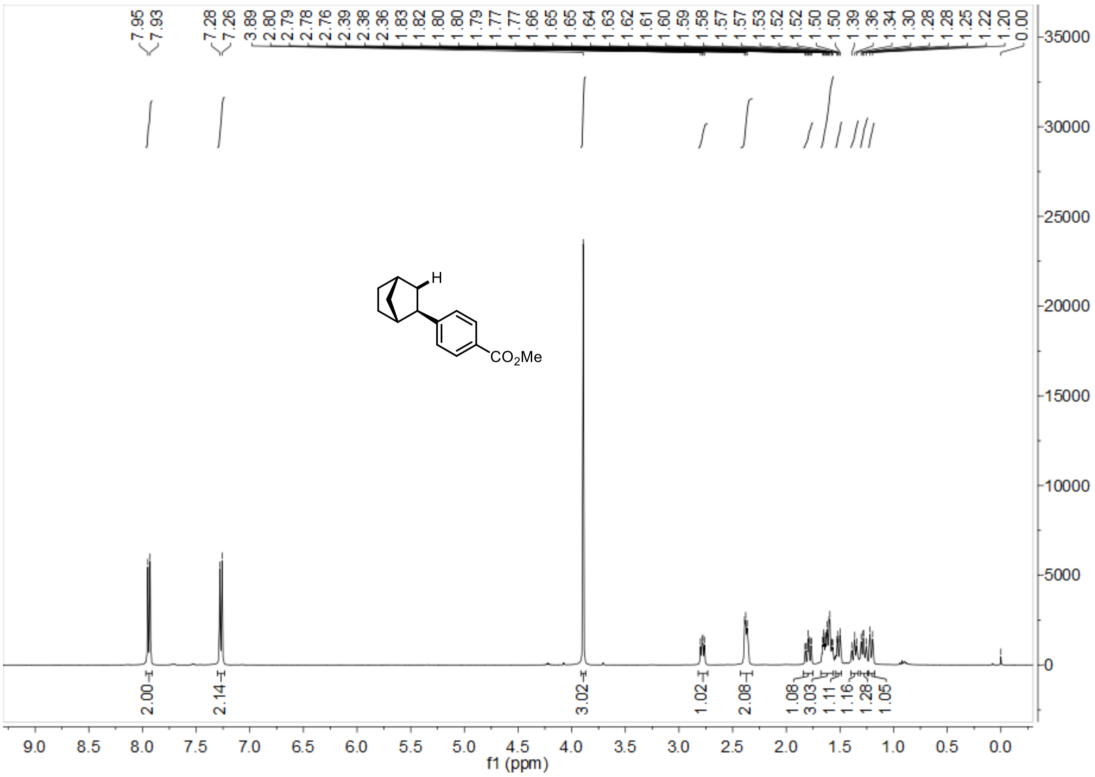

Compound 3aq ^{13}C NMR (101 MHz, CDCl3)

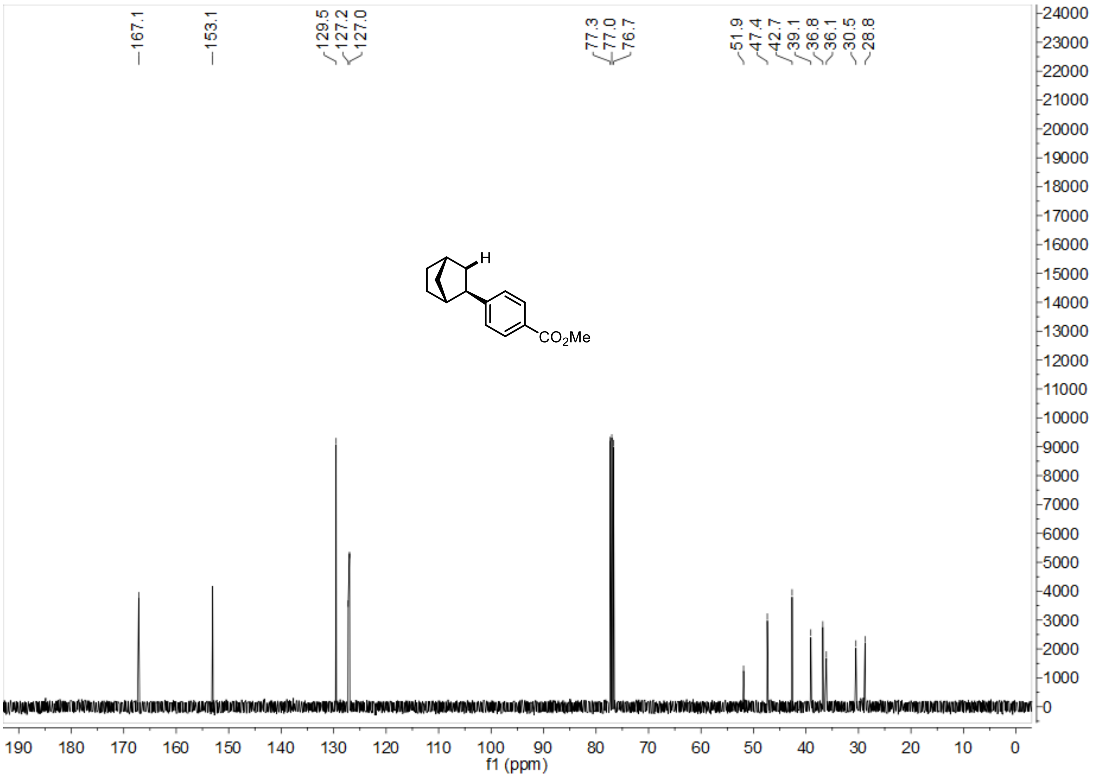

Compound 3ar ^{1}H NMR (400 MHz, $CDCl_3$)

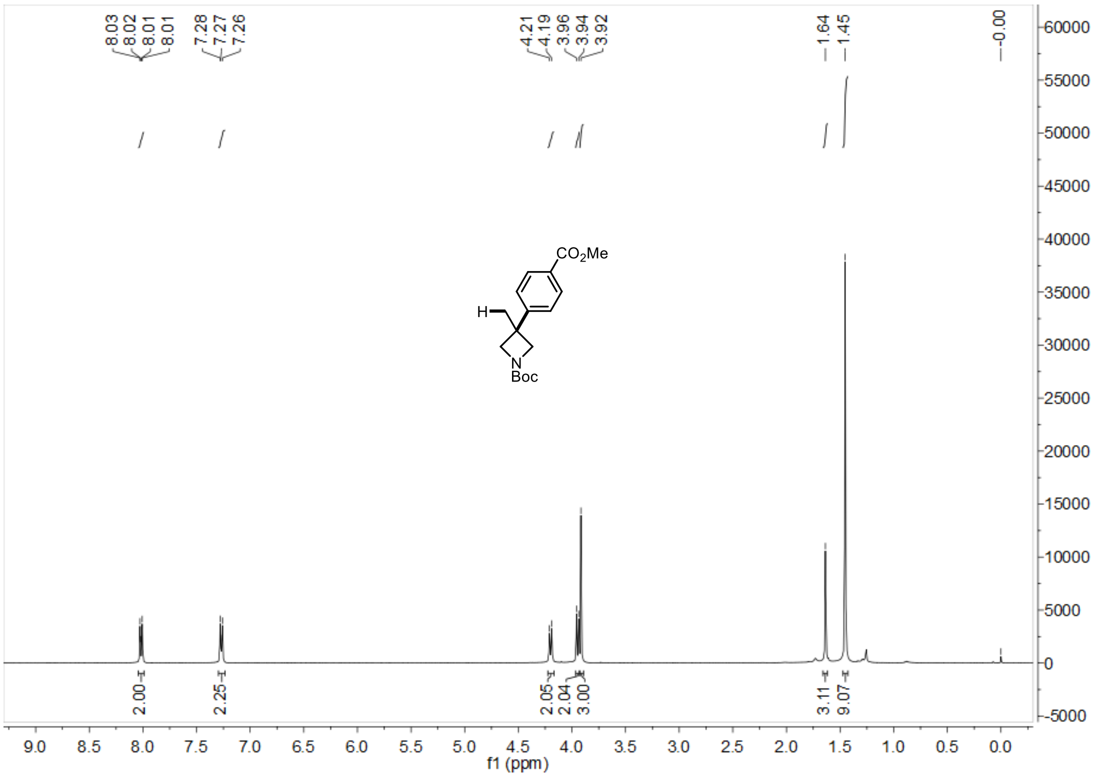

Compound 3ar ^{13}C NMR (101 MHz, $CDCl_3$)

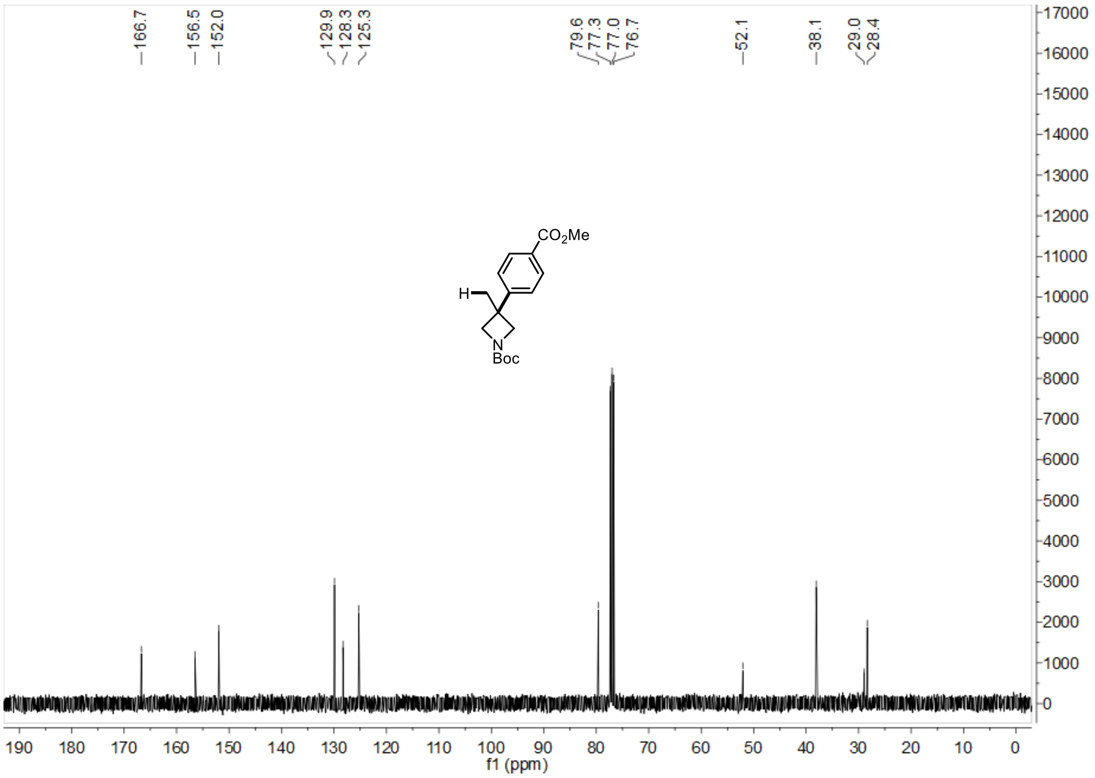

Compound 3bg ^{1}H NMR (400 MHz, $CDCl_3$)

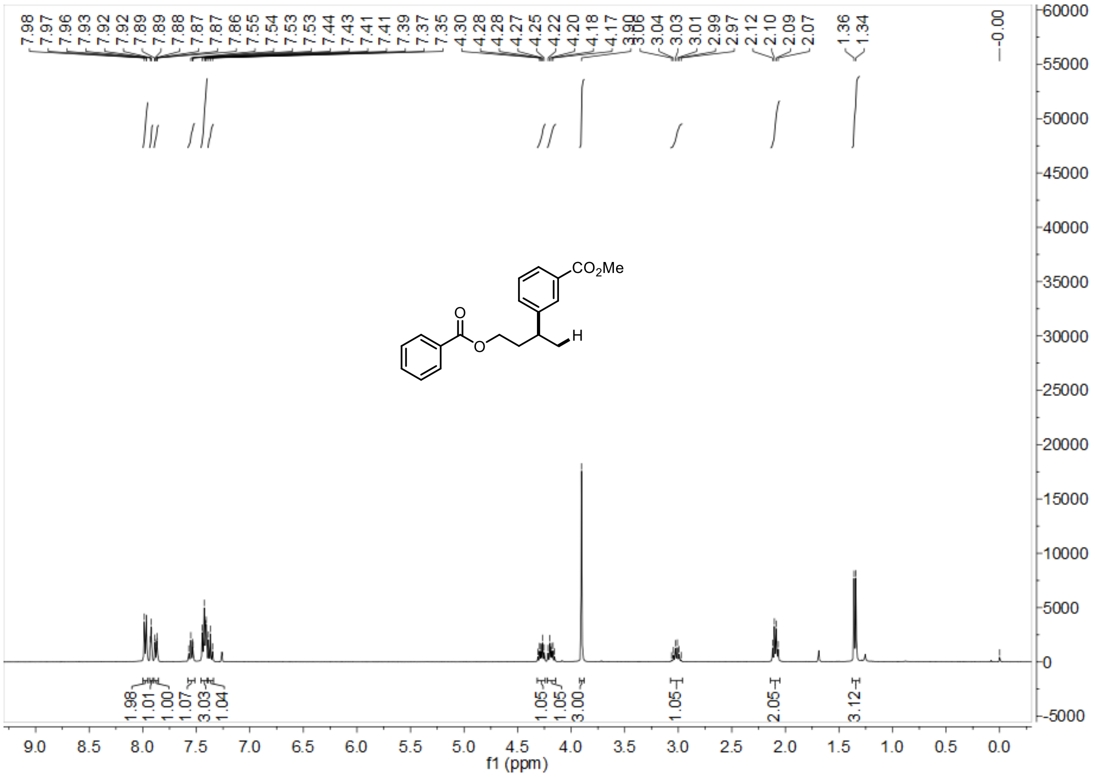

Compound 3bg ^{13}C NMR (101 MHz, $CDCl_3$)

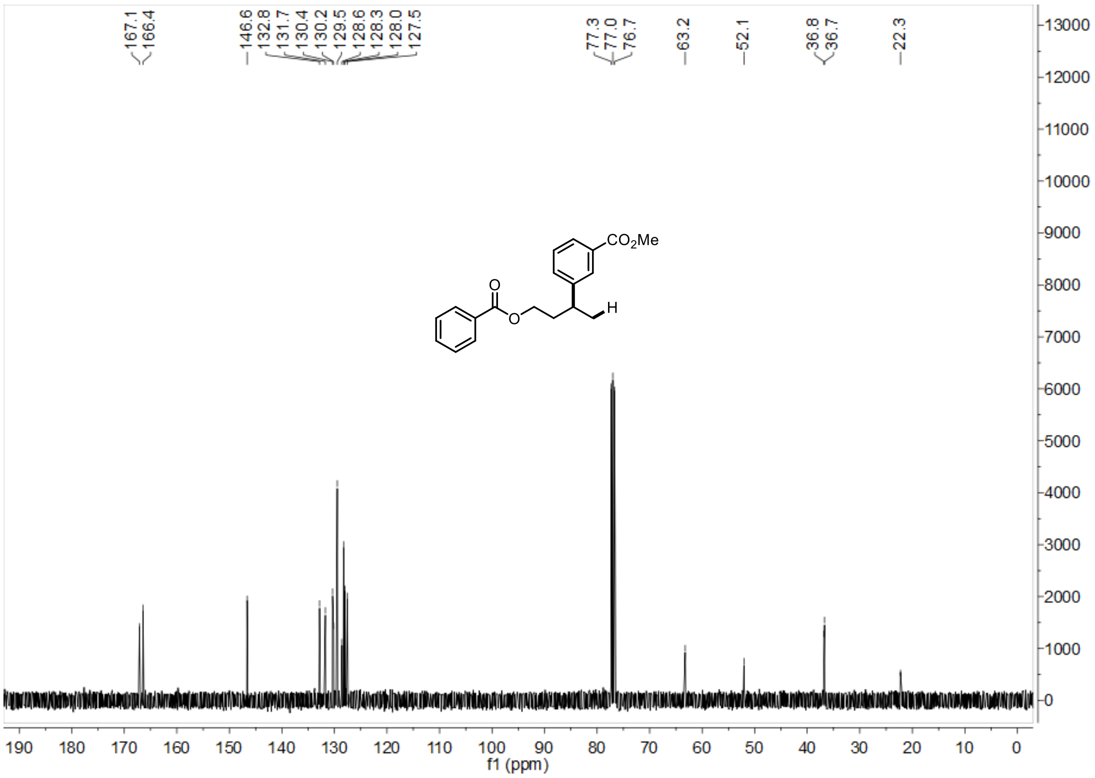

Compound 3cg ¹H NMR (400 MHz, CDCl₃)

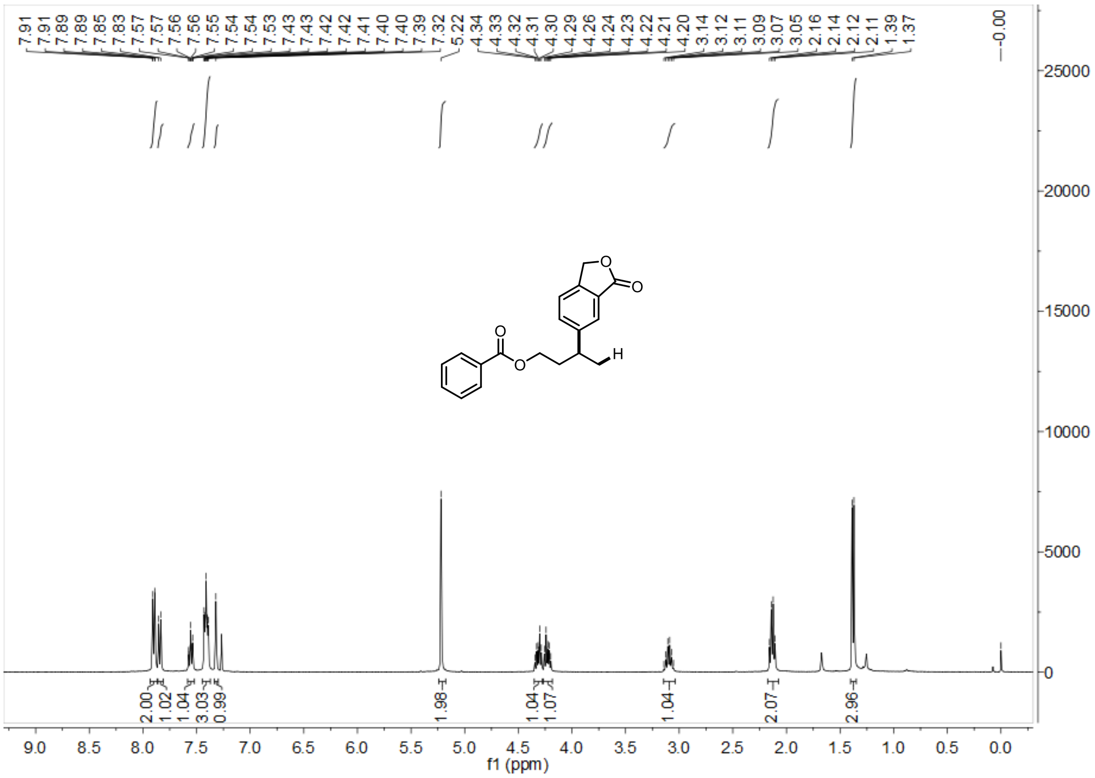

Compound 3cg ¹³C NMR (101 MHz, CDCl₃)

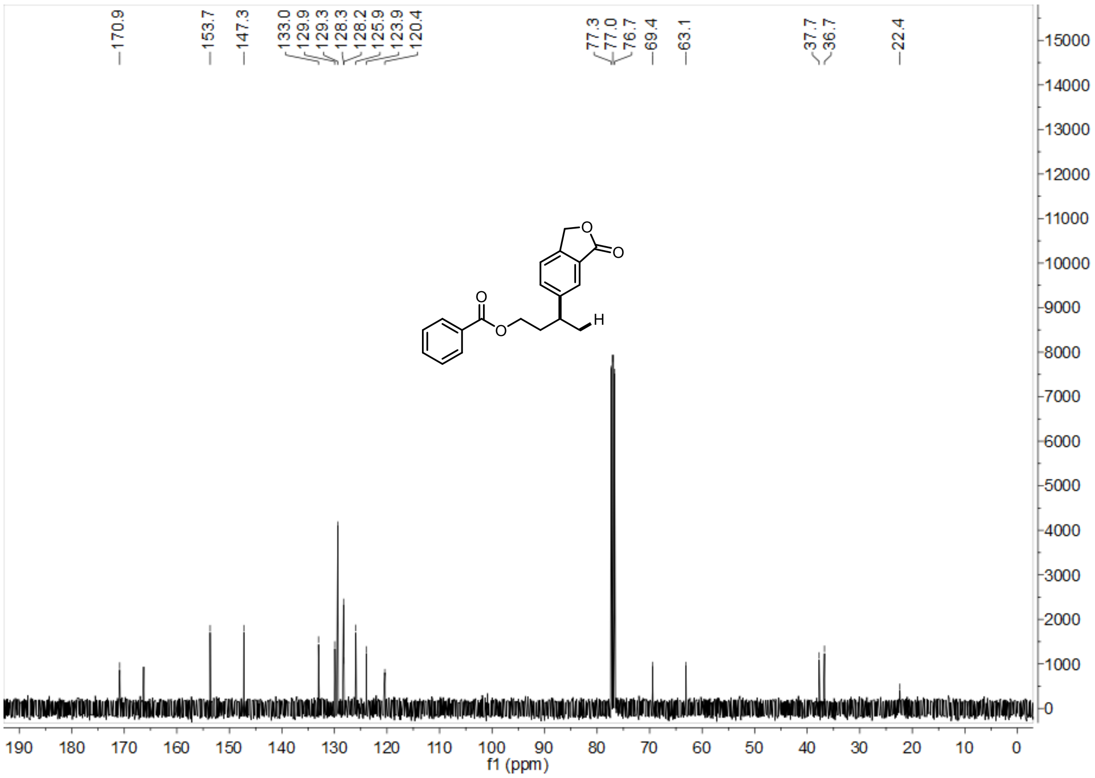

Compound 3dg ^1H NMR (400 MHz, $CDCl_3$)

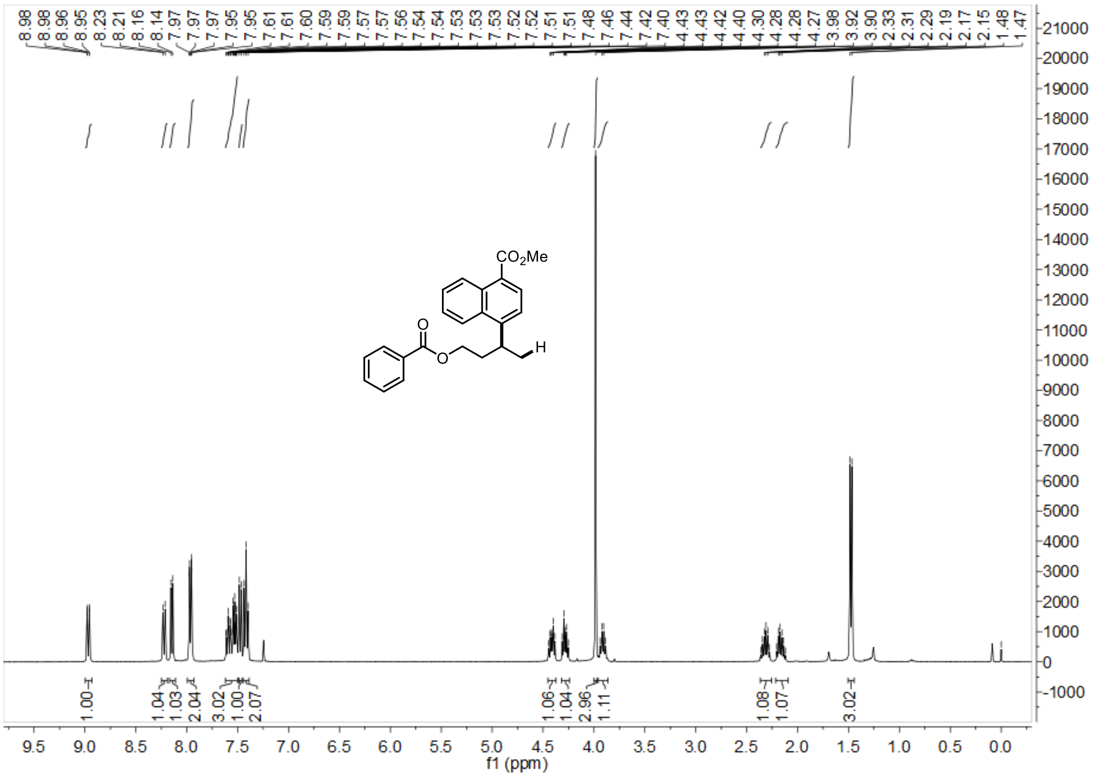

Compound 3dg ^{13}C NMR (101 MHz, $CDCl_3$)

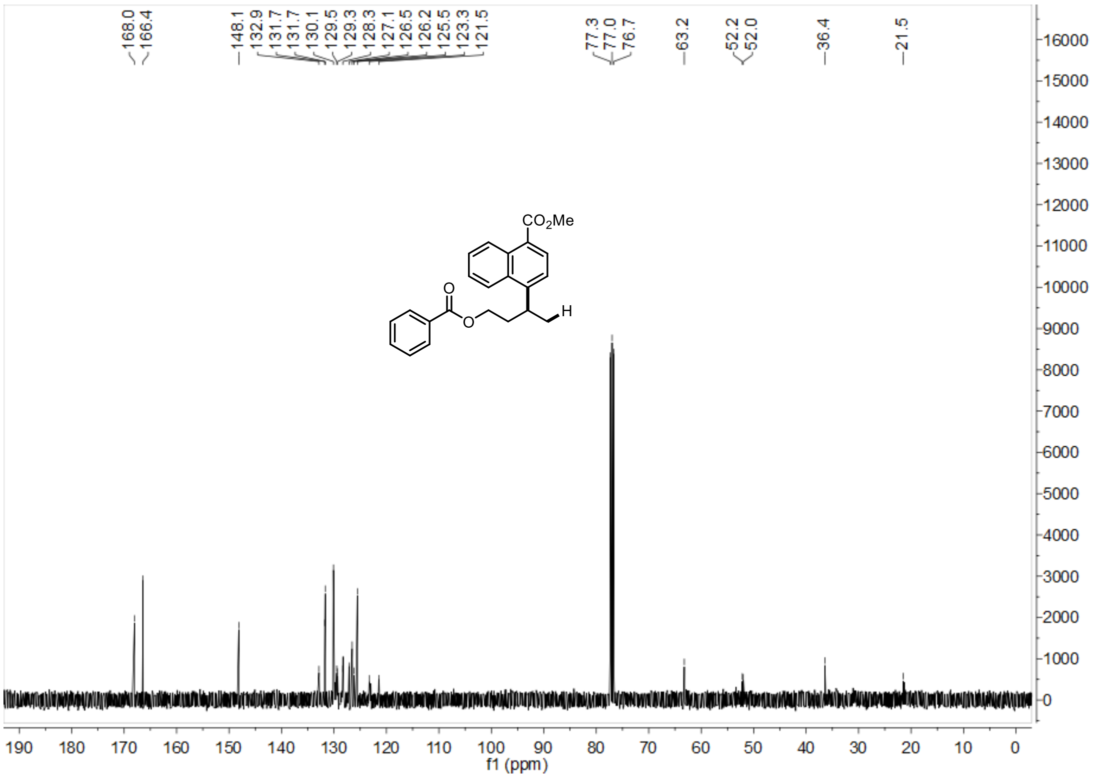

Compound 3eg ^{1}H NMR (400 MHz, CDCl$_3$)

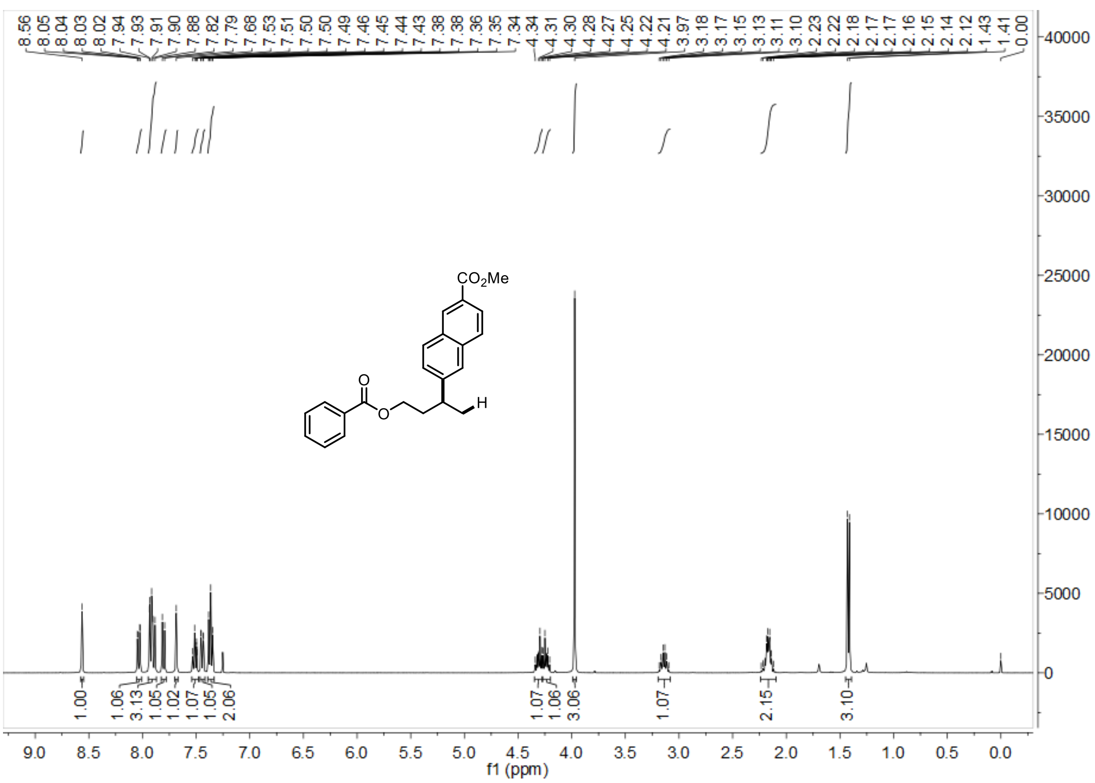

Compound 3eg ^{13}C NMR (101 MHz, CDCl$_3$)

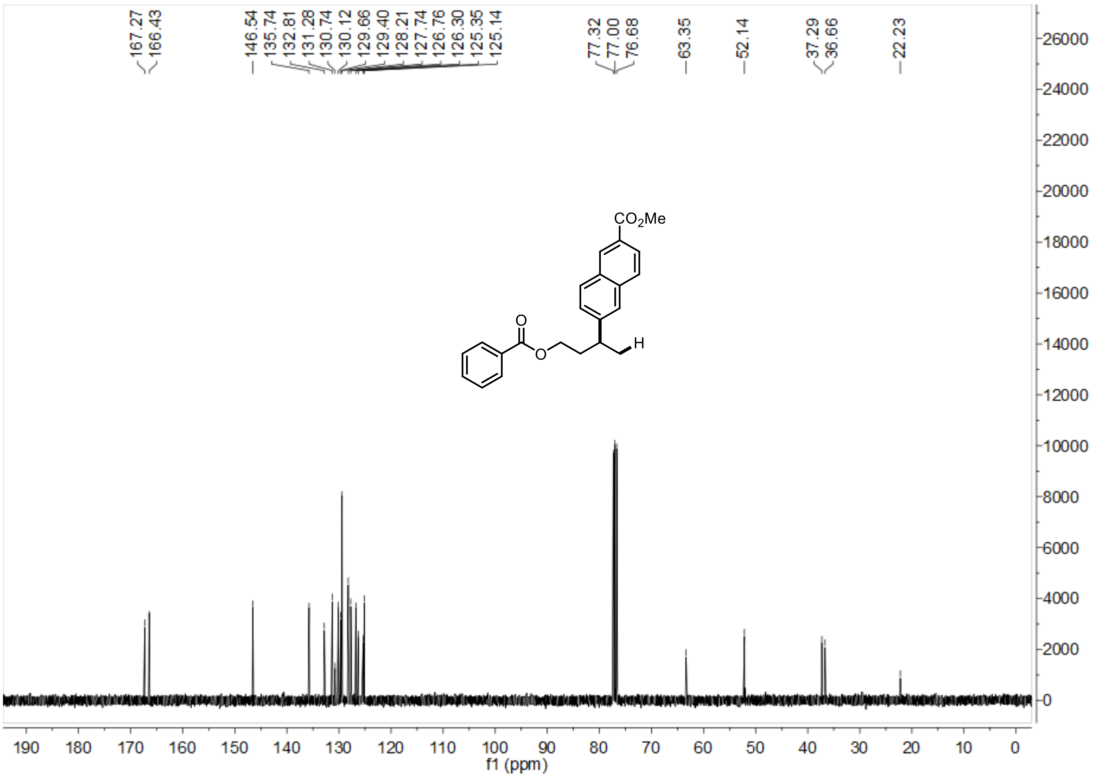

Compound 3fg ^{1}H NMR (400 MHz, $CDCl_3$)

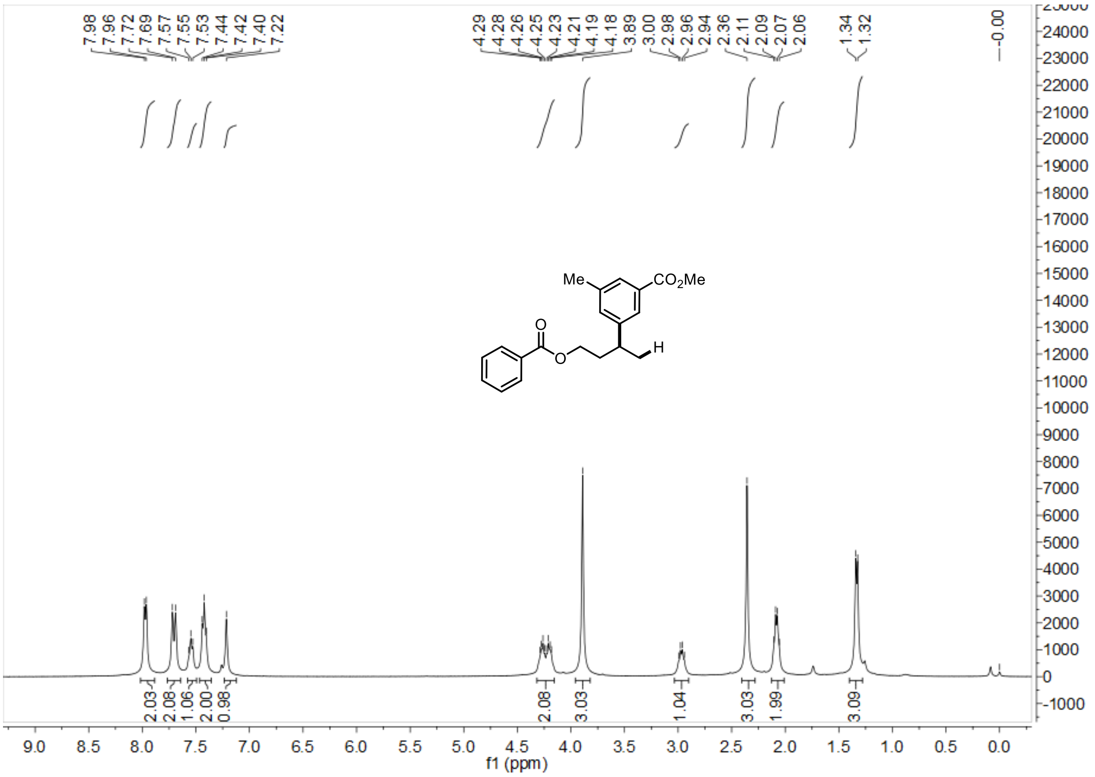

Compound 3fg ^{13}C NMR (101 MHz, $CDCl_3$)

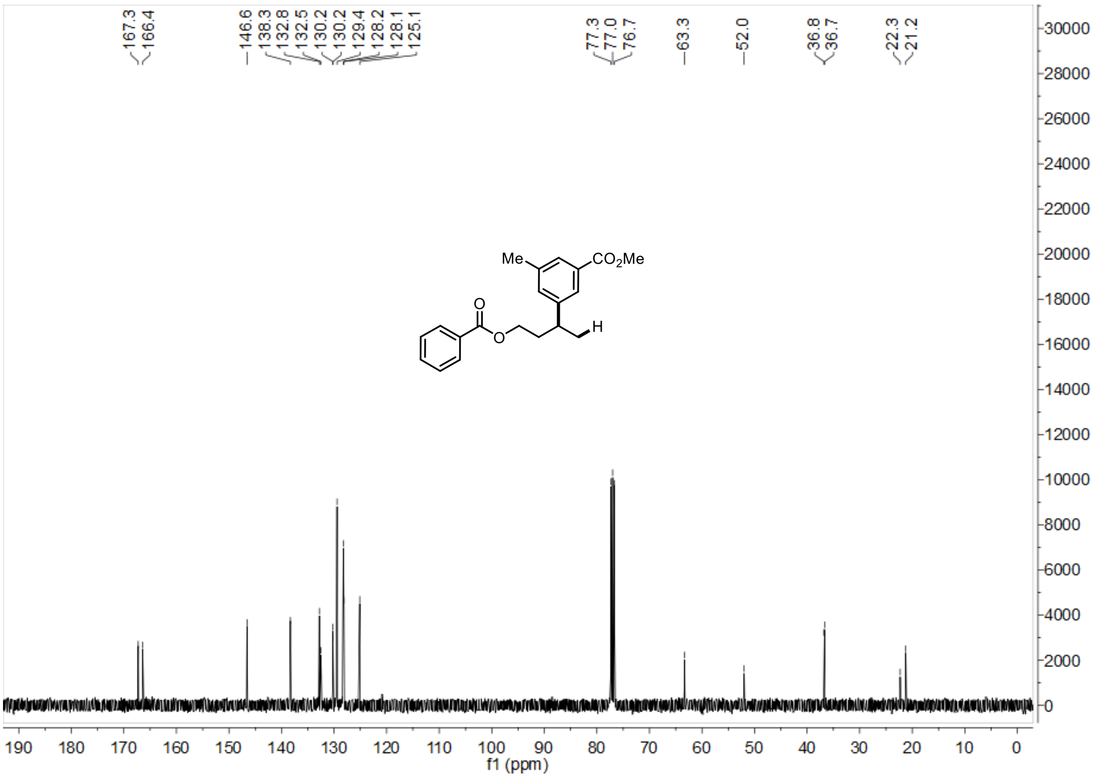

Compound 3gg ^{1}H NMR (400 MHz, $CDCl_3$)

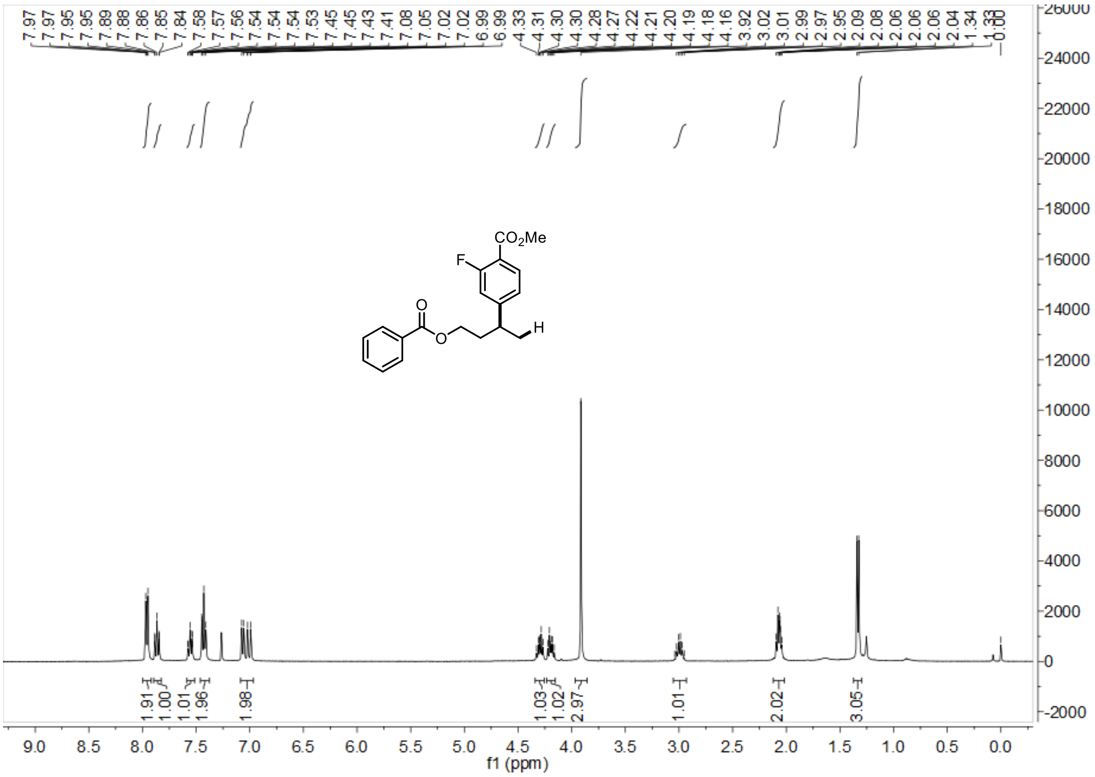

Compound 3gg ^{13}C NMR (101 MHz, $CDCl_3$)

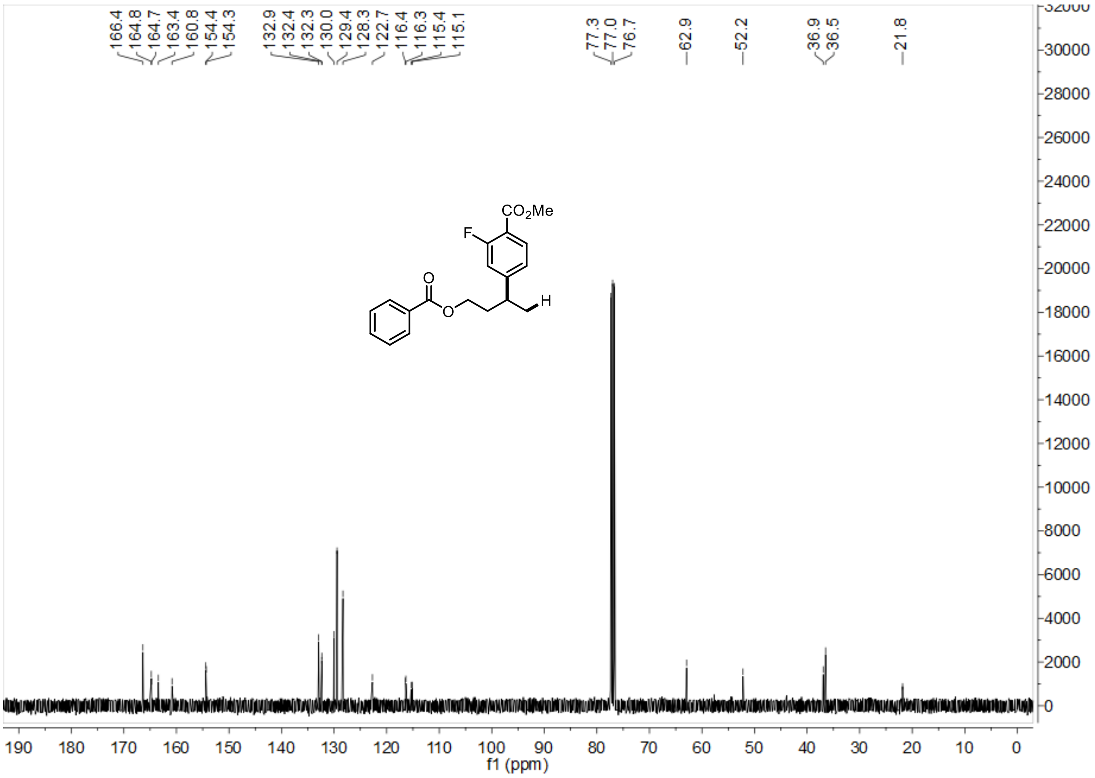

Compound 3gg ^{19}F NMR (376 MHz, $CDCl_3$)

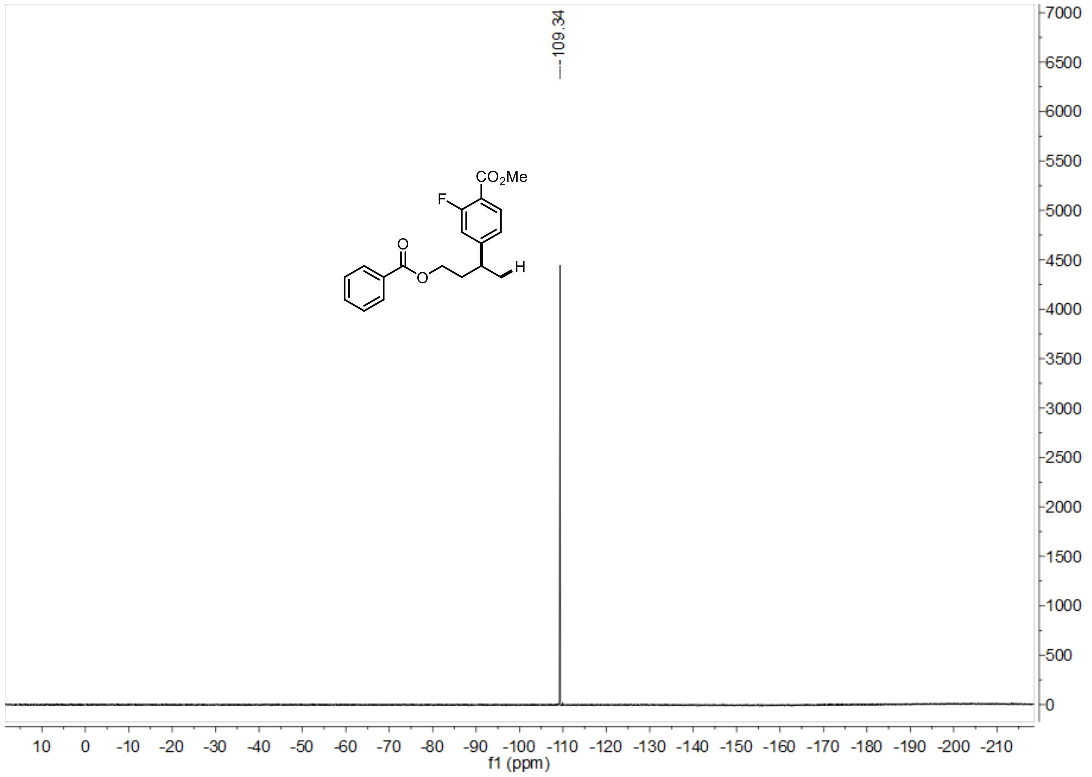

Compound 3hg ^{1}H NMR (400 MHz, $CDCl_3$)

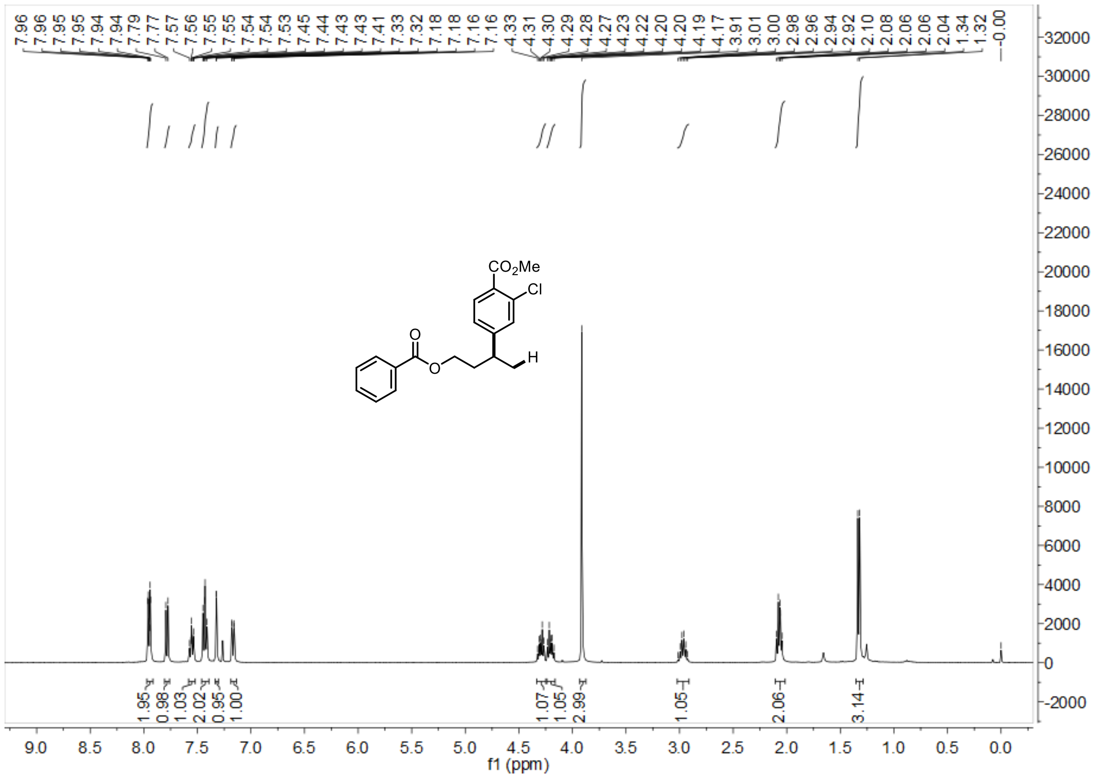

Compound 3hg ^{13}C NMR (101 MHz, $CDCl_3$)

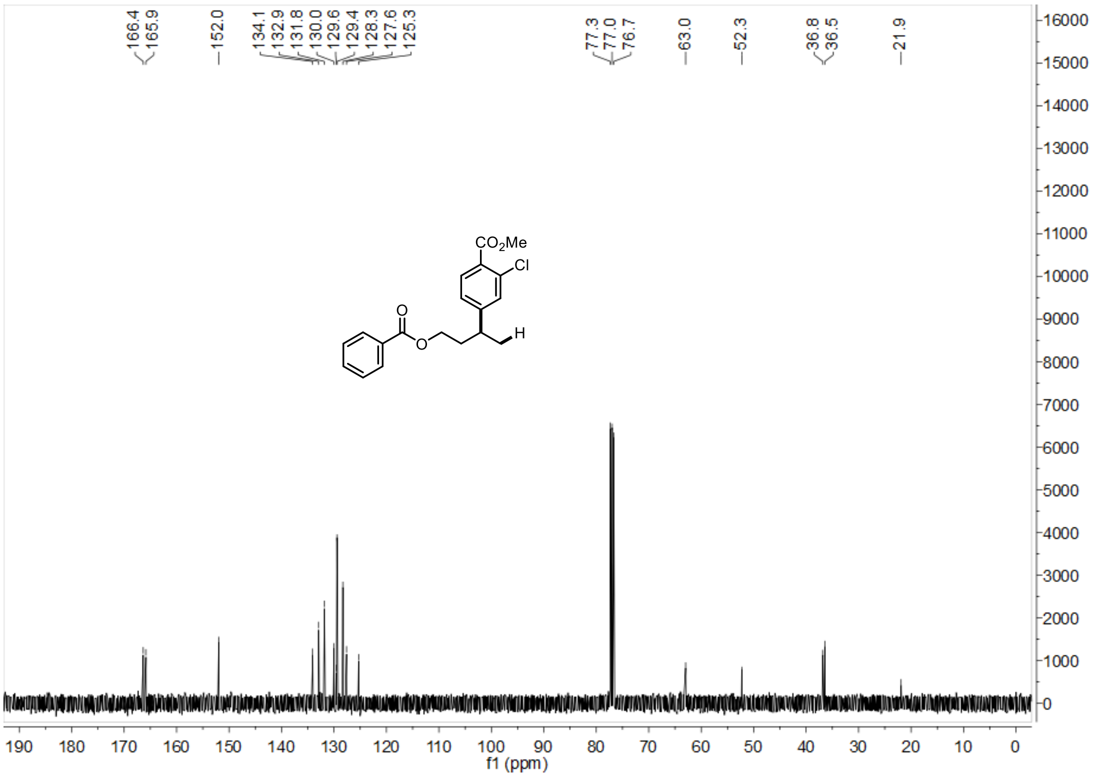

Compound 3ig ^{1}H NMR (400 MHz, $CDCl_3$)

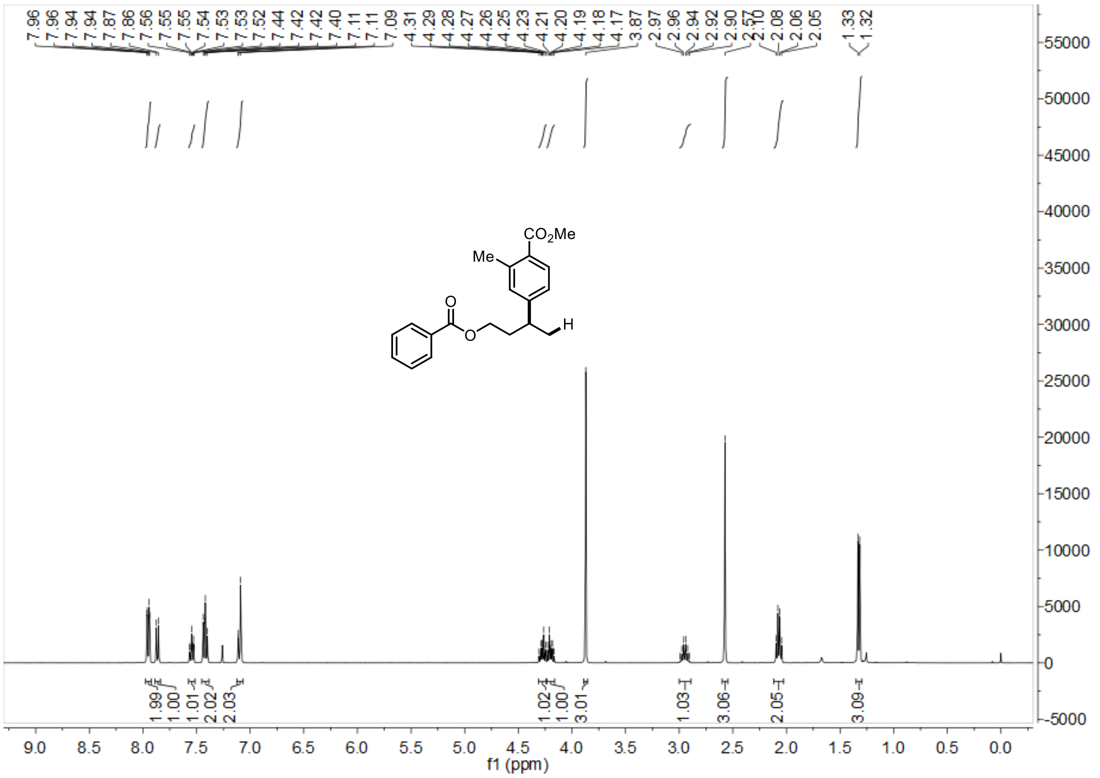

Compound 3ig ^{13}C NMR (101 MHz, $CDCl_3$)

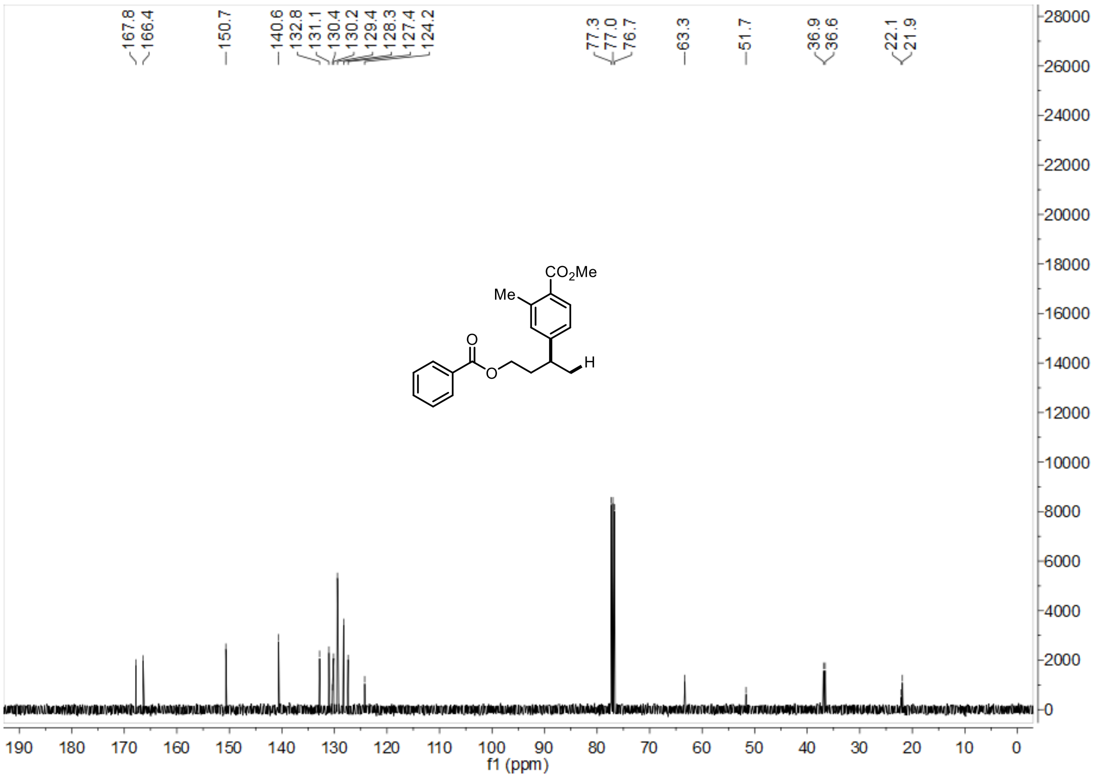

Compound 3jg ^{1}H NMR (400 MHz, $CDCl_3$)

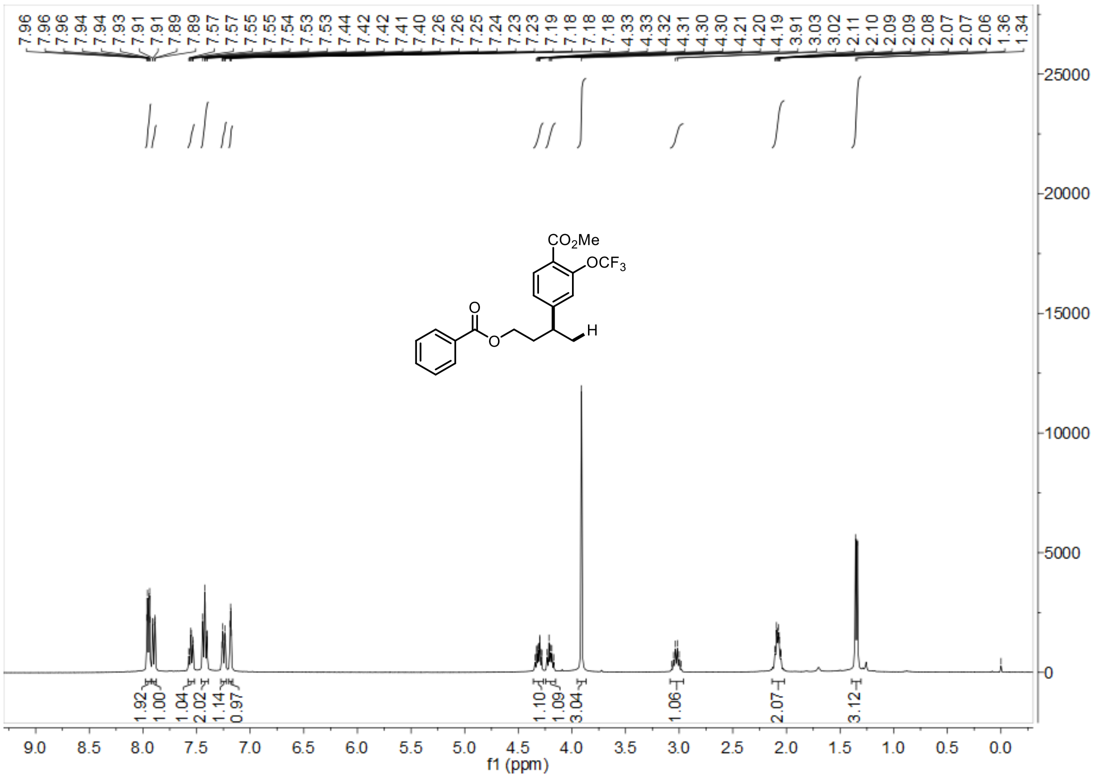

Compound 3jg ^{13}C NMR (101 MHz, $CDCl_3$)

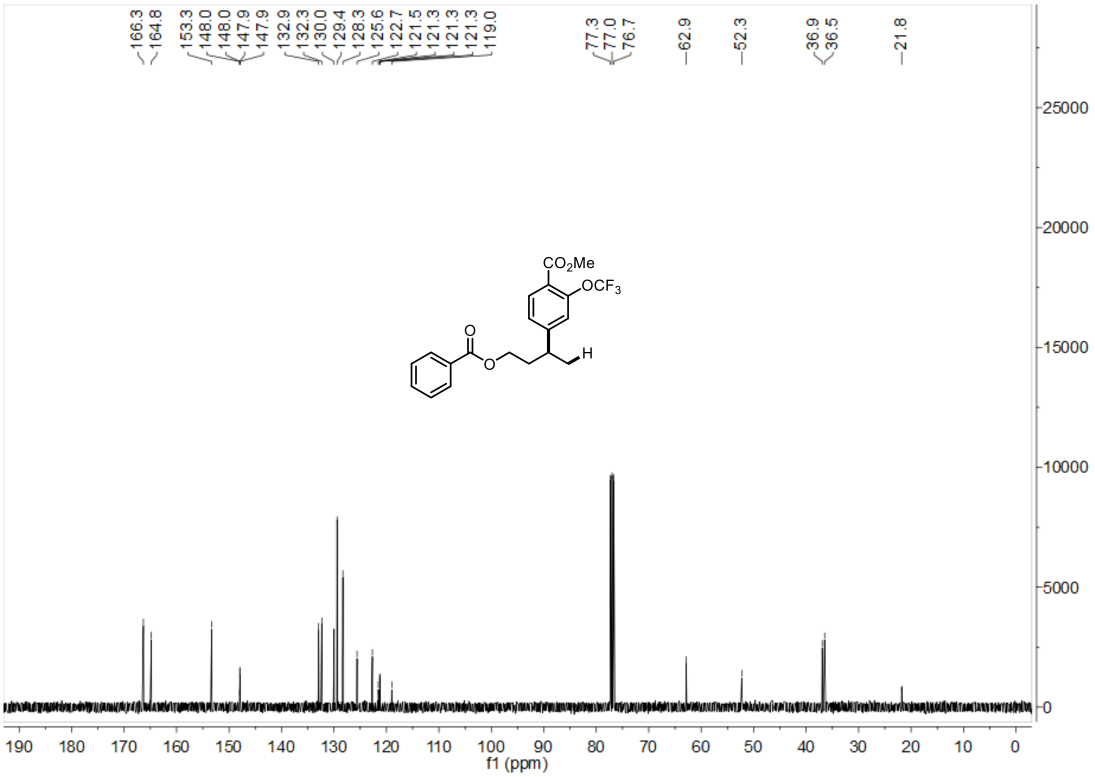

Compound 3jg ^{19}F NMR (376 MHz, $CDCl_3$)

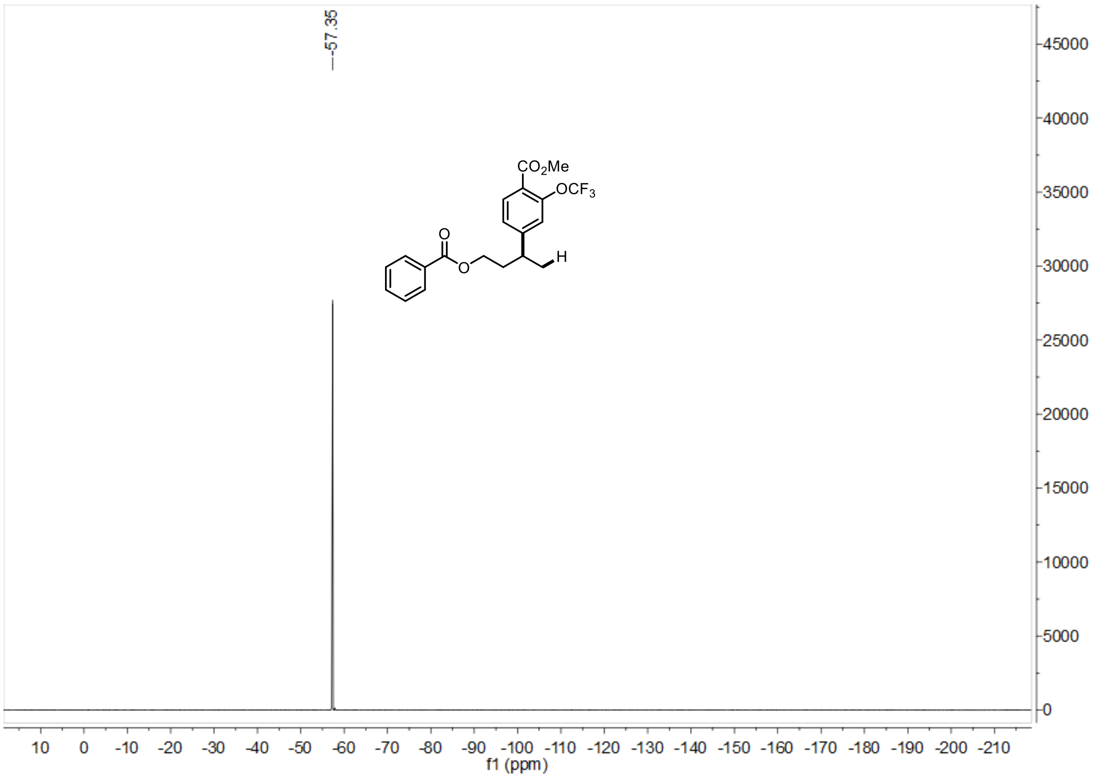

Compound 3kg ^{1}H NMR (400 MHz, $CDCl_3$)

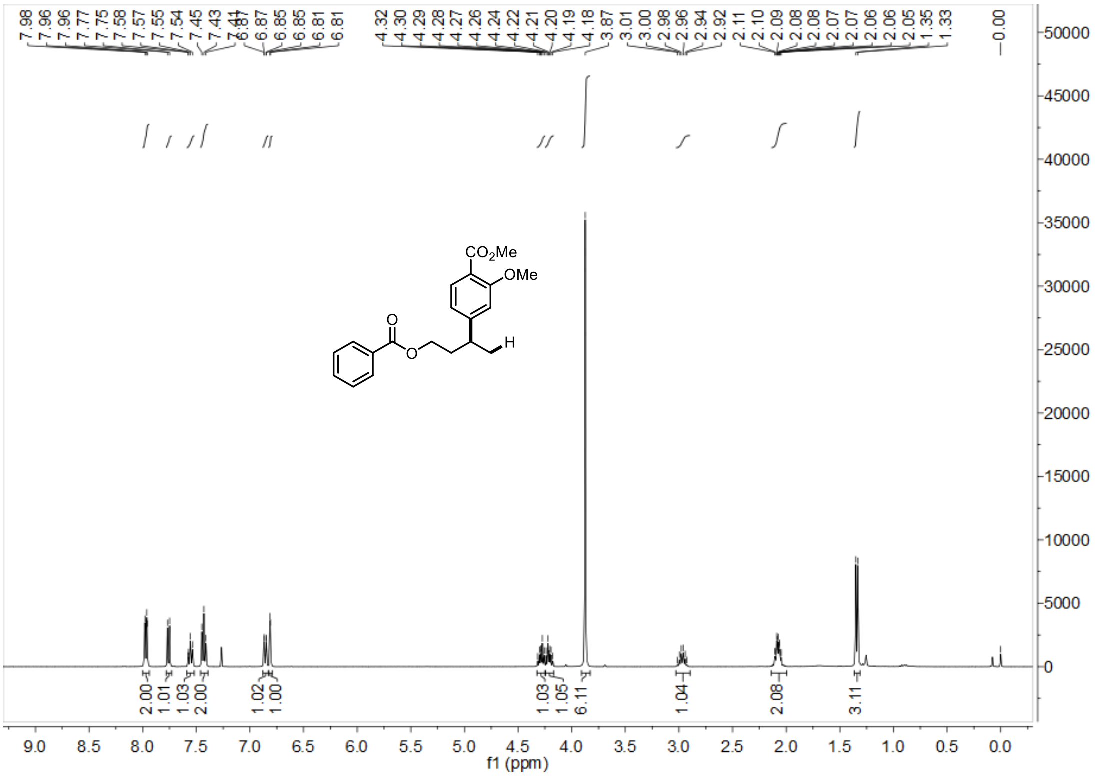

Compound 3kg ^{13}C NMR (101 MHz, $CDCl_3$)

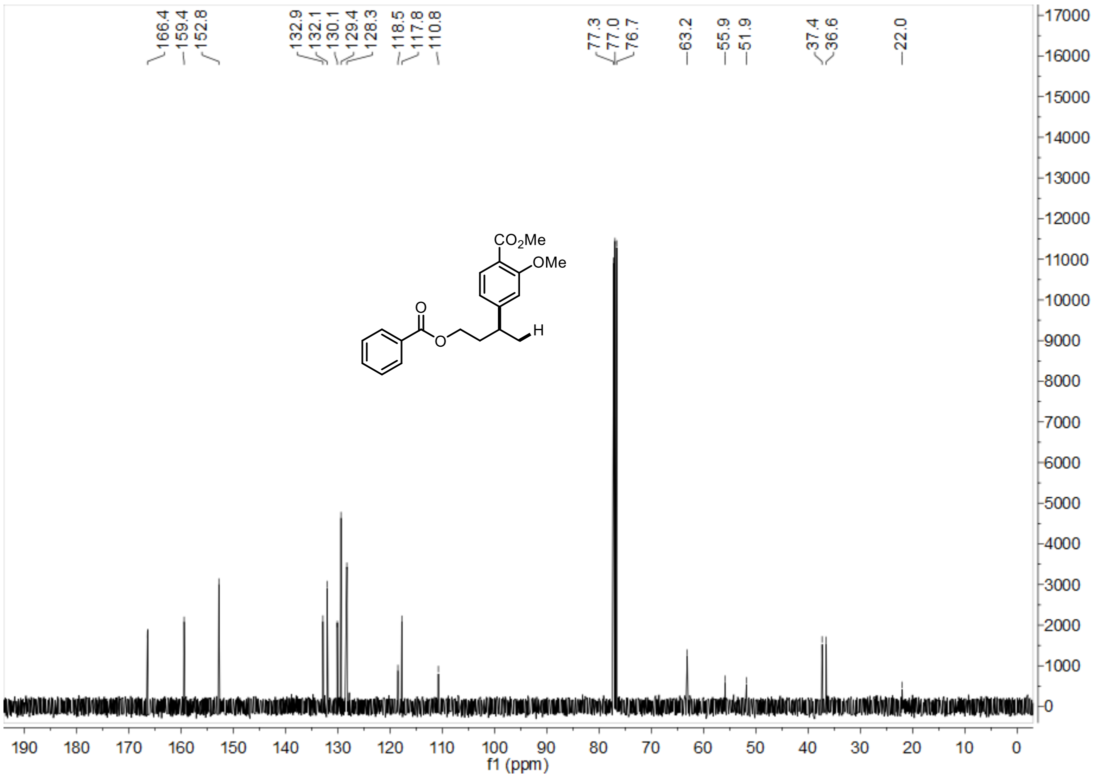

Compound 3lg ^{1}H NMR (400 MHz, $CDCl_3$)

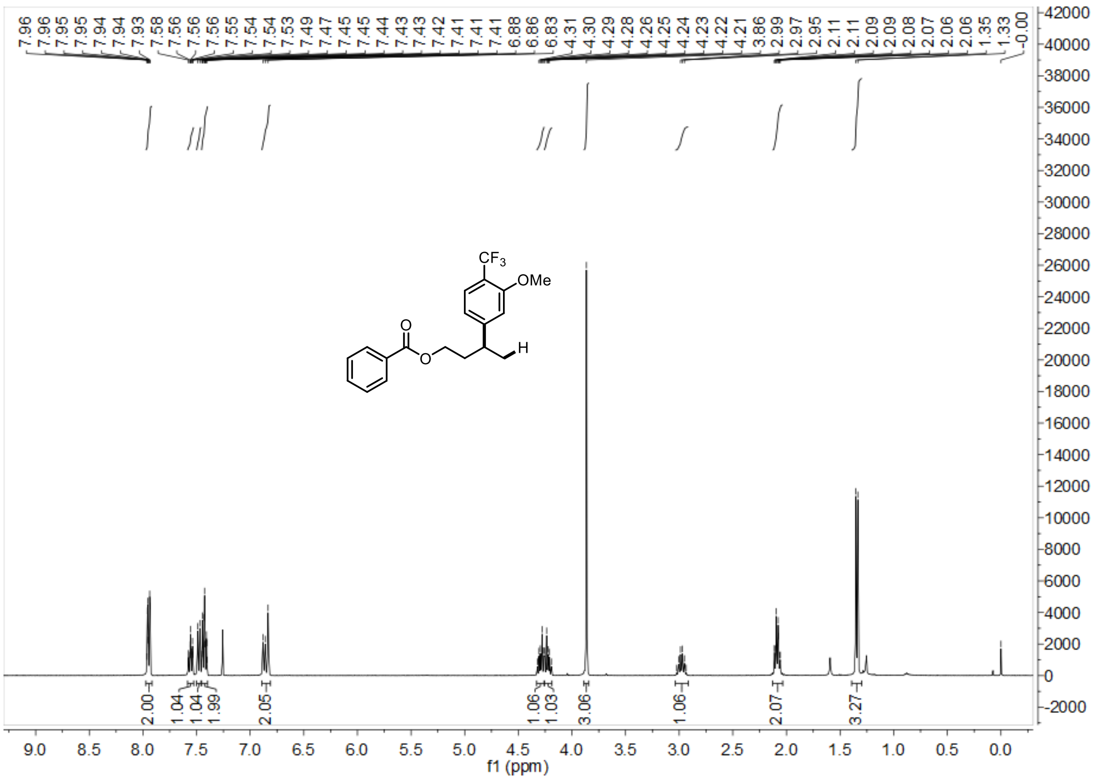

Compound 3lg ^{13}C NMR (101 MHz, $CDCl_3$)

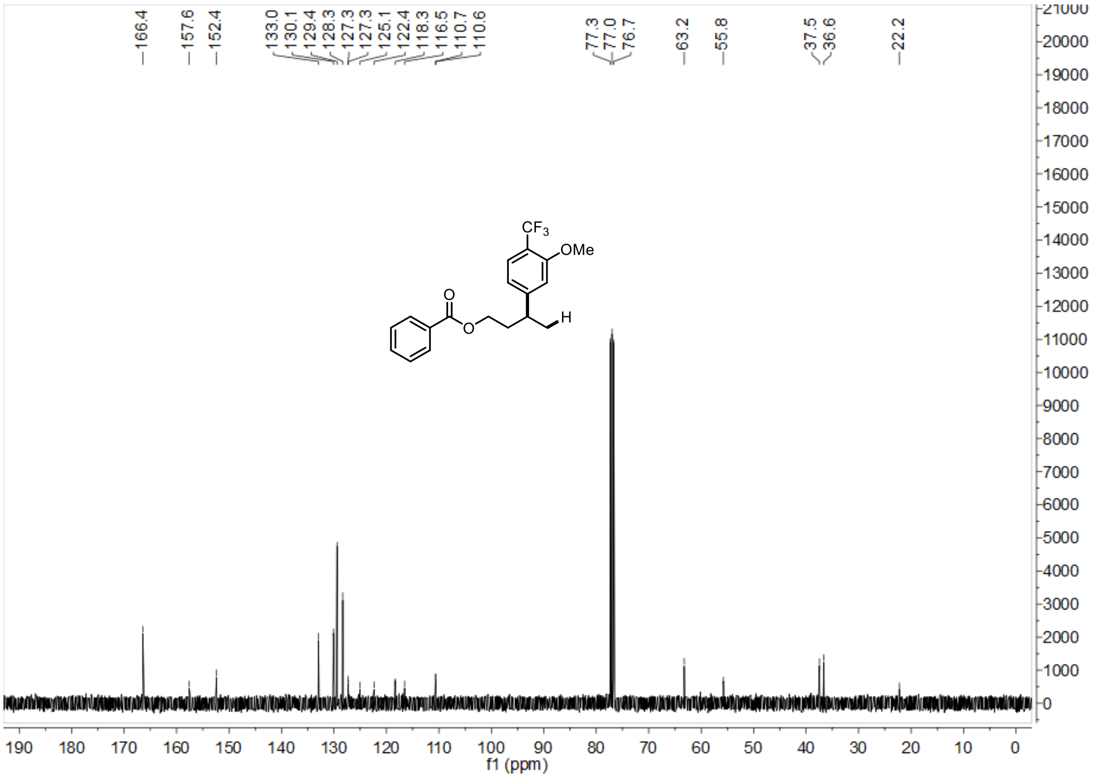

Compound 3lg ^{19}F NMR (376 MHz, $CDCl_3$)

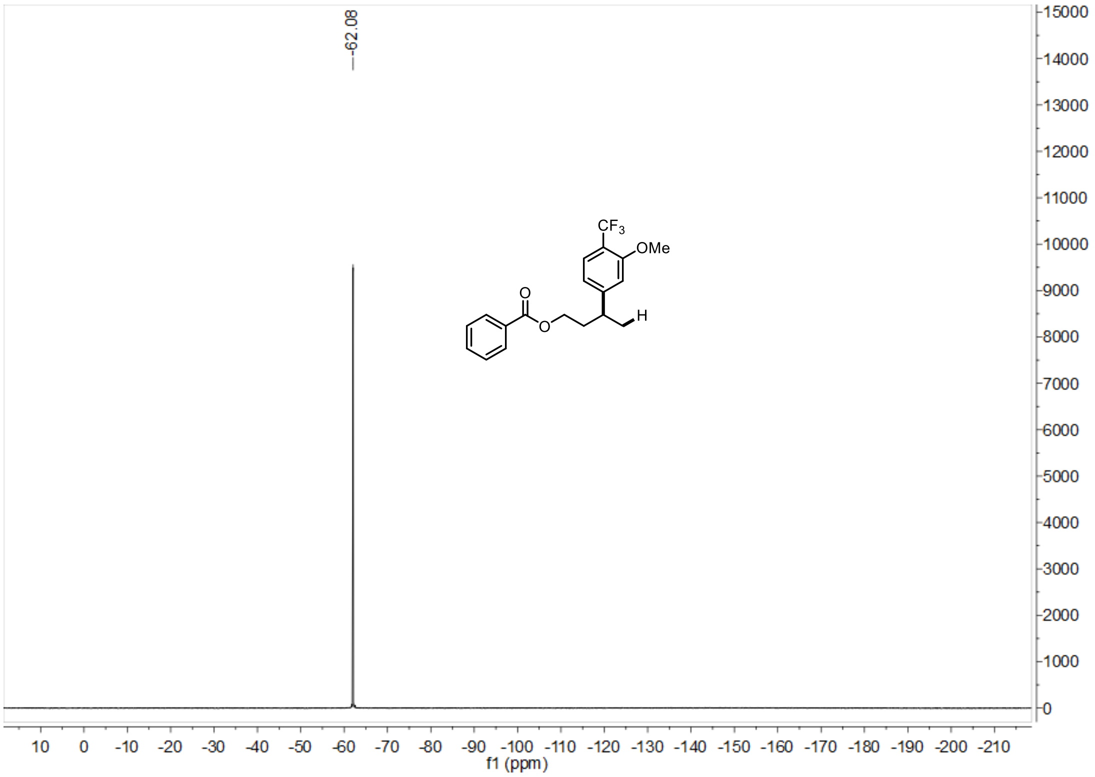

Compound 3mg ^{1}H NMR (400 MHz, $CDCl_3$)

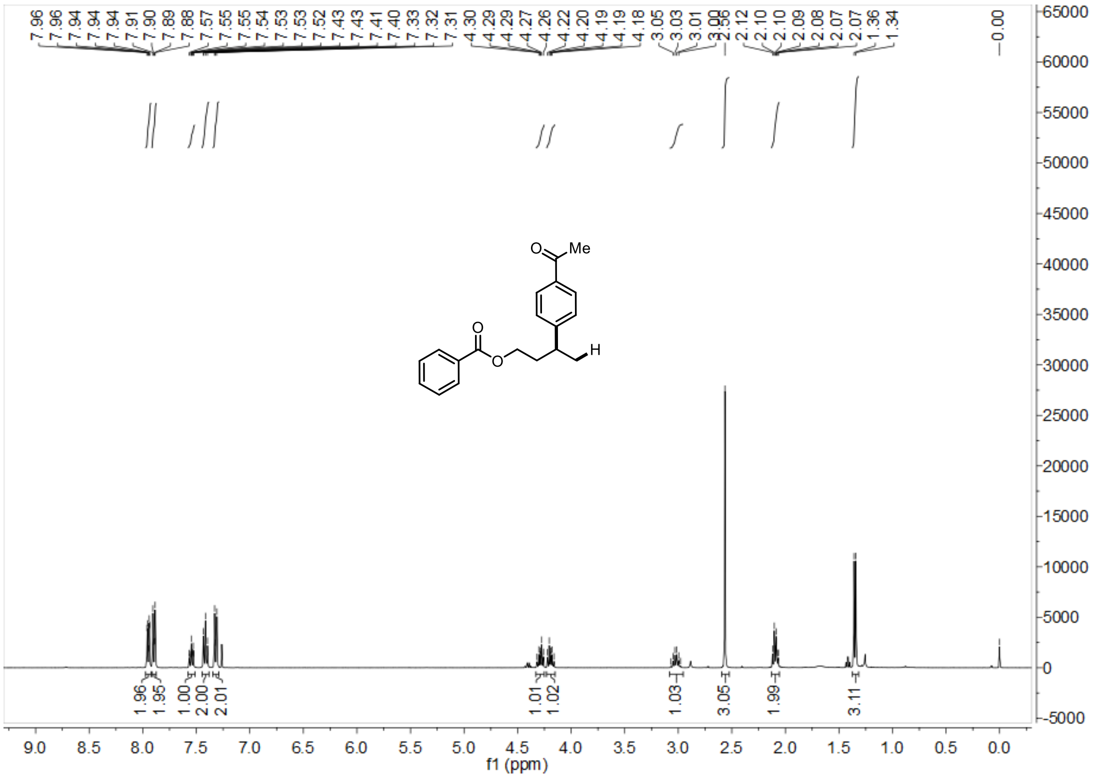

Compound 3mg ^{13}C NMR (101 MHz, $CDCl_3$)

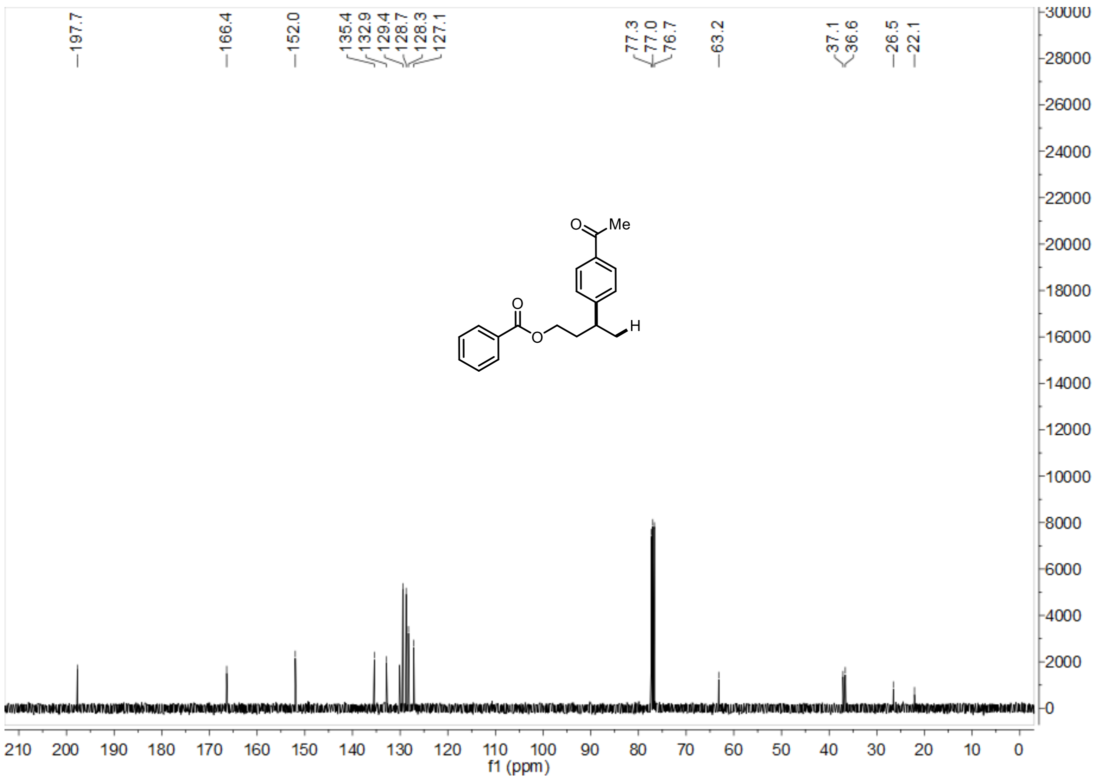

Compound 3ng ^{1}H NMR (400 MHz, $CDCl_3$)

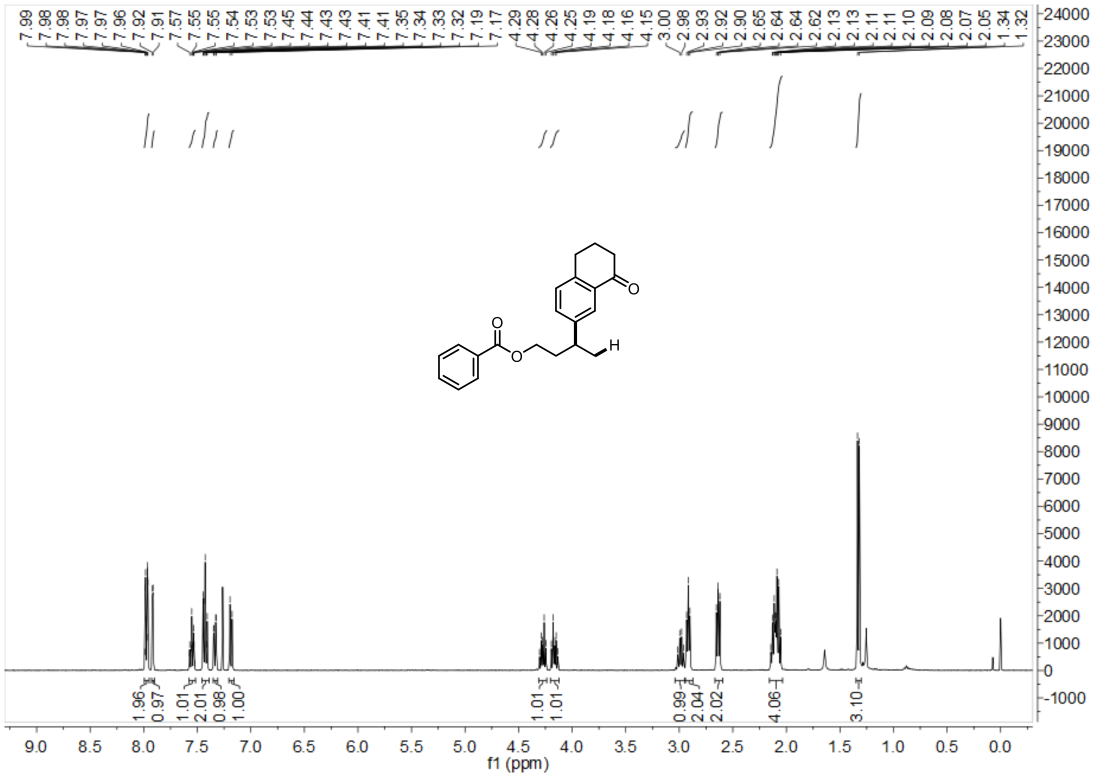

Compound 3ng ^{13}C NMR (101 MHz, $CDCl_3$)

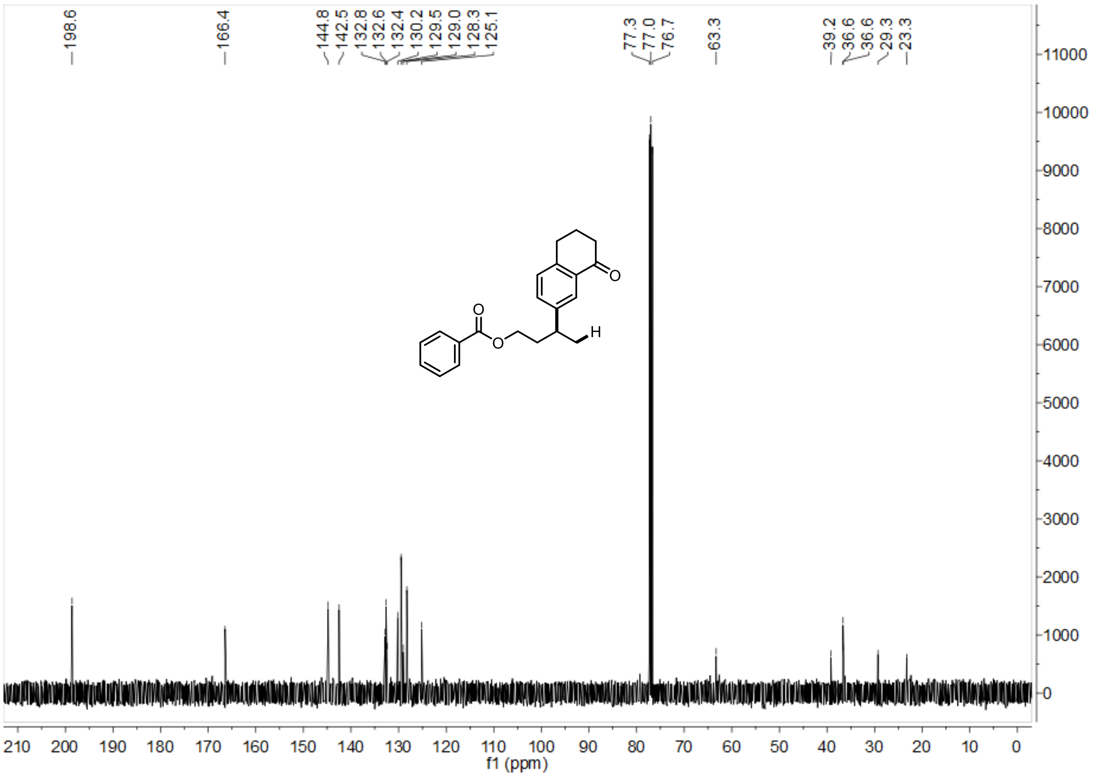

Compound 3og ^{1}H NMR (400 MHz, $CDCl_3$)

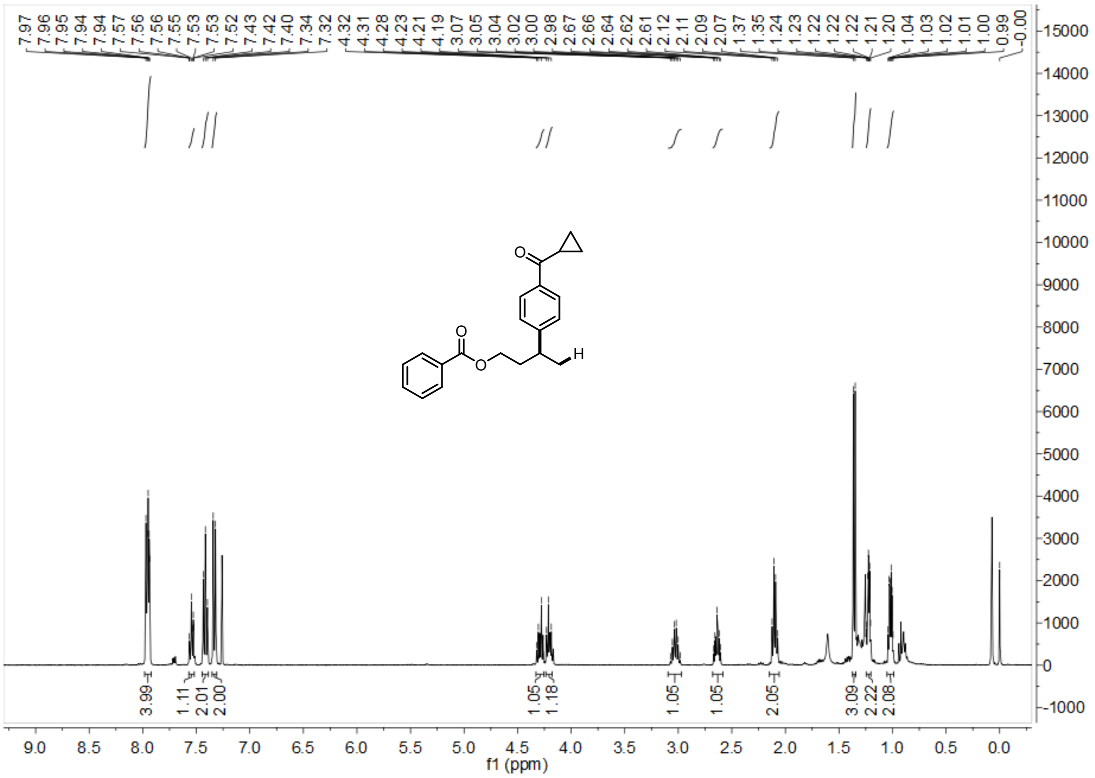

Compound 3og ^{13}C NMR (101 MHz, $CDCl_3$)

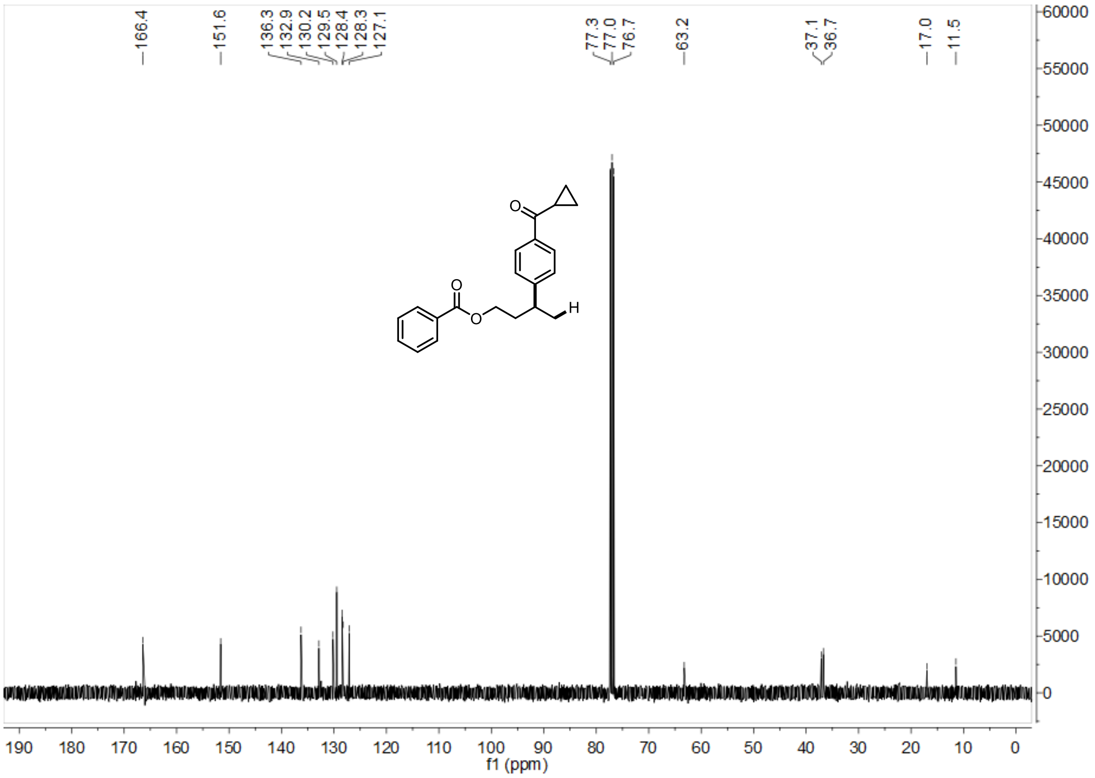

Compound 3pg ^{1}H NMR (400 MHz, $CDCl_3$)

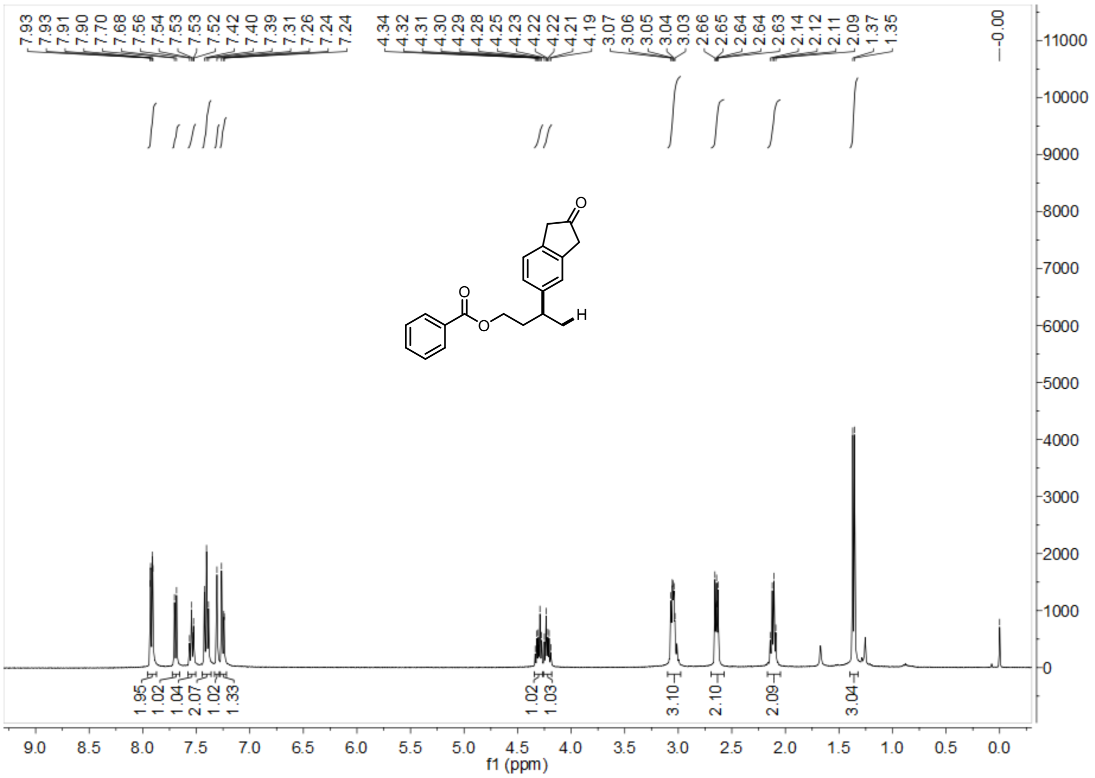

Compound 3pg ^{13}C NMR (101 MHz, $CDCl_3$)

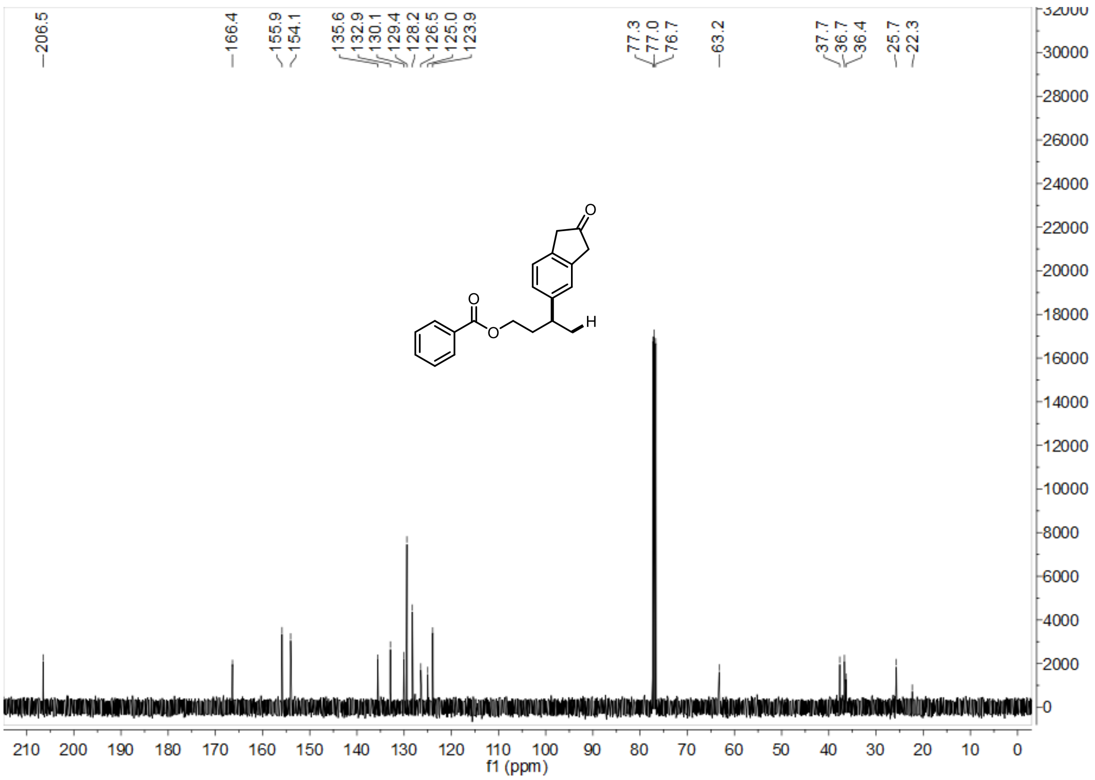

Compound 3qg ^{1}H NMR (400 MHz, $CDCl_3$)

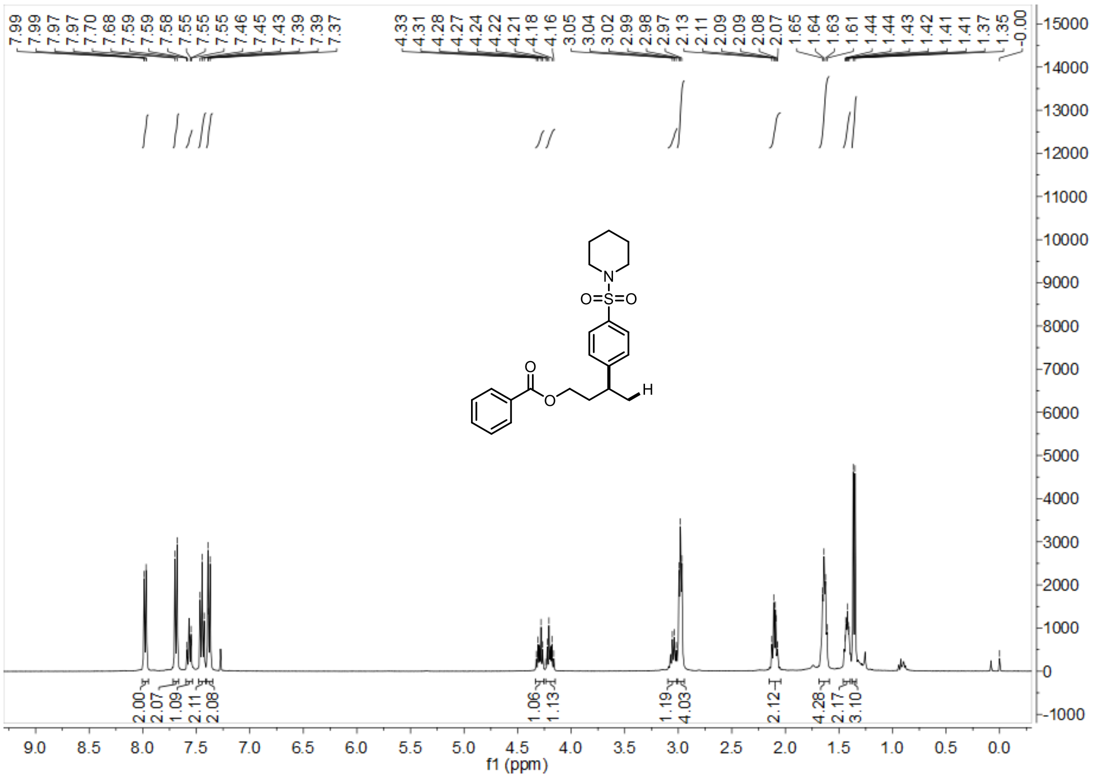

Compound 3qg ^{13}C NMR (101 MHz, $CDCl_3$)

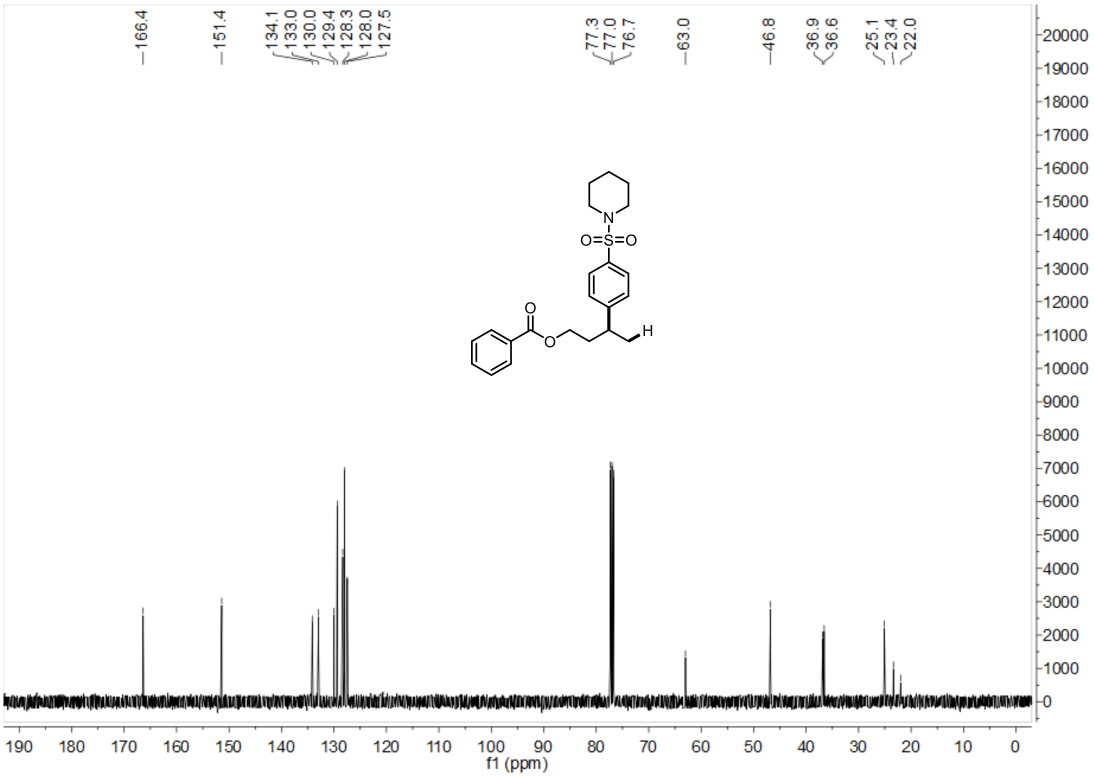

Compound 3rg ^{1}H NMR (400 MHz, $CDCl_3$)

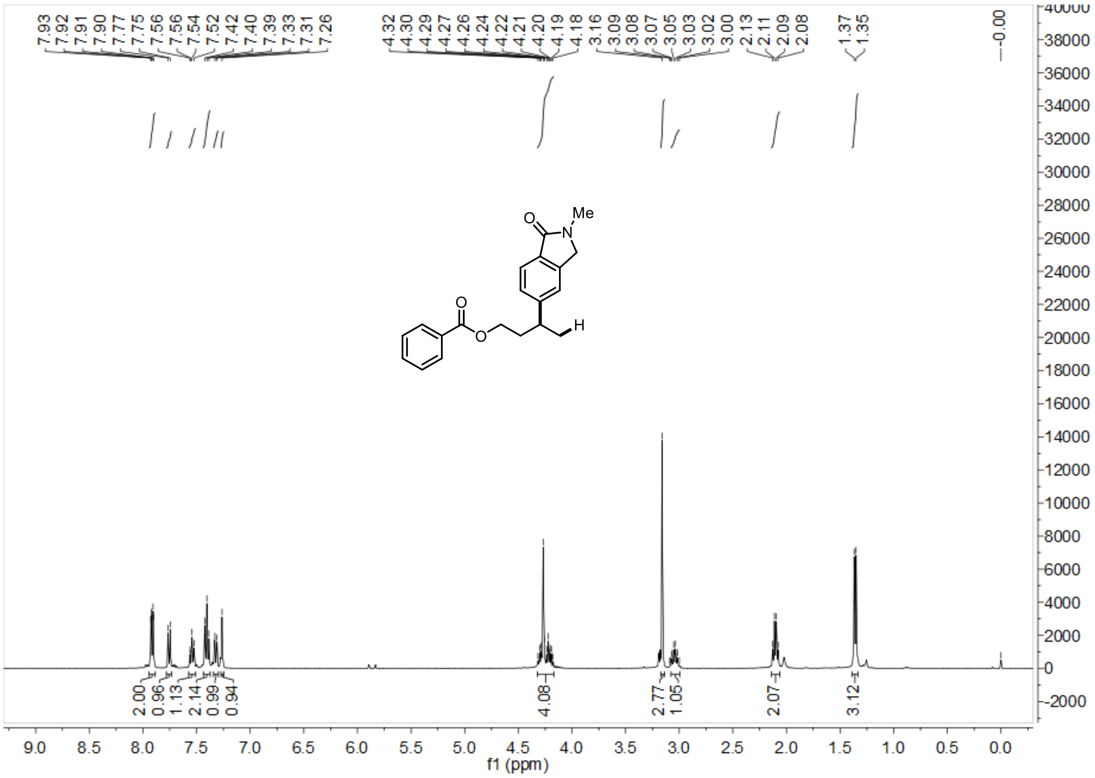

Compound 3rg ^{13}C NMR (101 MHz, $CDCl_3$)

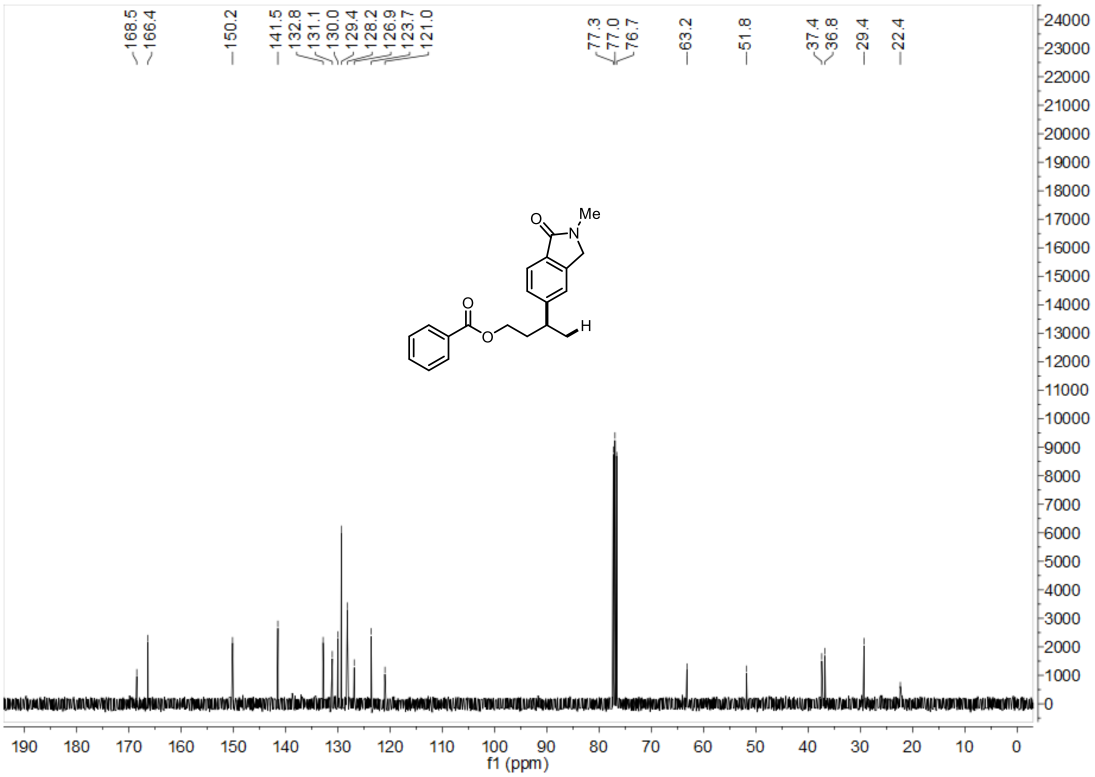

Compound 3sg ^{1}H NMR (400 MHz, $CDCl_3$)

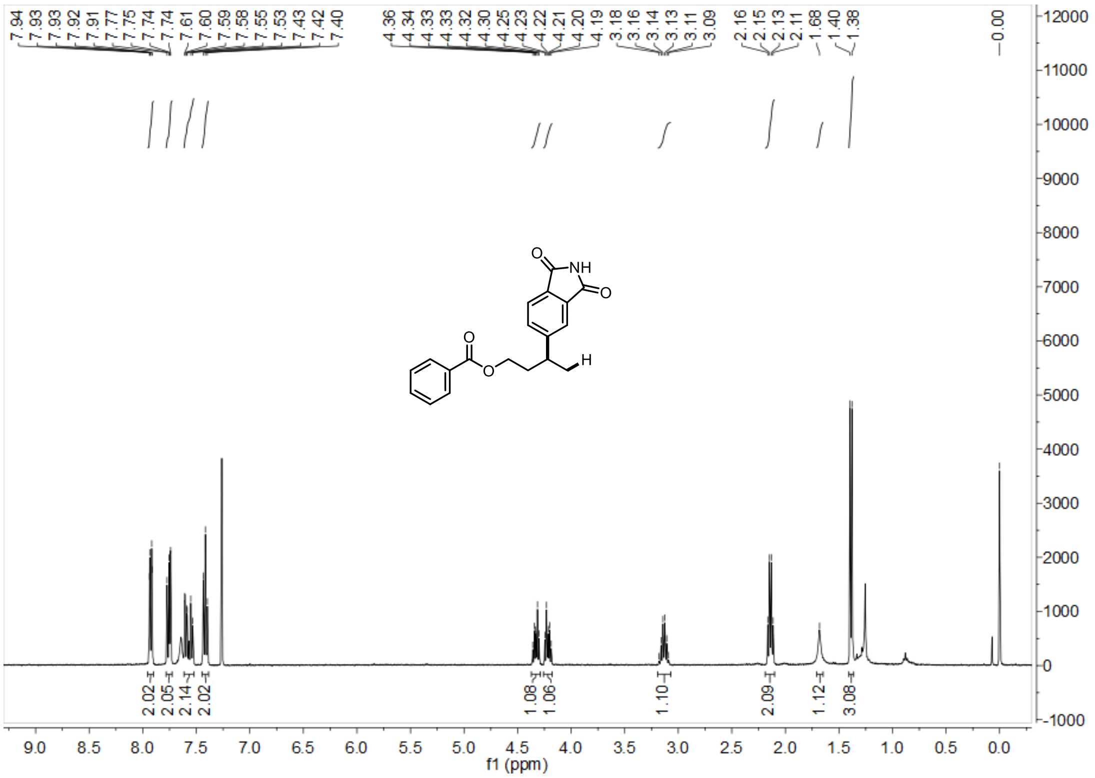

Compound 3sg ^{13}C NMR (101 MHz, $CDCl_3$)

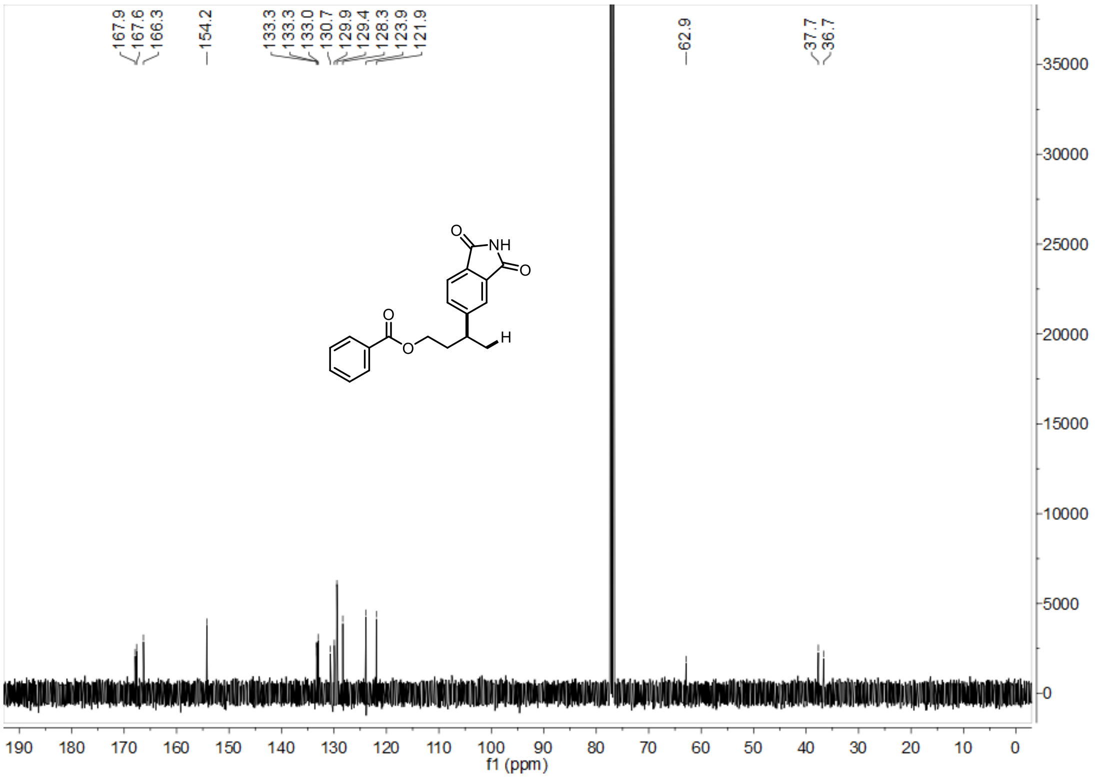

Compound 3tg ^{1}H NMR (400 MHz, $CDCl_3$)

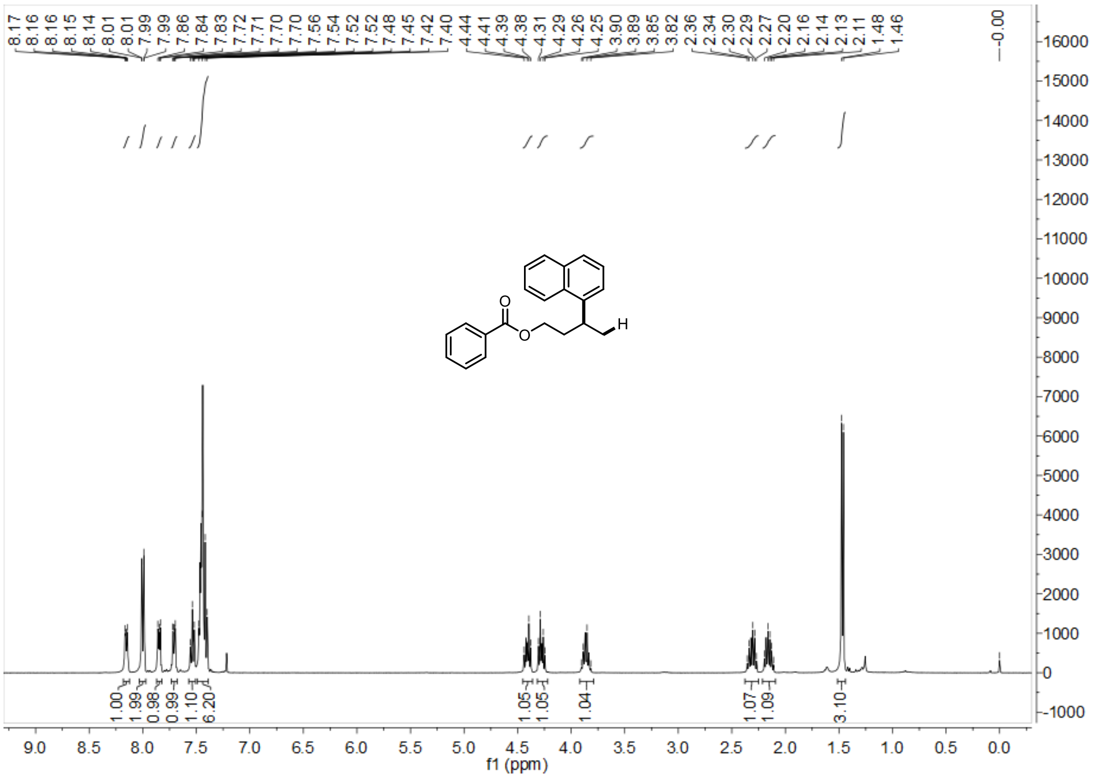

Compound 3tg ^{13}C NMR (101 MHz, $CDCl_3$)

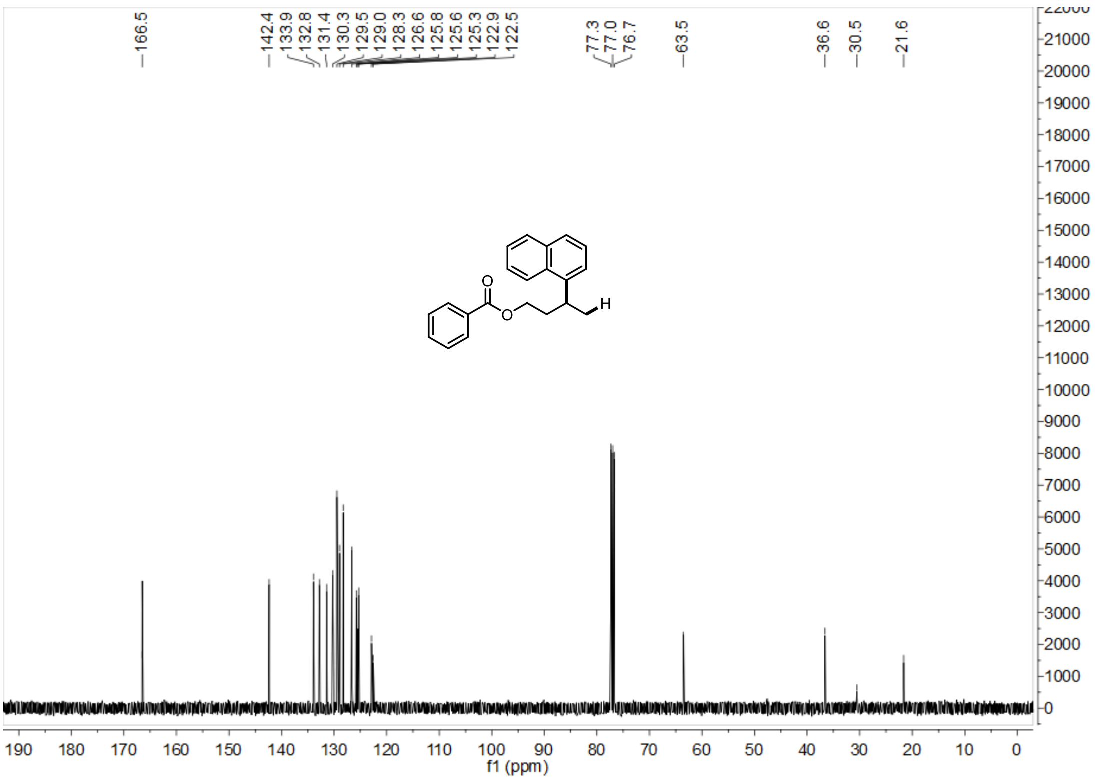

Compound 3ug ^{1}H NMR (400 MHz, $CDCl_3$)

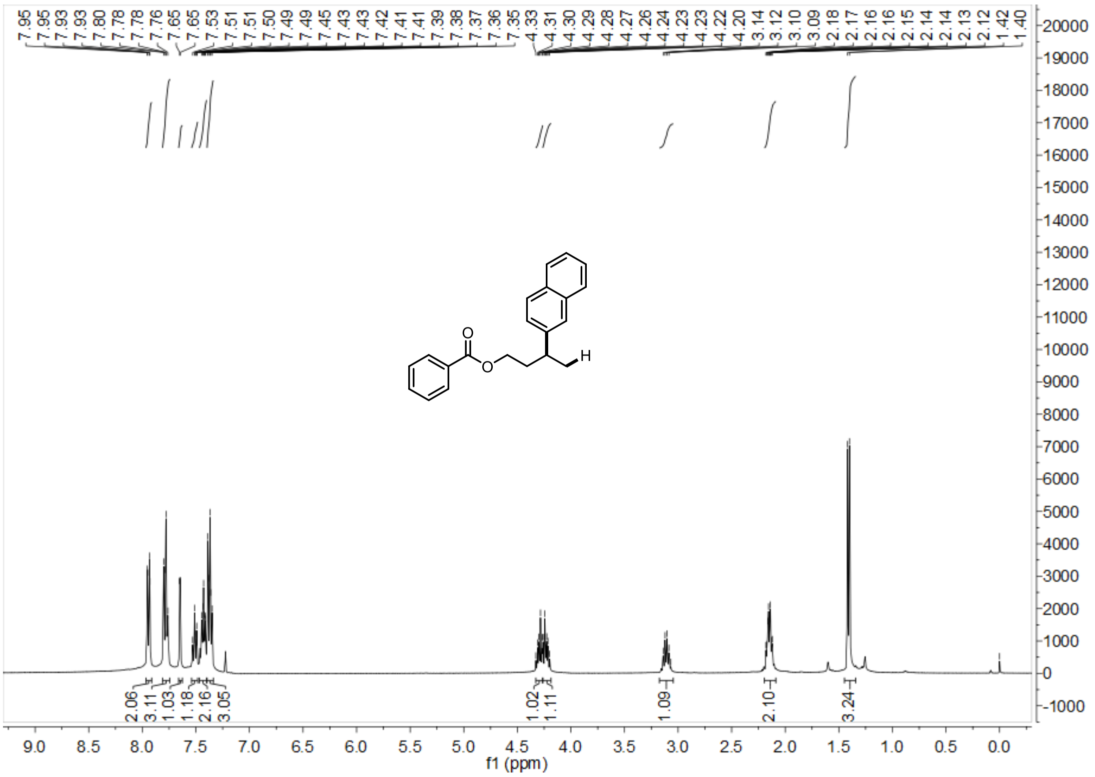

Compound 3ug ^{13}C NMR (101 MHz, $CDCl_3$)

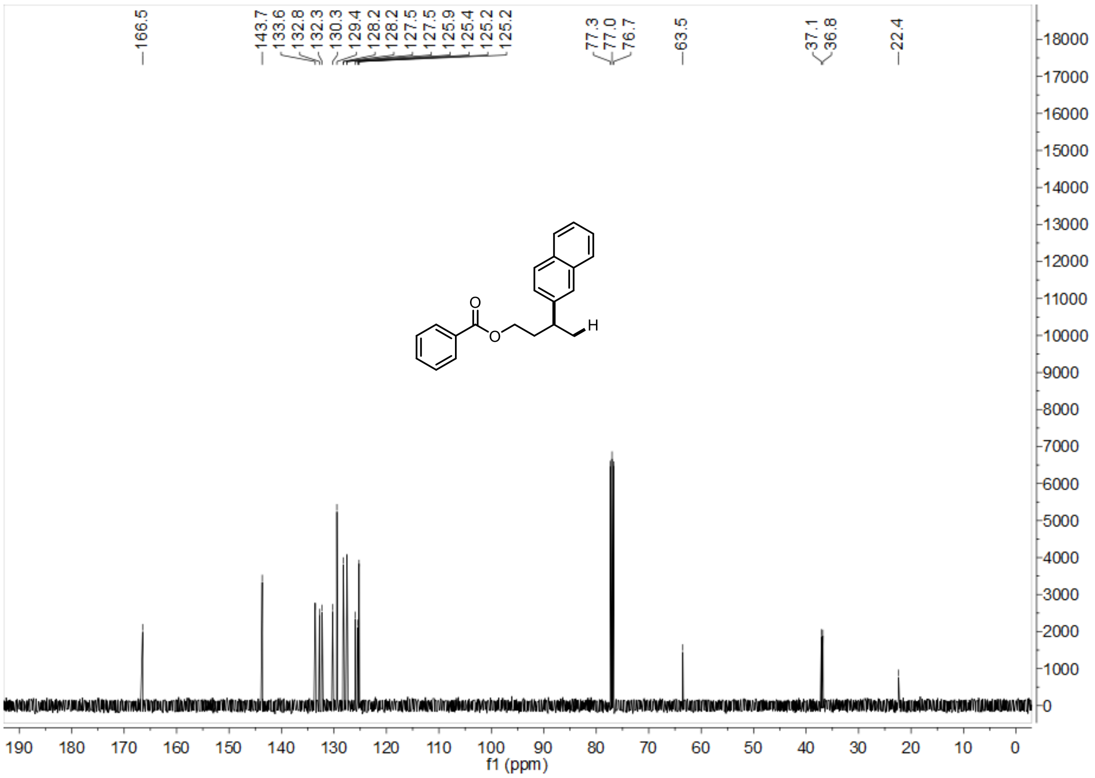

Compound 3vg ^{1}H NMR (400 MHz, $CDCl_3$)

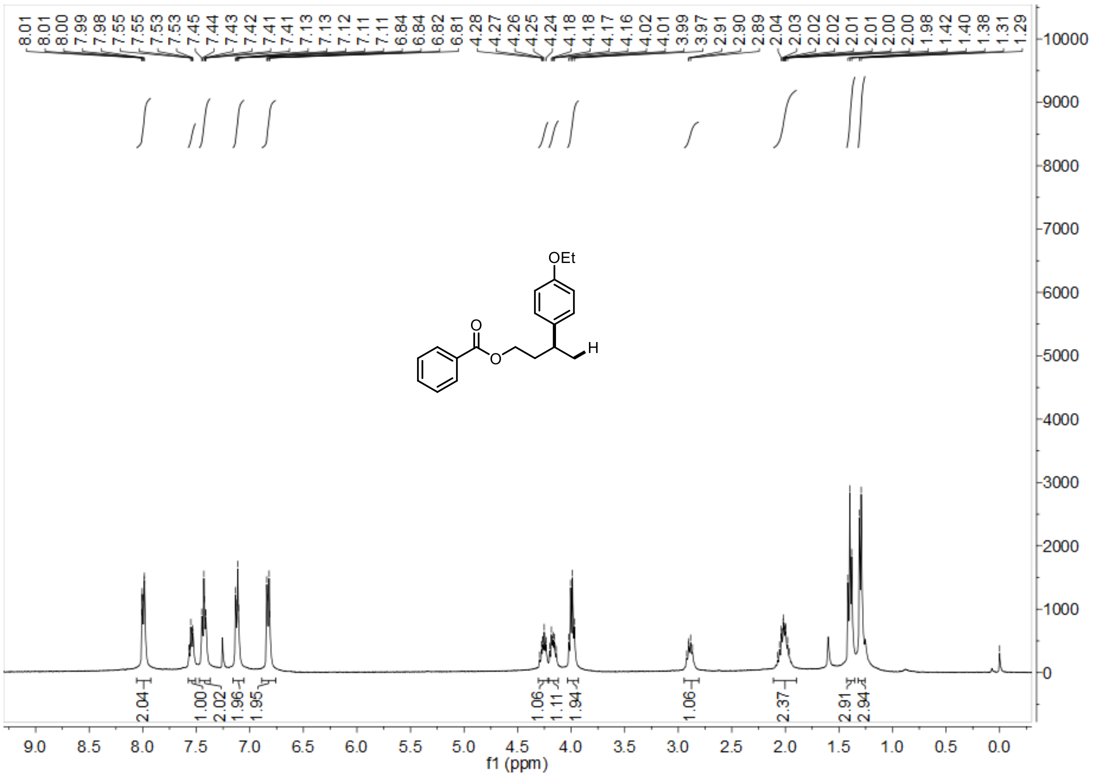

Compound 3vg ^{13}C NMR (101 MHz, $CDCl_3$)

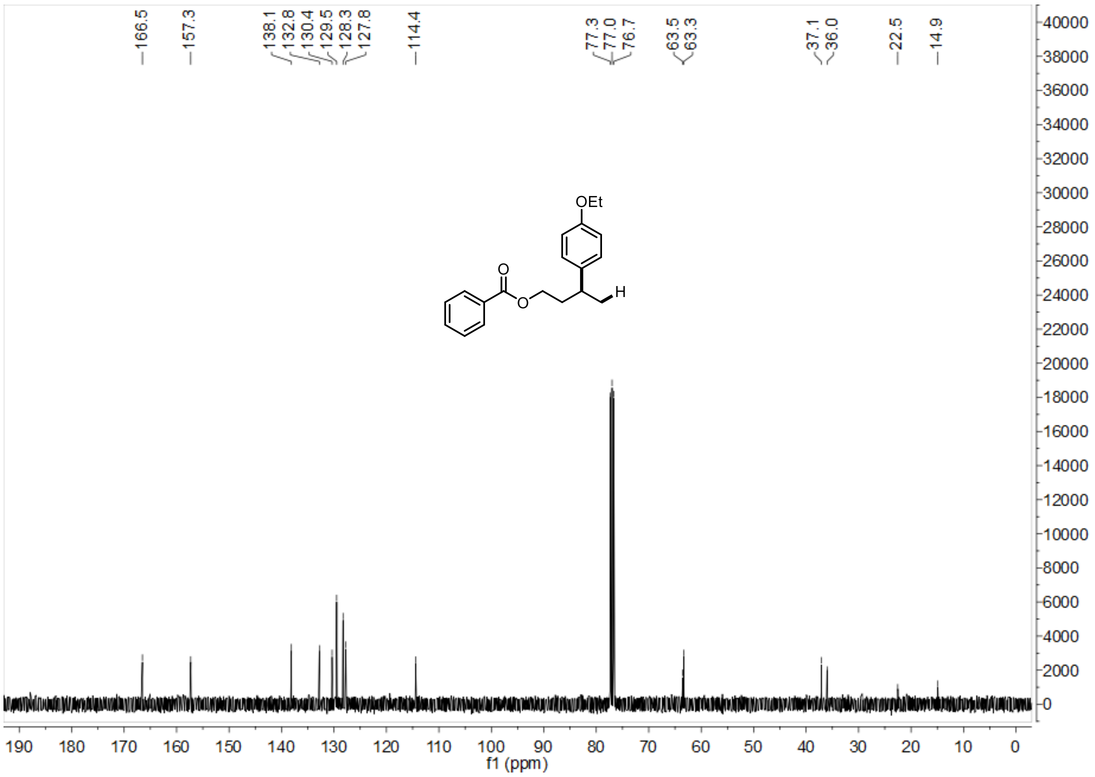

Compound 3wg ^{1}H NMR (400 MHz, $CDCl_3$)

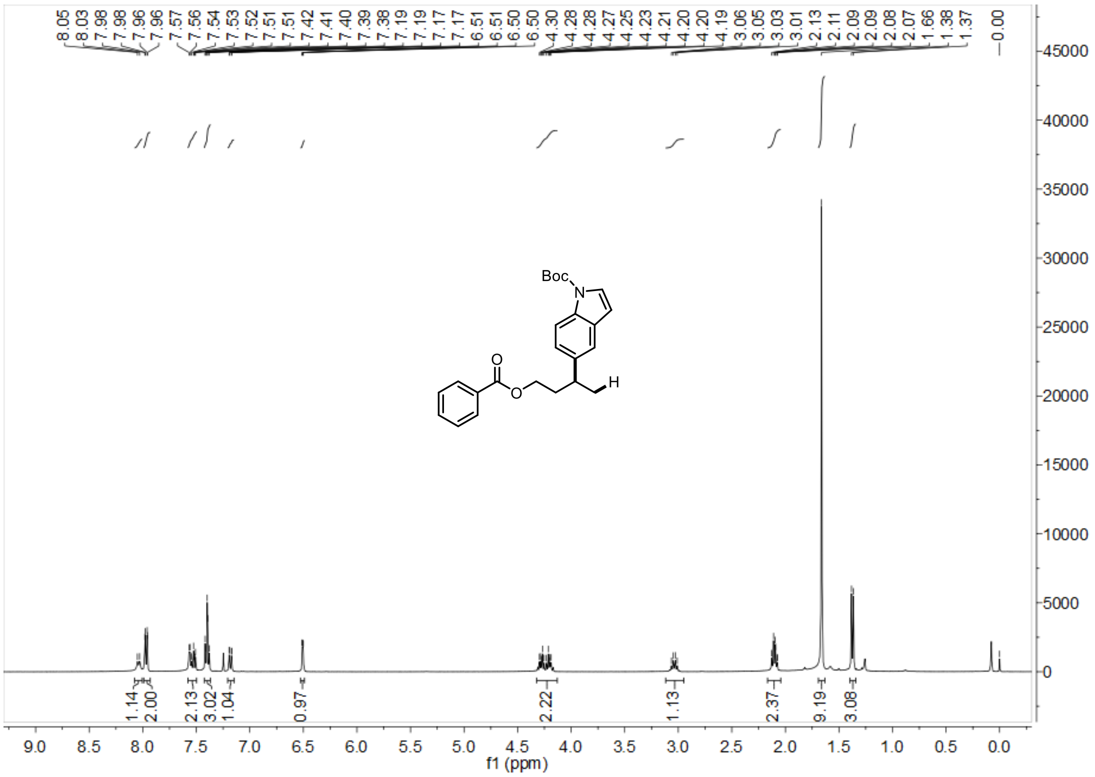

Compound 3wg ^{13}C NMR (101 MHz, $CDCl_3$)

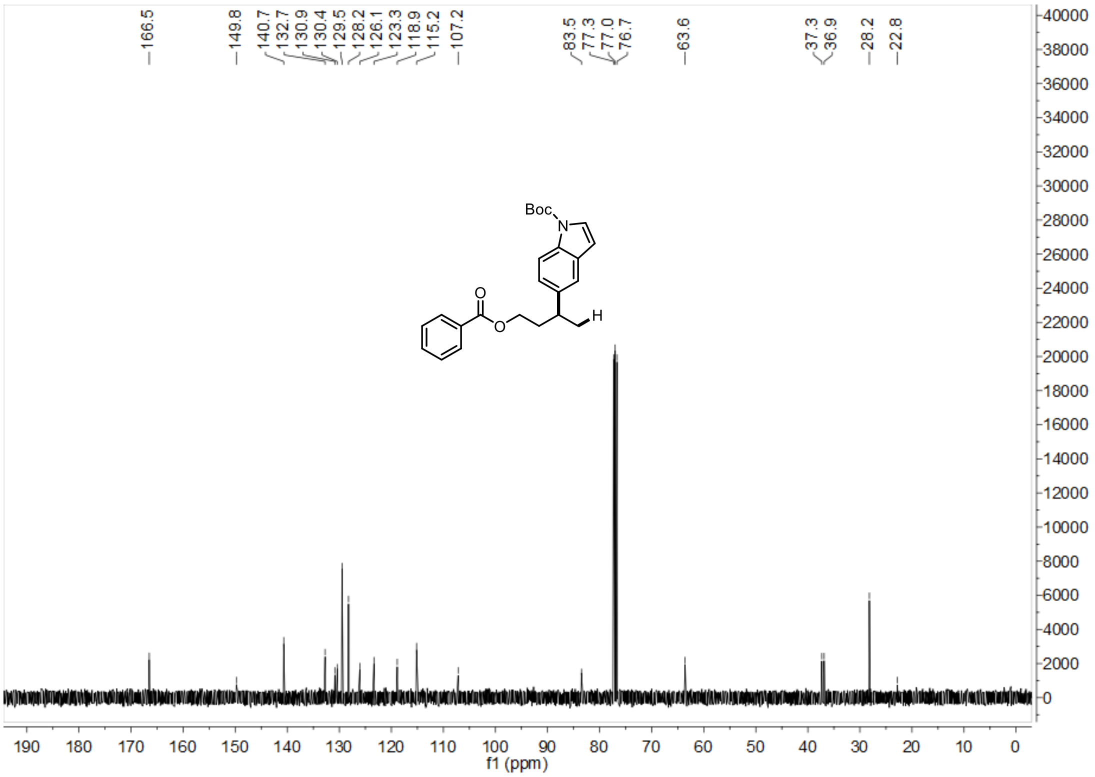

Compound 3xr ^{1}H NMR (400 MHz, $CDCl_3$)

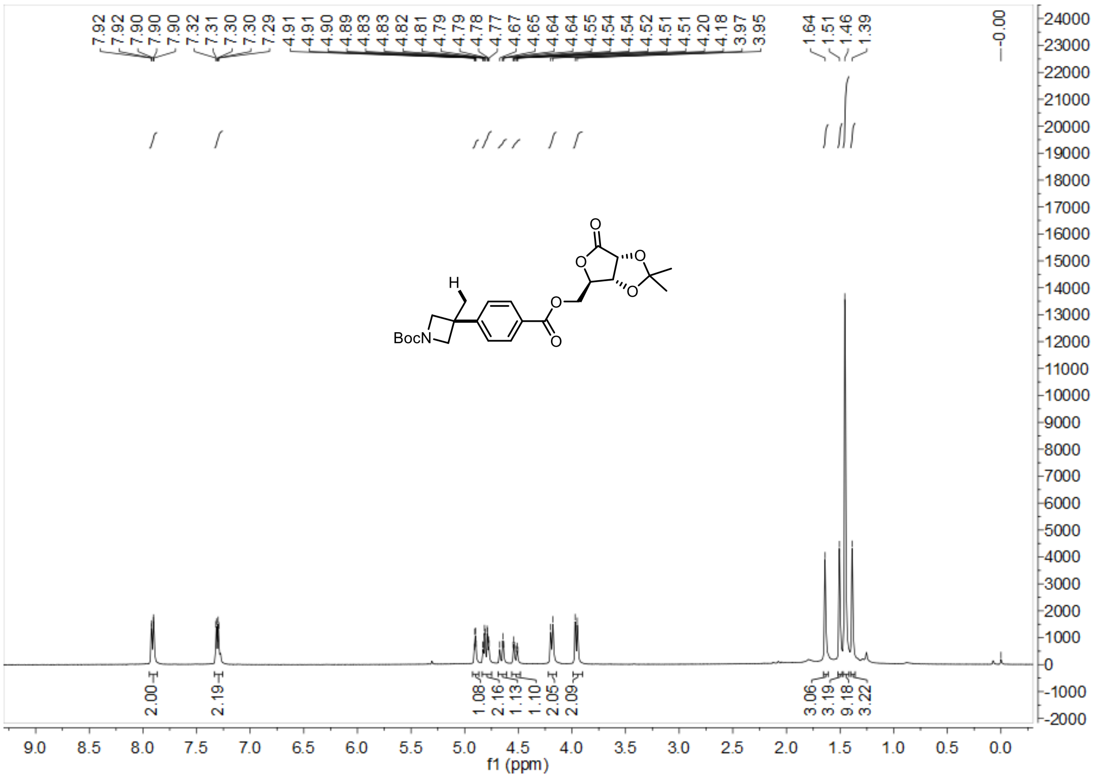

Compound 3xr ^{13}C NMR (101 MHz, $CDCl_3$)

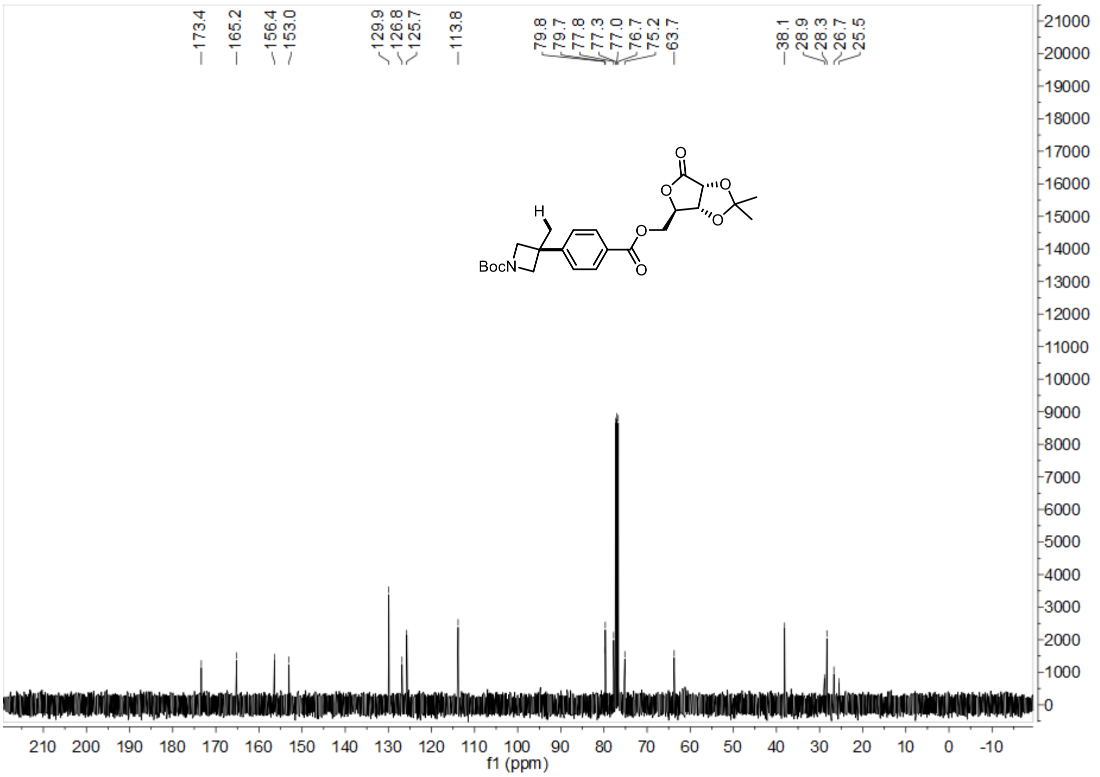

Compound 3yr ^{1}H NMR (400 MHz, $CDCl_3$)

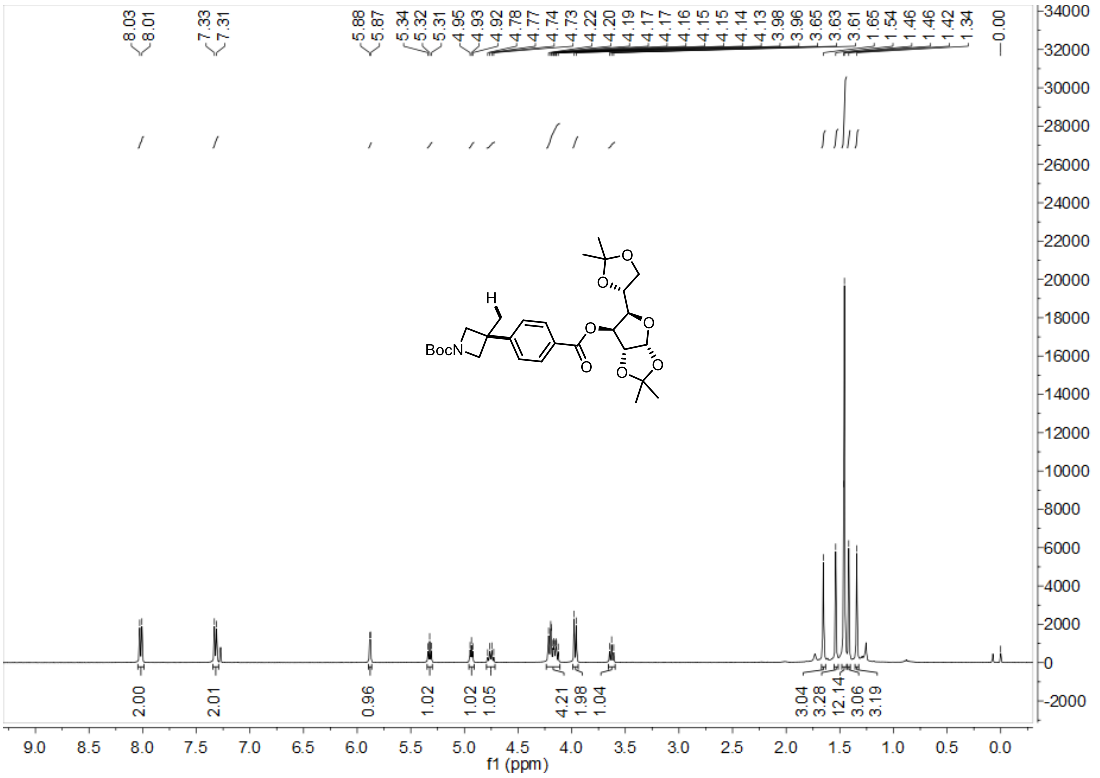

Compound 3yr ^{13}C NMR (101 MHz, $CDCl_3$)

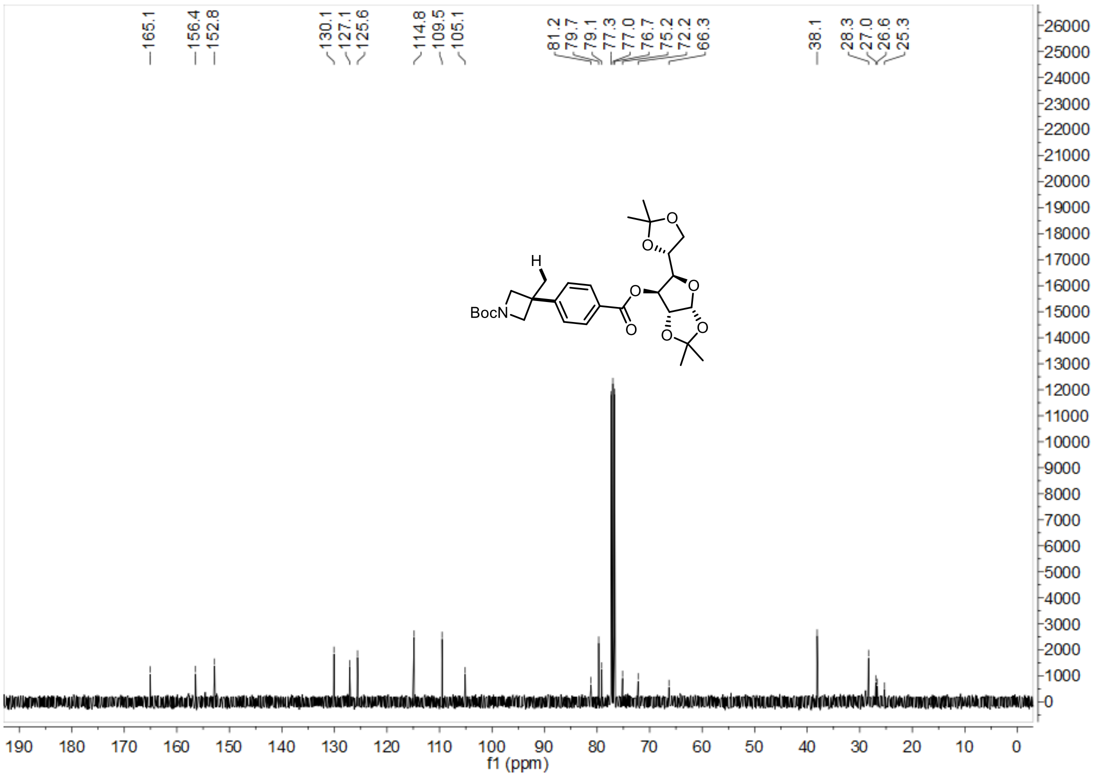

Compound 3zr ^{1}H NMR (400 MHz, $CDCl_3$)

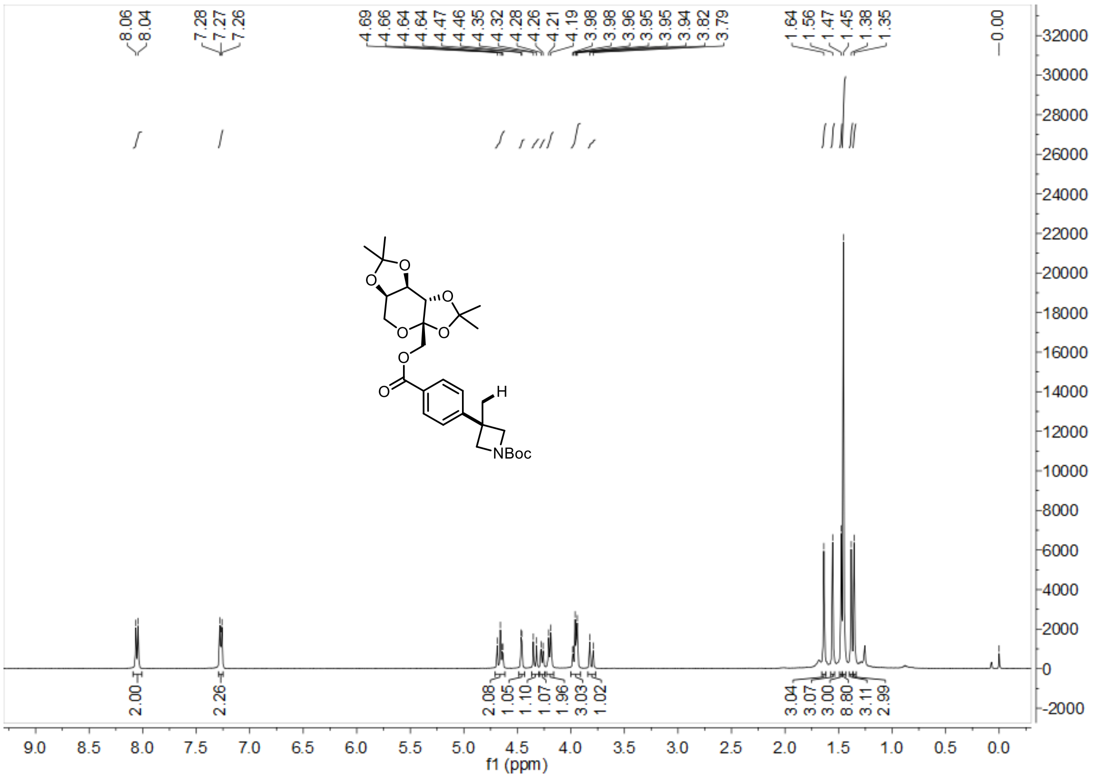

Compound 3zr ^{13}C NMR (101 MHz, $CDCl_3$)

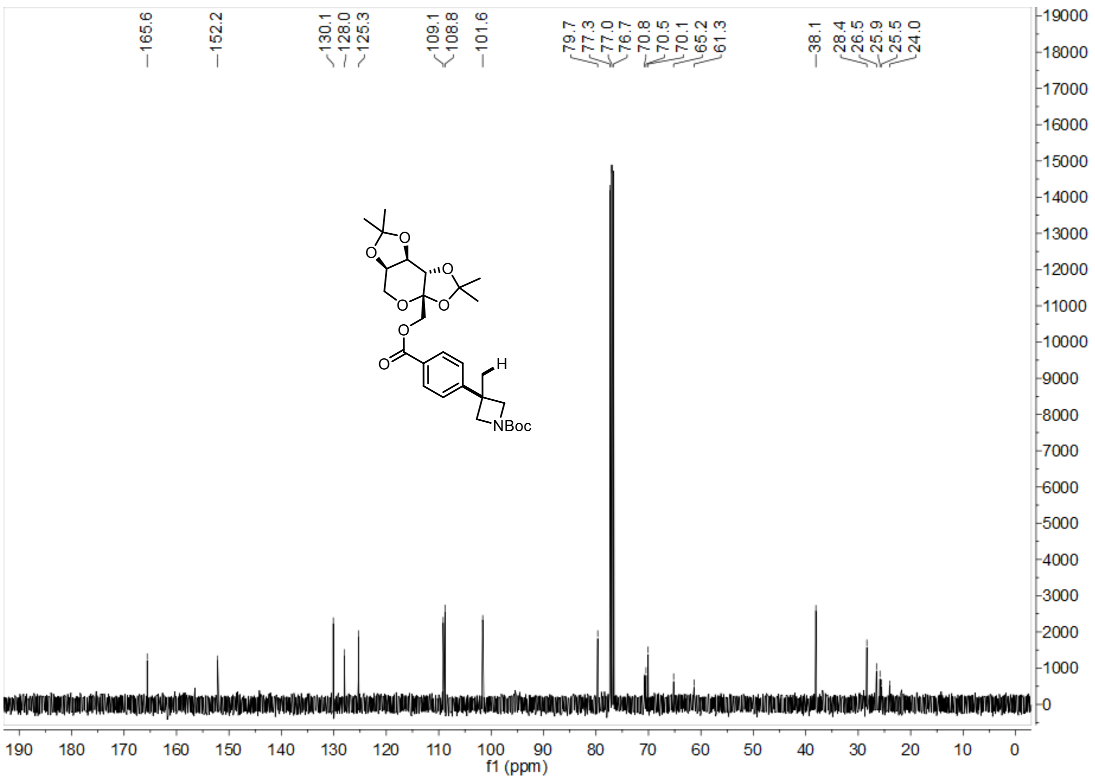

Compound 5 ^{1}H NMR (400 MHz, $CDCl_3$)

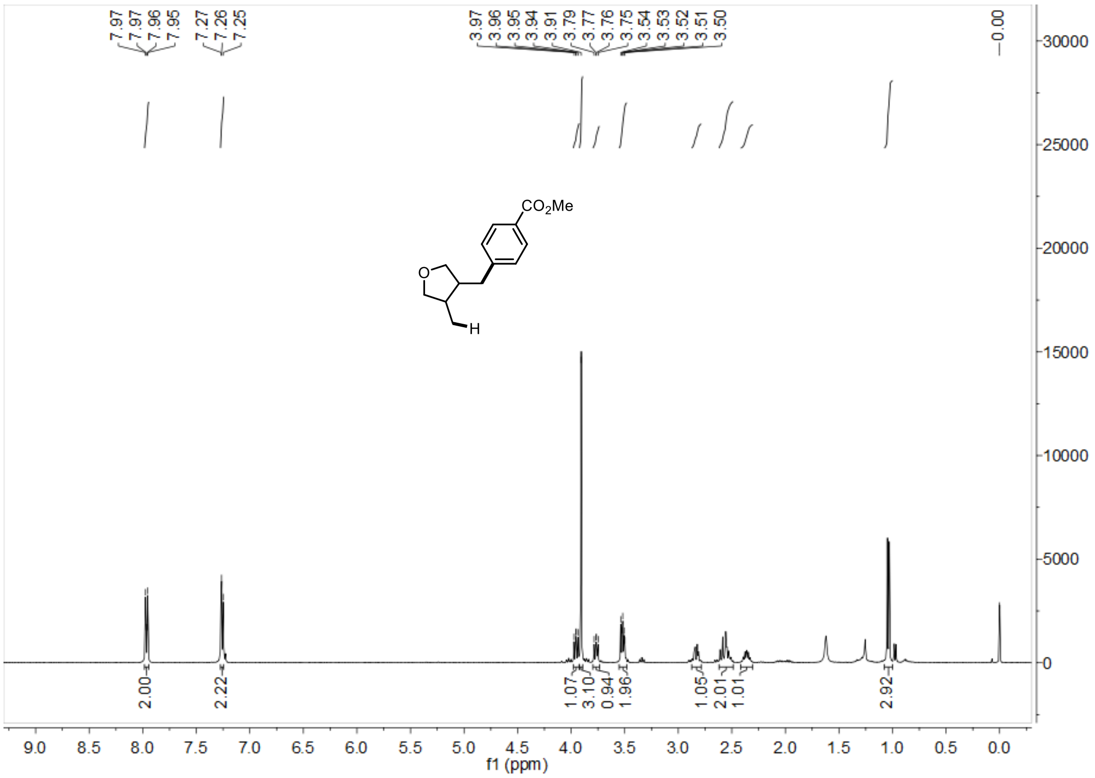

Compound 5 ^{13}C NMR (101 MHz, $CDCl_3$)

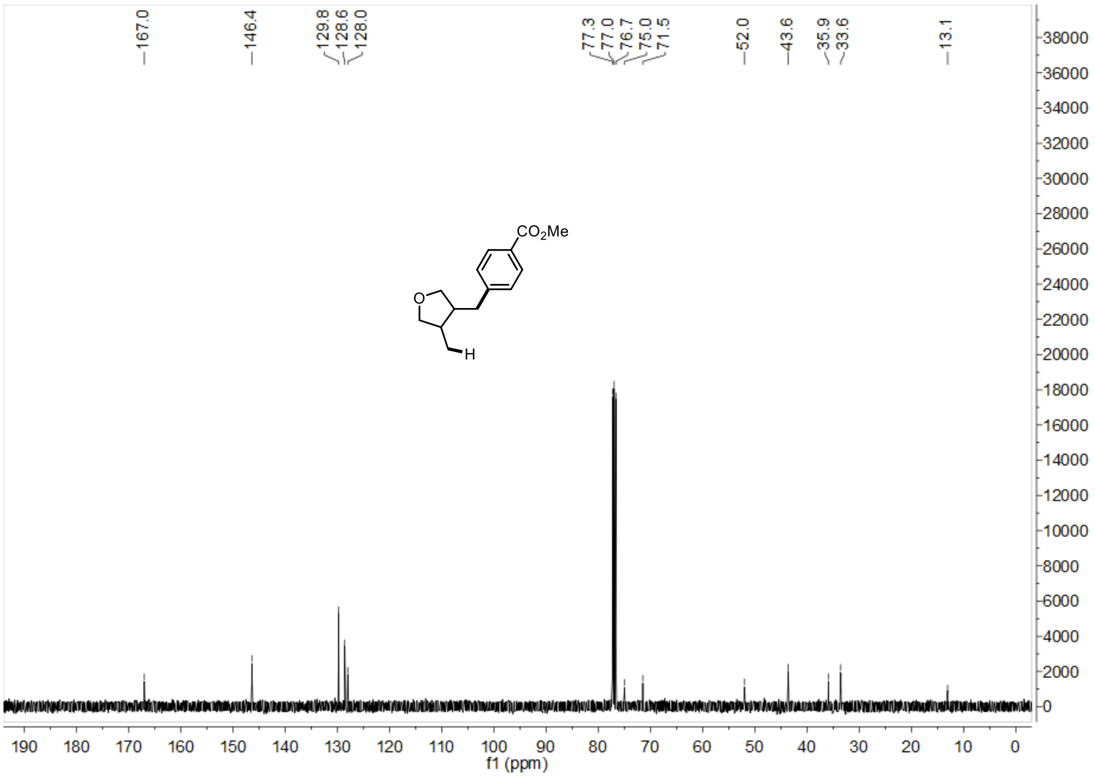

Compound 7 ^{1}H NMR (400 MHz, $CDCl_3$)

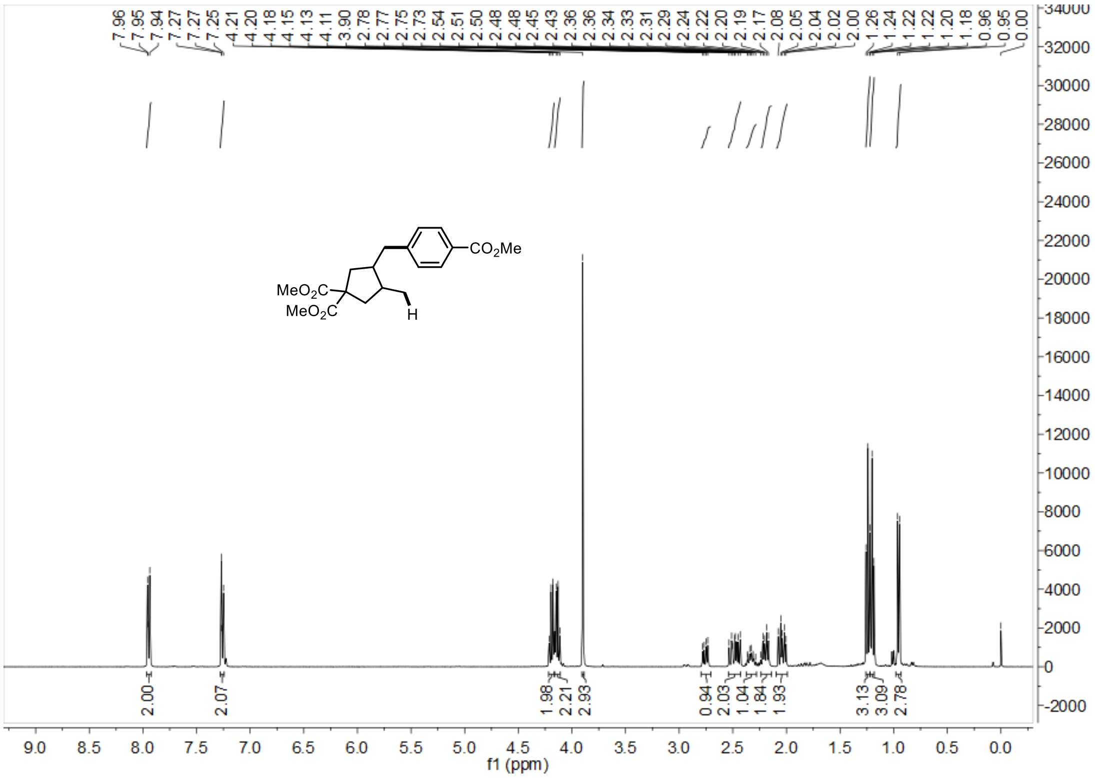

Compound 7 ^{13}C NMR (101 MHz, $CDCl_3$)

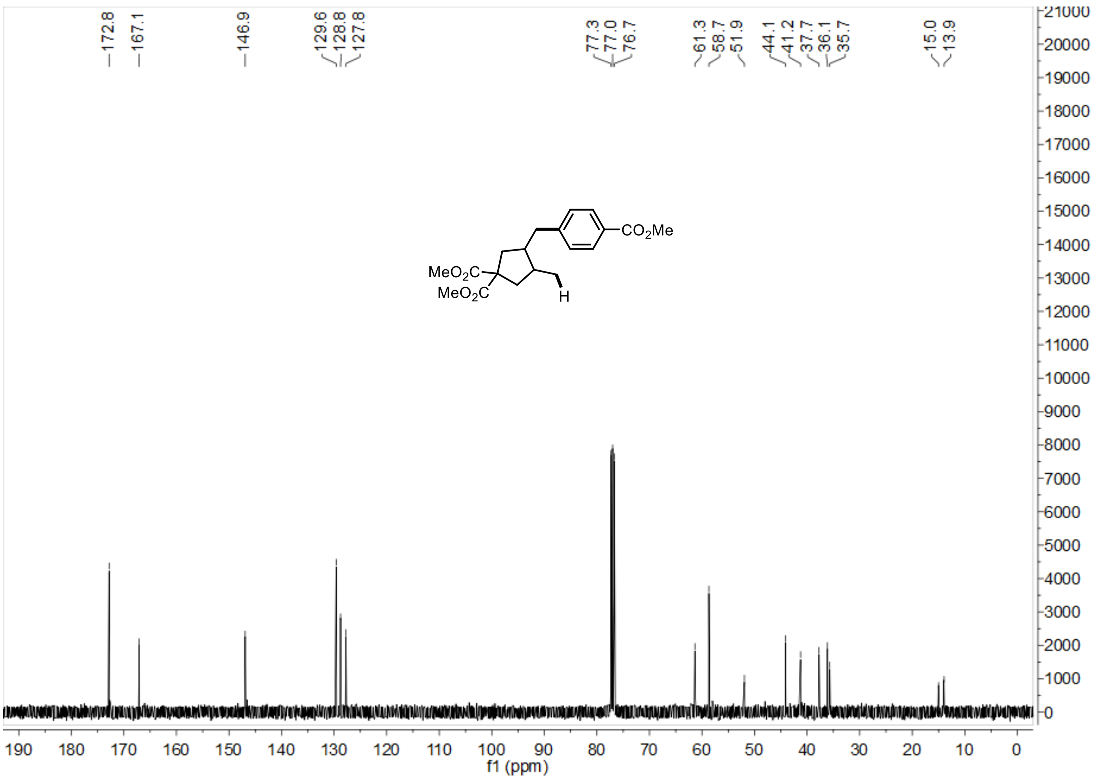

Supplement: SC-015-D4SC03355H-s001 [file SC-015-D4SC03355H-s001.pdf]
